# TNFα modulates PANX1 activation to promote ATP release and enhance P2RX7-mediated antitumor immune responses after chemotherapy in colorectal cancer

**DOI:** 10.1038/s41419-023-06408-5

**Published:** 2024-01-09

**Authors:** Kevin Chih-Yang Huang, Shu-Fen Chiang, Pei-Chun Lin, Wei-Ze Hong, Pei-Chen Yang, Hui-Ping Chang, Shin-Lei Peng, Tsung-Wei Chen, Tao-Wei Ke, Ji-An Liang, William Tzu-Liang Chen, K. S. Clifford Chao

**Affiliations:** 1 Department of Biomedical Imaging and Radiological Science, China Medical University, Taichung, 40402 Taiwan, ROC; 2 Translation Research Core, China Medical University Hospital, China Medical University, Taichung, 40402 Taiwan, ROC; 3 Cancer Biology and Precision Therapeutics Center, China Medical University, Taichung, 40402 Taiwan, ROC; 4 Lab of Precision Medicine, Feng-Yuan Hospital, Ministry of Health and Welfare, Taichung, 42055 Taiwan, ROC; 5 Proton Therapy and Science Center, China Medical University Hospital, China Medical University, Taichung, 40402 Taiwan, ROC; 6 Department of Pathology, Asia University Hospital, Asia University, Taichung, 41354 Taiwan, ROC; 7 School of Chinese Medicine and Graduate Institute of Chinese Medicine, China Medical University, Taichung, 40402 Taiwan, ROC; 8 Department of Colorectal Surgery, China Medical University Hospital, China Medical University, Taichung, 40402 Taiwan, ROC; 9 Department of Radiation Oncology, China Medical University Hospital, China Medical University, Taichung, Taiwan, ROC; 10 Department of Radiotherapy, School of Medicine, China Medical University, Taichung, 40402 Taiwan, ROC; 11 Department of Colorectal Surgery, China Medical University HsinChu Hospital, China Medical University, HsinChu, 302 Taiwan, ROC; 12 School of Medicine, China Medical University, Taichung, 40402 Taiwan, ROC

**Keywords:** Cancer microenvironment, Tumour immunology

## Abstract

ATP and its receptor P2RX7 exert a pivotal effect on antitumor immunity during chemotherapy-induced immunogenic cell death (ICD). Here, we demonstrated that TNFα-mediated PANX1 cleavage was essential for ATP release in response to chemotherapy in colorectal cancer (CRC). TNFα promoted PANX1 cleavage via a caspase 8/3-dependent pathway to enhance cancer cell immunogenicity, leading to dendritic cell maturation and T-cell activation. Blockade of the ATP receptor P2RX7 by the systemic administration of small molecules significantly attenuated the therapeutic efficacy of chemotherapy and decreased the infiltration of immune cells. In contrast, administration of an ATP mimic markedly increased the therapeutic efficacy of chemotherapy and enhanced the infiltration of immune cells in vivo. High PANX1 expression was positively correlated with the recruitment of DCs and T cells within the tumor microenvironment and was associated with favorable survival outcomes in CRC patients who received adjuvant chemotherapy. Furthermore, a loss-of-function P2RX7 mutation was associated with reduced infiltration of CD8^+^ immune cells and poor survival outcomes in patients. Taken together, these results reveal that TNFα-mediated PANX1 cleavage promotes ATP-P2RX7 signaling and is a key determinant of chemotherapy-induced antitumor immunity.

## Introduction

Dying cells play an important role in the initiation of T-cell mediated immunity [1]. The cross-presentation of antigens derived from dying cells enables dendritic cells to present exogenous tissue-restricted or tumor-restricted proteins. This mechanism of cell death is called immunogenic cell death (ICD), and it induces strong immune responses [2]. Cancer cells that die due to immunogenic chemotherapeutics and radiotherapy release inducible and constitutive DAMPs (damage-associated molecular patterns), which are direct upstream drivers of antitumor immunity [3–6].

ATP, which is a DAMP, is increasingly being recognized as an important factor that promotes the engulfment of dying cells [7–9], cross-presentation of tumor antigens to T cells and recruitment of tumor-infiltrating lymphocytes (TILs) [10–12] via several signaling pathways, including NF-kB signaling, RIPK-mediated NLRP inflammasome signaling and caspase-3 gated pannexin 1 (PANX1) channel-mediated signaling [7–9, 13–16]. When activated by the fully assembled NLRP3 inflammasome, caspase-1 sequentially cleaves the proinflammatory cytokines pro-IL-1β and pro-IL-18 into active IL-1β and IL-18, which are then released into the extracellular space to recruit neutrophils [17]. The PANX1-regulated secretion of ATP is associated with a favorable response to chemotherapy [7–9]. The release of cancer-associated ATP via caspase 3-gated PANX1 channels in response to chemotherapeutic drugs also enhances leukocyte recruitment [18, 19] and facilitates dendritic cell (DC) motility via the P2X7 receptor in response to danger signals [20]. Recent studies indicated that tumor growth in mice lacking *P2rx7* does not respond to chemotherapy [21]. Similarly, patients harboring loss-of-function alleles of *P2RX7* (Glu496Ala/rs3751143, 1513A/C) show reduced responses to adjuvant chemotherapy [21]. P2RX7-E496A limits the affinity of P2RX7 for ATP and leads to resistance against ATP-induced cell death in *Mycobacterium*-infected macrophages, confirming the functional effect of this polymorphism on the immune response [22]. Moreover, patients who are homozygous or heterozygous carriers of *P2RX7-E496A* (rs3751143) experienced more rapid relapse than patients who carry the wild-type allele [21]. These findings showed the importance of PANX1-ATP-P2RX7 channels in controlling inflammasome activation, inflammatory cytokine release, and inflammatory cell recruitment [23]. However, the mechanisms underlying this PANX1-ATP-P2RX7 axis in colorectal cancer have not been described.

Tumor necrosis factor (TNF)-mediated cell death can enhance chemotherapeutic drug-induced cell death in colorectal cancer [24]. The results from clinical trials showed that the combination of TNFα with DNA-damaging drugs might result in a therapeutic advantage, suggesting a potential role of TNFα after the induction of DNA damage [25]. Moreover, TNFα also contributes to immune surveillance via cytotoxic lymphocytes [26, 27]. Tumor immune evasion can arise through the loss of TNFα sensitivity, independent of perforin-mediated killing mechanisms. Therefore, TNFα-mediated cell killing might be a potent mechanism for enhancing tumor cell eradication [27]. When caspase-mediated apoptosis is inhibited, necroptosis is activated by a series of essential signaling molecules, such as RIPK1, RIPK3, and MLKL. RIPK3 phosphorylates MLKL, causing its oligomerization and insertion into the cellular membrane, which is followed by the lethal permeabilization of the plasma membrane to ATP, which in turn targets the purinergic receptor P2RX7 to mediate immunostimulatory signals [28]. Furthermore, PANX1 is a direct target of the TNFα signaling pathway [13, 18]. However, the detailed molecular mechanisms by which TNFα-mediated ATP release enhances antitumor immunity in colorectal cancer are still unknown.

In the present study, we found that TNFα promoted PANX1 cleavage through TNFR1 in a caspase 3-dependent manner in colorectal cancer. TNFα enhanced chemotherapeutic drug-induced cell death and increased PANX1-mediated caspase-3 cleavage to promote ATP release. Low PANX1 expression in cancer cells resulted in the release of lower ATP levels in response to chemotherapeutic drug treatment and immunogenicity that was insufficient for promoting dendritic cell maturation, proinflammatory cytokine release, and T-cell-mediated killing in vitro. Moreover, inhibition of the PANX1-ATP-P2RX7 axis by a small molecule inhibitor significantly increased tumor volume after chemotherapy treatment and reduced dendritic cell maturation and T-cell infiltration. However, the combination of this inhibitor with a ATP mimic markedly increased the response to chemotherapy, delayed tumor growth, enhanced dendritic cell maturation and promoted the recruitment of immune cells. Patients with low PANX1 expression in cancer cells or patients carrying P2RX7 polymorphism (*E496A*/rs3751143) had a better response to adjuvant chemotherapy associated with unfavorable survival outcomes. These results indicated that ATP-mediated P2RX7 activation via TNFα-mediated PANX1 channels was essential for chemotherapy-induced antitumor immunity in CRC.

## Materials and methods

### Cell culture, treatment, and western blotting analysis

The HCT15, HCT116, SW620, SW480 and LoVo colorectal cancer cell lines were obtained from the American Type Culture Collection (ATCC, VA, USA). The cells were cultured and maintained in RPMI 1640 medium supplemented with 10% fetal bovine serum (Life Technologies, Grand Island, New York, USA), 2 mM glutamine, 100 U/ml penicillin, 100 mg/ml streptomycin, and 1 mM sodium pyruvate at 37 °C in a humidified, 5% CO_2_ atmosphere. HCT15 and HCT116 cells were seeded in a 10 cm dish at ~80% confluence in RPMI 1640 supplemented with 10% FBS the day before treatment. Total cell lysates (30 μg) were separated using sodium dodecyl sulfate‒polyacrylamide gel electrophoresis (SDS‒PAGE; 6–12% resolving gel) and were electrophoretically transferred to PVDF membranes (GE, Amersham, UK). The membranes were blocked with 5% nonfat milk and probed with specific antibodies overnight at 4 °C. Then, horseradish peroxidase (HRP)-conjugated secondary antibodies (1:10,000, GE Healthcare, Amersham, UK) were added to the membranes, followed by detection using the Immobilon Western Chemiluminescent HRP Substrate (Millipore, MA, USA). The densitometric analysis of the western blots was performed using the ImageQuant™ LAS 4000 biomolecular imager (GE Healthcare, Amersham, UK). The digital images were processed with Adobe Photoshop 7.0 (Adobe Systems, CA, USA). Each blot was stripped using Restore Western Blot Stripping Buffer (Pierce, IO, USA) and incubated with the other antibodies. The results were assessed using ImageJ software (NIH, MD, USA). The antibodies used were as follows: anti-TNFR1 (A12540, ABclonal), anti-TNFR2 (A12536, ABclonal), anti-caspase-3 (#9662, Cell Signaling Tech. and IR96-401, iREAL Bio.), anti-cleaved caspase-3 (#9661 Cell Signaling Tech.), anti-PANX1 (ab233479, Abcam and #91137, Cell Signaling Tech.), anti-caspase-8 (SC-81656, Santa Cruz), anti-phospho-Src family kinase (SFK^Y416^, #2101, Cell Signaling Tech. and AP0511, ABclonal), anti-RIPK1 (#4926, Cell Signaling Tech.), anti-RIPK3 (#10188, Cell Signaling Tech.), anti-MLKL (#14993, Cell Signaling Tech.) and anti-phospho-MLKL^S358^ (#91689, Cell Signaling Tech.) antibodies.

### ATP assay

Cells were treated with TNFα (50 ng/mL) or oxaliplatin (25 μM), and intracellular ATP was measured at the indicated time points. Briefly, cells were washed with phosphate-buffered saline and stained with quinacrine (5 μM × 5 min, Sigma), an acridine derivative that has a very high affinity for ATP and is utilized to label intracellular ATP levels, placed in isotonic buffer and counterstained with propidium iodide (PI) for flow cytometry. For extracellular ATP levels, the conditioned medium was harvested and analyzed with a luminescent ATP detection kit (ab113849, Abcam) according to the manufacturer’s protocol.

### Assessment of cell growth and apoptosis

Cell growth was assessed using a CCK-8 assay. Apoptosis and necrosis were assessed using an apoptosis/necrosis detection kit (Enzo Life Sciences, Plymouth Meeting, USA) [29]. According to the instructions provided with the kit, the apoptotic and necrotic cells were fluorescently labeled with annexin V-EnzoGold and PI, respectively. The cells (2000) were observed to assess apoptosis or necrosis under a fluorescence microscope. The experiments were performed in triplicate.

### qRT–PCR

Total RNA was extracted from cell lines with TRIzol (Invitrogen, CA, USA), quantified by measuring the absorbance at 260 nm, and then reverse transcribed into cDNA using iScript™ Reverse Transcription Supermix (Bio-Rad, CA, USA) according to the manufacturer’s instructions. Primers were designed using the Primer design tool (NCBI, USA) according to target sequence information from the NCBI database. qRT–PCR was performed in a final reaction volume of 20 μL with iQ™ SYBR® Green Supermix (Bio-Rad, CA, USA) using the CFX96 Touch Real-Time PCR Detection System (Bio-Rad). All the reactions were performed in triplicate for each sample, and GAPDH was used as a reference gene for normalization. The relative gene expression levels were calculated using the 2^−ΔΔCt^ method. The gene expression levels were compared using the *t test*.

### Evaluation of immune cell profiles in PANX1-deficient tumor-bearing animals in response to OXP

BALB/c mice (female, 4 weeks old, BioLASCO Taiwan Co, Taipei, Taiwan) were maintained under specific pathogen-free conditions in a temperature-controlled environment with 12 h light/12 h dark cycles, and they received food and water ad libitum according to the institutional guidelines approved by the China Medical University Institutional Animal Care and Use Committee. CT26^shNC^ and CT26^shPANX1^ cells (5 × 10^5^ cells/mouse) were suspended in 100 μl of 50% *Matrigel* and subcutaneously injected into the left leg of each BALB/c mouse. On Day 7, when the tumor sizes reached 100 mm^3^, the animals were administered OXP (50 mg/kg OXP) on Days 7, 12, and 19. The tumor volume was measured every 3 days, and the mice were sacrificed on Day 26. The tumor volumes were calculated according to the following formula: (width^2^ × length)/2. The mice were sacrificed at the end of the experiments, and the resected tumor tissues were collected for immune cell analysis by flow cytometry.

### Flow cytometry analysis of immune cell profiles

Tumors were dissected from the mice, weighed, and then placed in Petri dishes containing blank RPMI media at room temperature to prevent dehydration. Tumors were minced into small pieces (1–2 mm) with a beaver blade, filtered through a 70-μm strainer, centrifuged, and then resuspended in blank RPMI media. Thereafter, the cell suspensions were layered over Ficoll-Paque media and centrifuged at 1025 × *g* for 20 min. The layer of mononuclear cells was transferred to a conical tube, 20 ml of complete RPMI media was added and gently mixed, and the sample was centrifuged at 650 × *g* for 10 min twice. Finally, the supernatant was removed, and the TILs were resuspended in complete RPMI media.

Then, the TILs were resuspended in 500 μL of staining buffer (2% BSA and 0.1% NaN_3_ in PBS). The cells were stained with different antibody panels against combinations of surface markers: [1] CD8^+^ T cells: CD45-PE (E-AB-F1136UD, Elabscience, Texas, USA) and CD8a-PerCP (E-AB-F1104UF, Elabscience, Texas, USA); [2] Foxp3^+^ regulatory T cells: CD45-PE (E-AB-F1136UD, Elabscience, Texas, USA), CD4-APC (E-AB-F1097UE, Elabscience, Texas, USA), CD25-PerCP (E-AB-F1194J, Elabscience, Texas, USA), and Foxp3-FITC (E-AB-F1238C, Elabscience, Texas, USA). For intracellular staining, the TILs were fixed and permeabilized with a Foxp3/transcription factor staining buffer set (eBioscience, Thermo Fisher, CA, USA) after cell surface staining. The cells were then stained with Foxp3-FITC for 45 min. The samples were washed twice with Perm Wash Buffer and then analyzed by a BD Canto II flow cytometer (BD, CA, USA). Isotype controls, including PerCP-conjugated rat IgG2b κ isotype control (E-AB-F09842J), APC-conjugated rat IgG2b (E-AB-F09843E, Elabscience, Texas, USA), and PE-conjugated rat IgG2b κ isotype control (E-AB-F09842D, Elabscience, Texas, USA), were used.

### Treatment of tumor-bearing animals with an ATP antagonist and agonist after OXP-based chemotherapy

CT26 cells (5 × 10^5^ cells/mouse) were suspended in 100 μl of 50% *Matrigel* matrix and subcutaneously injected into the left leg of each BALB/c mouse. On Day 7, when the tumor sizes reached 100 mm^3^, the animals were randomly assigned to 4 groups receiving FOLFOX (20 μM levofolinate, 20 μM 5-Fu, 100 μM OXP, intraperitoneal injection) or not, in combination or not with the ATP agonist BzATP (20 μM, 1 h before and after FOLFOX by intraperitoneal injection) or the ATP antagonist A438079 (20 μM, 1 h before and after FOLFOX by intraperitoneal injection). The tumor volume was measured every 3 days, and the mice were sacrificed on Day 26. The tumor volumes were calculated according to the following formula: (width^2^ × length)/2. The mice were sacrificed at the end of the experiments, and tumor tissues from the representative mice were collected for lysis, subjected to western blotting analysis and stained by immunohistochemistry.

### Evaluation of the immunomodulatory effect of BzATP in response to OXP in DC-depleted tumor-bearing animals

CT26 cells (5 × 10^5^ cells/mouse) were suspended in 100 μl of 50% *Matrigel* matrix and subcutaneously injected into the left leg of each BALB/c mouse. On Day 7, when the tumor sizes reached 100 mm^3^, the animals were randomly assigned to five groups receiving OXP (50 mg/kg, intraperitoneal injection) or not, in combination or not with clodronate liposomes (50 μl/mouse, intraperitoneal injection) on the indicated days. The tumor volume was measured every 3 days, and the mice were sacrificed on Day 26. The tumor volumes were calculated according to the following formula: (width^2^ × length)/2. The mice were sacrificed at the end of the experiments, and tumor tissues were collected for flow cytometry analysis.

### Construction of the tissue microarray (TMA)

Tissue microarrays were constructed from 156 surgical tumor tissues and adjacent normal tissues from stage III colorectal cancer patients [30]. The tumor cell areas were evaluated and marked on hematoxylin and eosin (H&E)-stained slides, and the corresponding area on the paraffin block (donor block) was then identified and moved by an AutoTiss 10C system (EverBio Technology Inc., Taipei, Taiwan) to a recipient block. Each punch was 2 mm in diameter, and a maximum of 60 punches were placed on a single block to generate sections with a microtome.

### Immunohistochemistry

Immunohistochemistry (IHC) was performed on 3-μm-thick histological TMA sections [31]. TMA sections were stained individually with horseradish peroxidase-conjugated avidin biotin complex (ABC) using the Vectastain Elite ABC-HRP Kit (PK-6100, Vector Laboratories, Burlingame, CA, USA) and DAB chromogen (Vector Laboratories, Burlingame, CA) and counterstained with hematoxylin [32]. Staining for CD8 (1:500 for 2 h at room temperature, ab4055, Abcam, Cambridge, UK), CD11c (ab52632, Abcam, Cambridge, UK) and PANX1 (1:500 for 2 h at room temperature, ab233479, Abcam, Cambridge, UK) was considered positive when these signals were detected in the cytoplasm or at the cell membrane of tumor-infiltrating lymphocytes (TILs), and staining was evaluated using microscopy (OLYMPUS BX53, Tokyo, Japan) to assess the density of CD8^+^ TILs. Two pathologists, blinded to all the information about the samples, evaluated the CD8^+^ TILs. To detect CD8^+^ TILs, the tissues were reviewed at 40× magnification, and the area with the highest density of CD8^+^ TILs adjacent to malignant cells was counted at 400× magnification (no. of CD8+ TILs/high-power field). The average number of CD8^+^ TILs in five high-power fields was included in the evaluation. For CD8^+^ T-cell scoring, a count of zero CD8^+^ TILs in a high-power field was given a score of 0, a count of 1-3 CD8^+^ TILs was given a score of 1+, a count of 4-10 CD8^+^ TILs was given a score of 2+, and a count of >10 CD8^+^ TILs was given a score of 3+.

### Genomic DNA extraction and SNP genotyping

Genotyping of SNPs was performed using the iPLEX^®^ HS panel on the MassARRAY^®^ System (Agena Bioscience, San Diego, CA, USA), which uses matrix-assisted laser desorption/ionization time-of-flight mass spectrometry for amplicon detection (MALDI-TOF-MS; SpectroACQUIRE, Agena Bioscience). Primers designed for PCR (polymerase chain reaction) amplification of specific polymorphisms in TLR5-R392STOP (rs5744168), TLR5-F616L (rs5744174), P2RX7-E496A (rs3751143), and TLR2 −196–−174 (rs111200466) and extension reactions were prepared using MassARRAY^®^ Assay Design Version 3.1 software (Agena Bioscience, San Diego, CA, USA).

Genomic DNA was extracted from two 3-μm-thick FFPE sections of nontumor tissues from colon carcinoma patients using a QuickExtract™ FFPE DNA Extraction Kit (QEF81050, Epicenter, WI, USA). For SNP genotyping, 10 ng of total genomic DNA was used for PCR amplification. The PCR samples contained *Taq* DNA polymerase (Agena Bioscience), genomic DNA (5–10 ng), PCR primers, and dNTPs. Following standard protocols for PCR (95 °C 5 s→58 °C 15 s→68 °C 30 s, 45 cycles), the remaining dNTPs were removed by the addition of alkaline phosphatase (Agena Bioscience, San Diego, CA, USA), and the plates were subsequently incubated at 37 °C for 40 min.

Following PCR, SAP addition, and the iPLEX HS^®^ extension reaction, the samples were desalted by resin treatment for 15 min, spotted onto SpectroCHIP^®^ Arrays (Agena Bioscience, San Diego, CA), analyzed by mass spectrometry, and ultimately interpreted using SpectroTYPER v4.0 software (Agena Bioscience, San Diego, CA).

### Statistical analysis

SAS statistical software, version PC 9.4 (SAS Institute, NC, USA), was used to perform the statistical analyses. All tests reported two-sided *p* values with the significance level set at 0.05. Student’s *t* test, Pearson’s chi-square and Fisher’s exact test were used to perform group comparisons. Cox regression analysis was used to estimate the hazard ratios (HRs) and 95% confidence intervals (CIs) for univariate and multivariate models. Influential factors that were correlated with the rectal cancer patient survival rate were adjusted in the Cox models, including age (≥65 versus <65), pT stage (tumor grade 3–4 versus tumor grade 1–2), tumor location (proximal colon versus distal colon), lymphovascular invasion (present versus absent), perineural invasion (present versus absent), CD8^+^ TILs (high versus low) and P2RX7 (variant vs. WT). The Kaplan‒Meier estimation method was used to assess the 5-year overall survival and disease-free survival. Survival time was defined as the time from diagnosis until relapse or death. Univariate comparisons were performed using log-rank tests.

## Results

### TNFα activates caspase-dependent PANX1 cleavage to promote cell death via TNFR1 in colorectal cancer

**Fig. 1:**
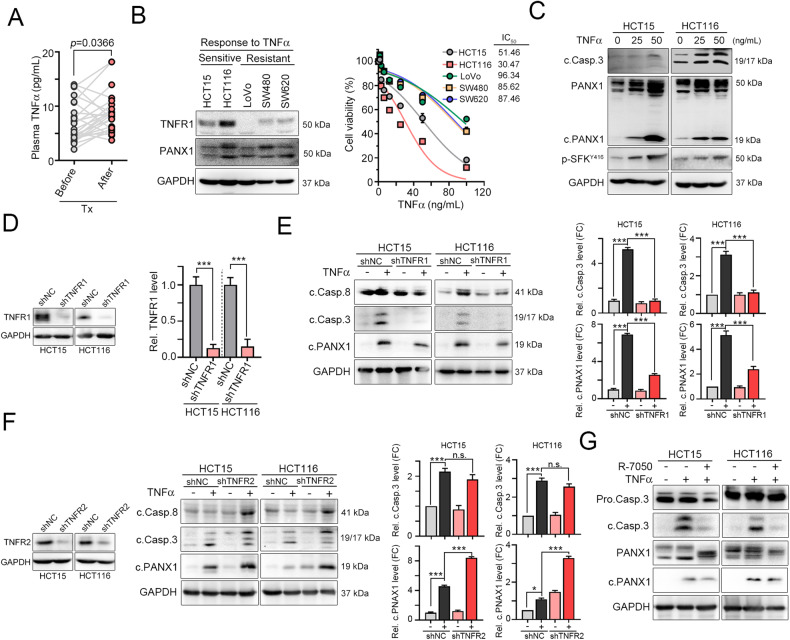
PANX1 is cleaved by caspase activation in TNFα-sensitive CRC cells. **A** The plasma TNFα levels in CRC patients before and after chemotherapy treatment were measured by ELISA (*n* = 19, *p* = 0.0366, paired t-test). **B** The response to TNFα in five CRC cell lines was evaluated by cell viability assay after 24 h of treatment (*n* = 3). Among the five cell lines, two (HCT15 and HCT116) were sensitive to TNFα treatment. **C** HCT15 and HCT116 cells were treated with TNFα (25 and 50 ng/mL) for 24 h, and cell lysates were harvested for western blotting. **D** HCT15 and HCT116 cells were infected with lentivirus carrying shRNA targeting TNFR1 and selected with puromycin for 3 days. The knockdown efficacy was evaluated by western blotting (*n* = 3). ***p < 0.001. Unpaired t-test. **E** HCT15^shNC^ and HCT15^shTNFR1^ cells were treated with TNFα (50 ng/mL) for 24 h. The cleavage of caspase-8, caspase-3, and PANX1 was evaluated by immunoblotting (*n* = 3). Similar experiments were carried out in HCT116 cells (*n* = 3). ***p < 0.001. One-way ANOVA test. **F** HCT15 and HCT116 cells were infected with lentivirus carrying shRNA targeting TNFR2 and selected with puromycin for 3 days. The knockdown efficacy was evaluated by immunoblotting (*n* = 3). HCT15^shNC^ and HCT15^shTNFR2^ cells were treated with TNFα (50 ng/mL) for 24 h. The cleavage of caspase-8, caspase-3, and PANX1 was evaluated by western blotting (*n* = 3). Similar experiments were carried out in HCT116 cells (*n* = 3). *p < 0.05 and ***p < 0.001. One-way ANOVA test. **G** HCT15 and HCT116 cells were treated with TNFα (50 ng/mL) and the TNFR1 inhibitor R-7050 (10 μM) for 24 h. The cleavage of caspase-8, caspase-3, and PANX1 was evaluated by western blotting.

The TNFα that is present in the tumor microenvironment (TME) has the potential to activate cell death pathways in colorectal cancer cells [24, 33]. After chemotherapy, we found that the plasma level of TNFα was significantly upregulated in colorectal cancer (Fig. 1A) and that the level of *TNFα* was markedly associated with survival outcome in The Cancer Genome Atlas (TCGA) colon adenocarcinoma database (Fig. S1A, *n* = 597, log-rank *p* = 0.0409). Recent studies showed that loss of TNFα sensitivity led to tumor immune evasion and was associated with reduced infiltration of immune cells [34], suggesting that TNFα-mediated cell death was essential for promoting antitumor immunity. RIPK-dependent necroptosis and caspase-dependent apoptosis might be involved in ATP release in response to TNFα [35]. Therefore, we first examined the expression of necroptosis-related proteins such as RIPK1, RIPK3, and MLKL (RIPK-mediated ATP release) and the expression of caspase-8/PANX1 (PANX1-mediated ATP release) [16, 18, 34]. As shown in Fig. 1B and S2A, we found that TNFR1 was highly expressed in HCT116 and HCT15 cells, expressed at low levels in SW480 and SW620 cells, and not expressed in LoVo cells (Fig. 1B). Moreover, RIPK1 was expressed at lower levels, suggesting that RIPK-mediated necroptotic signaling was not highly activated in the SW480 cell line (Fig. S2A). Caspase-8 and PANX1 were highly expressed in the HCT116 cell line. Treatment with TNFα significantly decreased cell viability and increased the cleavage of caspase-3 and PANX1 in HCT15 and HCT116 cells (Fig. 1B, C and Table S1). The cleavage of caspase-3 and PANX1 was lower in LoVo, SW480 and SW620 cells (Fig. S2B). These results suggested that the cleavage of PANX1, which promoted ATP release, may be associated with extrinsic apoptosis in the HCT15 and HCT116 cell lines. Phosphorylation of MLKL was observed after TNFα treatment in SW480 and SW620 cells (Fig. S2C), suggesting that MLKL-mediated plasma membrane pore formation to promote ATP release occurred at high levels in the SW480 and SW620 cell lines. Treatment with TNFα, the caspase inhibitor z-DEVD-FMK and the necroptosis inhibitor Nec-1 showed strong induction of caspase-dependent cell death in HCT15 and HCT116 cells (Fig. S2D). These results suggested that TNFα-mediated ATP release might be different in colorectal cancer. However, the sensitivity to TNFα, which promotes ATP release, might be associated with caspase-dependent PANX1 cleavage.

**Fig. 2:**
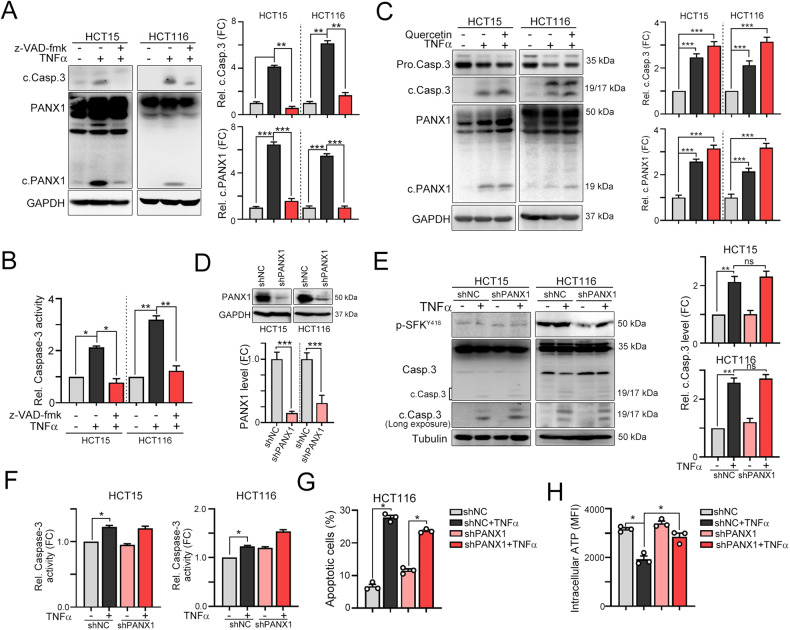
PANX1 cleavage is dispensable for caspase-dependent apoptosis but essential for ATP release. **A** HCT15 and HCT116 cells were treated with TNFα (50 ng/mL) and the caspase inhibitor z-VAD-fmk (10 μM) for 24 h. The cleavage of caspase-3 and PANX1 was evaluated by western blotting (*n* = 3). **p < 0.01 and ***p < 0.001. One-Way ANOVA test. **B** HCT15 and HCT116 cells were treated with TNFα (50 ng/mL) and the caspase inhibitor z-VAD-fmk (10 μM) for 24 h. Caspase-3 activity was evaluated with a caspase-3 activity kit (*n* = 3). *p < 0.05 and **p < 0.01. One-Way ANOVA test. **C** HCT15 and HCT116 cells were treated with TNFα (50 ng/mL) and the SFK inhibitor quercetin (10 μM) for 24 h. The cleavage of caspase-3 and PANX1 was evaluated by western blotting (*n* = 3). ***p < 0.001. One-Way ANOVA test. **D** HCT15 and HCT116 cells were infected with lentivirus carrying shRNA targeting PANX1 and selected with puromycin for 3 days. The knockdown efficacy of lentivirus carrying shRNA targeting PANX1 was evaluated by western blotting (*n* = 3). ***p < 0.001. Unpaired t-test. **E** HCT15^shNC^ and HCT15^shPANX1^ cells were treated with TNFα (50 ng/mL) for 24 h. The cleavage of caspase-3 was evaluated by western blotting (*n* = 3). Similar experiments were carried out in HCT116 cells. **p < 0.01. One-Way ANOVA test. **F** HCT15^shNC^ and HCT15^shPANX1^ cells were treated with TNFα (50 ng/mL) for 24 h. Caspase-3 activity was evaluated with a caspase-3 activity kit (*n* = 3). Similar experiments were carried out in HCT116 cells. *p < 0.05. One-Way ANOVA test. **G** HCT116^shNC^ and HCT116^shPANX1^ cells were treated with TNFα (50 ng/mL) for 24 h. Apoptotic cells were examined by flow cytometry (*n* = 3). *p < 0.05. One-Way ANOVA test. **H** HCT116^shNC^ and HCT116^shPANX1^ cells were treated with TNFα (50 ng/mL) for 6 h. The intracellular ATP content was examined by flow cytometry (*n* = 3). *p < 0.05. One-Way ANOVA test.

The signaling of the proinflammatory cytokine TNFα is mediated by two distinct receptors, TNFR1 and TNFR2, resulting in several opposing cellular functions. TNFR1 mostly promotes cell death, whereas TNFR2 plays a critical role in cell migration and proliferation. To investigate whether TNFα mediates PANX1 cleavage via TNFR1, we generated stable TNFR1- and TNFR2-knockdown HCT15 and HCT116 cell lines with lentivirus carrying shRNA targeting individual genes (Fig. 1D). We found that knockdown of TNFR1 decreased TNFα-mediated caspase-8/caspase-3 cleavage (Fig. 1E). Furthermore, PANX1 cleavage by TNFα was also diminished in TNFR1-knockdown HCT15 and HCT116 cells (Fig. 1E). However, knockdown of TNFR2 enhanced the cleavage of caspase-8, caspase-3 and PANX1 (Fig. 1F). Additionally, neutralizing TNFα signaling by R-7050 (TNFα antagonist) decreased the cleavage of caspase-3 and PANX1 (Fig. 1G), suggesting that PANX1 cleavage in response to TNFα was TNFR1 dependent. Furthermore, inhibition of TNFα-dependent caspase activation significantly reduced the cleavage of PANX1 (Fig. 2A, B). Previous studies also reported that TNFα promotes PANX1 channel opening to release ATP via Src family kinase (SFK)-dependent phosphorylation [18, 36]. To evaluate whether SFK participates in TNFα-dependent ATP release via PANX1 phosphorylation, we combined the SFK inhibitor quercetin with TNFα treatment [37]. Inactivation of SFK by quercetin led to a significant decrease in SFK phosphorylation (Fig. S2E). However, inhibition of SFK did not reduce TNFα-mediated caspase-3 cleavage and partial inhibit ATP release (Fig. 2C and Fig. S2F), suggesting that SFK-mediated PANX1 phosphorylation was not involved in TNFα-mediated ATP release. Furthermore, knockdown of PANX1 did not influence the cleavage of caspase-3 (Fig. 2D–F) or apoptosis (Fig. 2G) but decreased the release of ATP (Fig. 2H), suggesting that PANX1 is dispensable for caspase-dependent apoptosis but influences ATP release in a TNFα-mediated caspase cleavage-dependent manner.

### TNFα enhanced pannexin 1 cleavage in association with chemotherapy-induced cell death to promote ATP release and thus increase cancer immunogenicity

**Fig. 3:**
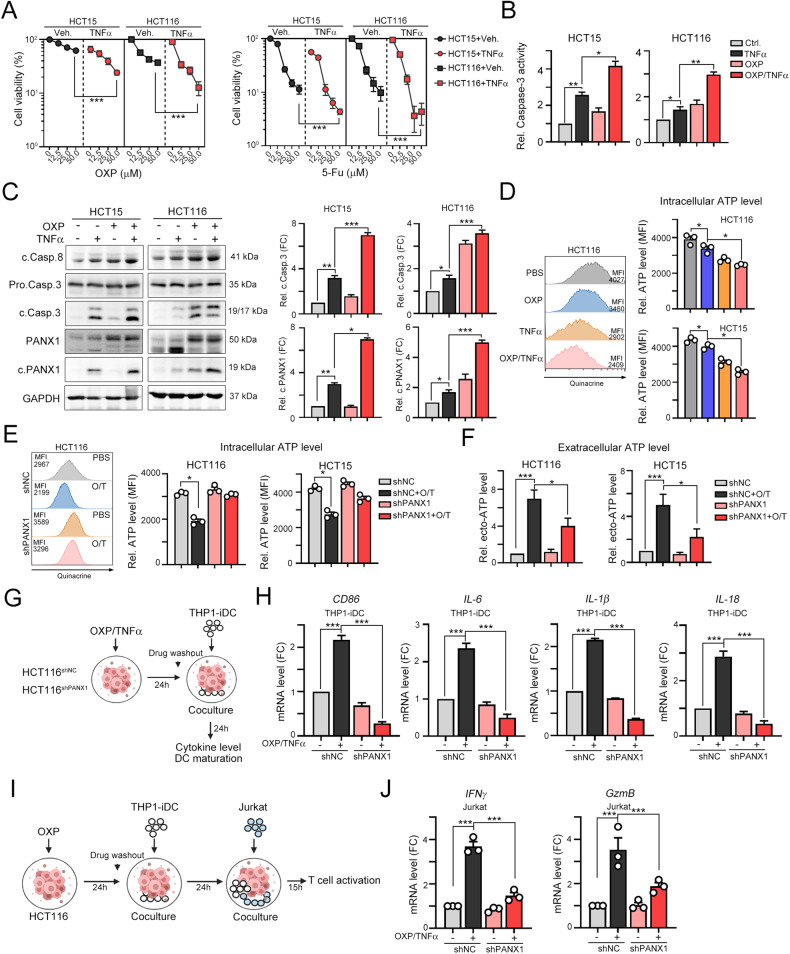
TNFα enhances OXP-induced cell death and ATP release. **A** HCT15 and HCT116 cells were treated with TNFα (50 ng/mL) and OXP or 5-Fu for 24 h. Cell viability was evaluated by CCK assay (*n* = 3). ***p < 0.001. Two-Way ANOVA test. **B** HCT15 and HCT116 cells were treated with TNFα (50 ng/mL) and OXP (25 μM) for 24 h. Caspase-3 activity was evaluated with a caspase-3 activity kit (*n* = 3). *p < 0.05 and **p < 0.01. One-Way ANOVA test. **C** HCT15 and HCT116 cells were treated with TNFα (50 ng/mL) and OXP (25 μM) for 24 h. The cleavage of caspase 8, caspase-3 and PANX1 was evaluated by western blotting (*n* = 3). *p < 0.05, **p < 0.01 and ***p < 0.001. One-Way ANOVA test. **D** After treatment with TNFα and OXP for 6 h, the intracellular ATP content was examined by flow cytometry (*n* = 3). *p < 0.05. One-Way ANOVA test. **E** HCT15^shNC^ and HCT15^shPANX1^ cells were treated with TNFα (50 ng/mL) and OXP (25 μM) for 6 h. The intracellular ATP content was examined by flow cytometry (*n* = 3). *p < 0.05. One-Way ANOVA test. Similar experiments were carried out in HCT116 cells. **F** HCT15^shNC^ and HCT15^shPANX1^ cells were treated with TNFα (50 ng/mL) and OXP (25 μM) for 6 h. The extracellular ATP content was examined with a luminescent ATP detection kit (*n* = 3). *p < 0.05 and ***p < 0.001. One-Way ANOVA test. Similar experiments were carried out in HCT116 cells. **G** Schematic diagram of the DC maturation analysis. HCT116^shNC^ and HCT116^shPANX1^ cells were treated with TNFα (50 ng/mL) and OXP (25 μM) for 24 h. Then, these cells were cocultured with THP1- iDCs for 24 h. **H** The mRNA levels of the DC maturation marker *CD86* and the proinflammatory cytokines *IL-6, IL-1β* and *IL-18* in THP-iDCs that were cocultured with vehicle- or OXP/TNFα-treated HCT116^shNC^ and HCT116^shPANX1^ cells were evaluated by qRT‒PCR (*n* = 3). ***p < 0.001. One-Way ANOVA test. **I** HCT116^shNC^ and HCT116^shPANX1^ cells were treated with TNFα (50 ng/mL) and OXP (25 μM) for 24 h. Then, these cells were cocultured with THP1-iDCs for 24 h and Jurkat T cells for 15 h. **J** The mRNA levels of the cytotoxic cytokines *IFNγ* and *GzmB* in Jurkat T cells that were cocultured with THP-iDCs and vehicle- or OXP/TNFα-treated HCT116^shNC^/HCT116^shPANX1^ cells was evaluated by qRT‒PCR (*n* = 3). ***p < 0.001. One-Way ANOVA test.

The combination of TNFα with DNA-damaging drugs and radiotherapy results in better cell death [24, 38]. Chemotherapeutics, such as oxaliplatin, have been reported to trigger ICD and the release of ATP to promote specific cancer immunogenicity [4, 39, 40]. Therefore, we examined whether TNFα either enhanced the sensitivity of cells to chemotherapeutics or increased cancer immunogenicity to promote antitumor immunity. As shown in Fig. 3A, B, TNFα increased cancer cell sensitivity to the chemotherapeutic drugs oxaliplatin (OXP) and 5-flurouracil (5-Fu). TNFα significantly enhanced the cleavage of caspase-3 and PANX1 after treatment with OXP and 5-Fu (Fig. 3B, C). The release of ATP was markedly increased after exposure to the OXP/TNFα combination treatment (Fig. 3D), indicating that TNFα enhanced OXP-induced ATP release. Furthermore, knockdown of PANX1 led to the maintenance of the level of intracellular ATP (Fig. 3E) and a reduction in the level of extracellular ATP (Fig. 3F), demonstrating that TNFα enhanced OXP-induced ATP release by PANX1.

We further evaluated whether PANX1-mediated ATP release influences cancer immunogenicity to promote DC maturation. Therefore, we treated HCT116^shNC^ and HCT116^shPANX1^ cells with OXP/TNFα and then cocultured them with THP1-differentiated immature DCs for 24 h (Fig. 3G). DC maturation markers *CD86, HLA-A*, *B2M*, and *CD80* and proinflammatory cytokines *IL-6*, *IL-1β*, and *IL-18* were significantly upregulated by OXP/TNFα treatment (Fig. 3H and Fig. S3). Knockdown of PANX1 profoundly reversed the OXP/TNFα-induced increase in the mRNA levels of *CD86*, *HLA-A*, *B2M*, *CD80*, *IL-6*, *IL-1β*, and *IL-18* in THP-differentiated DCs (Fig. 3H and Fig. S3A). Furthermore, we cocultured these primed dendritic cells and Jurkat T cells to evaluate their cytotoxicity (Fig. 3I). Coculture with T cells showed that the cytotoxic ability of HCT116^shPANX1^ cells was markedly decreased along with reduced *IFNγ* and *GzmB* production after treatment with OXP/TNFα (Fig. 3J).

**Fig. 4:**
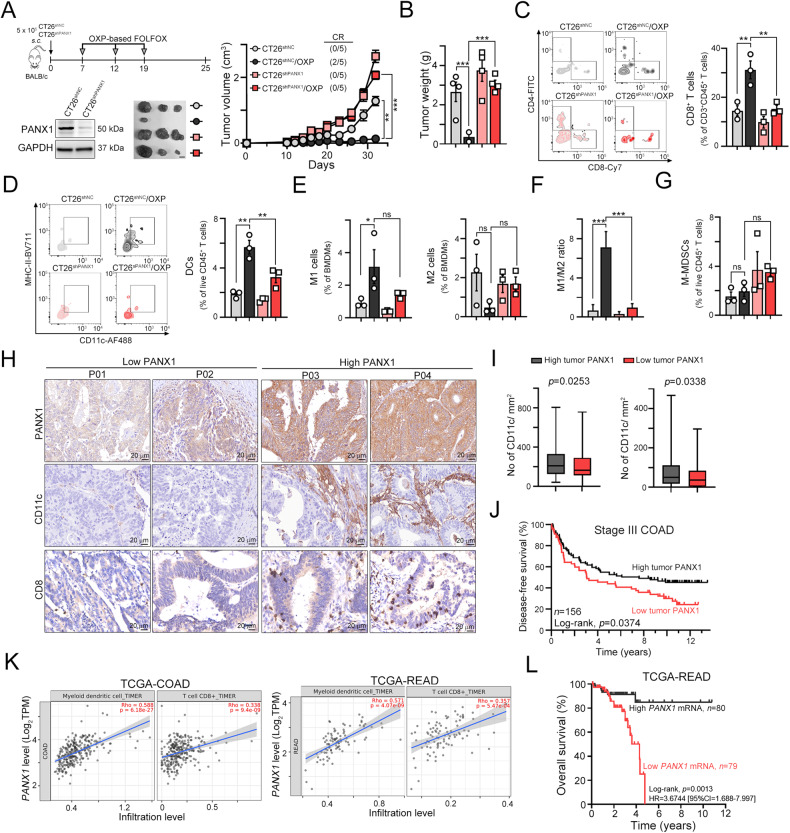
PANX1 deficiency significantly attenuated the cancer immunogenicity that promotes DC maturation and T infiltration in response to chemotherapy in vivo. **A** Schematic diagram of OXP treatment in tumor-bearing BALB/c mice. A total of 5 × 10^5^ CT26^shNC^ and CT26^shPANX1^ cells were subcutaneously injected into the left sides of the backs of BALB/c mice, and the mice were treated with OXP (50 mg/kg) on Days 7, 12, and 19. The tumor tissues were harvested on Day 25 for further analysis. Tumor volume was measured every 3 days (*n* = 5). **p < 0.01 and ***p < 0.001. Two-Way ANOVA test. **B** The resected tumors were weighed on Day 25 (*n* = 5). **p < 0.01 and ***p < 0.001. One-Way ANOVA test. **C** The tumor-infiltrating CD8^+^CD3^+^CD45^+^ T cells within resected tumors were analyzed by flow cytometry (*n* = 3). **p < 0.01. One-Way ANOVA test. **D** The tumor-infiltrating CD11c^+^MHC-II^Hi^CD11b^+^F4/80^-^CD3^-^CD45^+^ live dendritic cells within resected tumors were analyzed by flow cytometry (*n* = 3). **p < 0.01. One-Way ANOVA test. **E** The tumor-infiltrating CD11C^+^CD206^-^CD11b^+^F4/80^+^CD3^-^CD45^+^ (M1-like) and CD11C^-^CD206^+^CD11b^+^F4/80^+^CD3^-^CD45^+^ (M2-like) live macrophages within resected tumors were analyzed by flow cytometry (*n* = 3). *p < 0.05. One-Way ANOVA test. **F** The ratio of M1/M2 within resected tumor tissues. (*n* = 3). ***p < 0.001. One-Way ANOVA test. **G** The tumor-infiltrating Gr-1(Ly6C)^+^CD11c^-^MHC-II^Low^CD11b^+^F4/80^-^CD3^-^CD45^+^ M-MDSCs within resected tumors were analyzed by flow cytometry (*n* = 3). ns: not significant. One-Way ANOVA test. **H** Representative images of tumor PANX1 expression and infiltration of CD11c^+^ DCs and CD8^+^ immune cells within the tumor microenvironment. **I** The tumor expression of PANX1 was positively correlated with the infiltration of CD11c^+^ dendritic cells (*n* = 156, *p* = 0.0253). and CD8^+^ immune cells (*n* = 156, *p* = 0.0338). Unpaired t test. **J** High tumor PANX1 expression was associated with favorable disease-free survival outcomes in stage III colon adenocarcinoma patients (log-rank *p* = 0.0374, *n* = 156). **K** The level of tumor PANX1 was positively correlated with the infiltration of myeloid dendritic cells and CD8^+^ T cells in patients in the TCGA-COAD and TCGA-READ databases, and the data were retrieved from the TIMER2 database (http://timer.cistrome.org). **L** High tumor *PANX1* mRNA expression was associated with favorable disease-free survival outcomes in patients in the TCGA-READ database (log-rank *p* = 0.0013, *n* = 159).

To demonstrate the importance of PANX1 in the response to chemotherapy-induced antitumor immunity in vivo, we subcutaneously injected CT26^shNC^ and CT26^shPANX1^ cells into syngeneic BALB/c mice and treated the mice with OXP (Fig. 4A). We found that knockdown of PANX1 significantly decreased the response to OXP, leading to a larger tumor size and tumor weight compared to those in the CT26^shNC^/OXP group (Fig. 4A, B). We further evaluated the immune cell profiles within resected tumors. The gating strategies were shown in Fig. S4. We found that knockdown of PANX1 alone slightly decreased the number of tumor-infiltrating CD8^+^CD3^+^CD45^+^ T cells and effector/memory CD8^+^ T cells (Fig. 4C and Fig. S4B), and CD11c^+^MHC-II^Hi^CD11b^+^F4/80^-^CD3^-^CD45^+^ dendritic cells (Fig. 4D). However, knockdown of PANX1 significantly decreased tumor-infiltrating CD8^+^T and DCs in response to chemotherapy (Fig. 4A, D). There was no significant difference in the infiltration of M1 (CD11c^+^CD11b^+^F4/80^+^)- and M2 (CD206^+^CD11b^+^F4/80^+^)-like macrophages (Fig. 4F). But the M1/M2 ratio was significantly increased in CT26^shNC^/OXP group, compared to CT26^shPANX1^/OXP group (Fig. 4F). Additionally, the infiltration of F4/80^+^CD11b^+^ macrophages and IFNγ^+^CD8^+^ T cells was also decreased in the CT26^shPANX1^/OXP group compared with the CT26^shNC^/OXP group (Fig. 4E, F). There was no significant difference in the density of Gr1^+^-MDSCs [Gr-1(Ly6C)^+^CD11c^-^MHC-II^Low^CD11b^+^F4/80^-^CD3^-^CD45^+^] (Fig. 4G). Taken together, these results indicated that PANX1-mediated ATP release may enhance cancer immunogenicity and promote chemotherapy-induced antitumor immunity by recruiting DCs and T cells, and repolarization macrophage to eradicate tumor cells.

We then evaluated the clinical relevance of PANX1 in colorectal cancer patients who received adjuvant chemotherapy. We used anti-PANX1 antibodies that recognize the C-terminal intracellular domain of PANX1 proteins (aa 226-426), including cleaved PANX1. We examined the correlation between PANX1 expression and the infiltration of CD11^+^ DCs as well as CD8^+^ T cells within tumor tissues from CRC patients (Fig. 4H). We found that high PANX1 expression in tumor cells was positively correlated with a high density of tumor-infiltrating CD11^+^ DCs and CD8^+^ T cells in patients (Fig. 4I). Moreover, high tumor expression PANX1 was markedly associated with favorable patient survival outcomes (Fig. 4J). Consistent with our observations, high *PANX1* mRNA expression was significantly correlated with high infiltration of myeloid dendritic and CD8^+^ T cells in COAD and READ patients, according to data that were retrieved from the TCGA database (Fig. 4K). High *PANX1* mRNA expression was also associated with favorable survival outcomes in READ patients (Fig. 4L). Similar results were observed in breast cancer (BRCA), lung adenocarcinoma (LUAD), pancreatic adenocarcinoma (PAAD), prostate adenocarcinoma (PRAD) and stomach adenocarcinoma (STAD) according to data from the TCGA database (Fig. S5). Taken together, these results indicated that TNFα-mediated PANX1 cleavage to promote ATP release may enhance cancer immunogenicity and promote chemotherapy-induced antitumor immunity by recruiting DCs and T cells to eradicate tumor cells.

### Blockade of P2RX7 resulted in the recruitment of fewer intratumoral infiltrating T lymphocytes within the TME

**Fig. 5:**
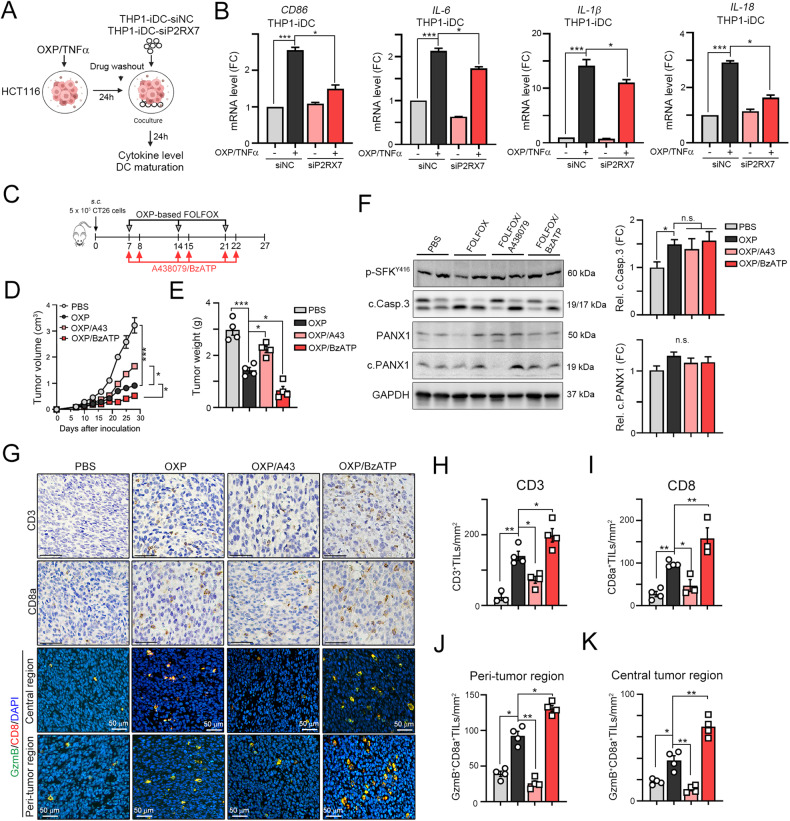
The ATP mimic BzATP significantly increased the therapeutic efficacy of chemotherapy in a colorectal cancer animal model. **A** HCT116 cells were treated with TNFα (50 ng/mL) and OXP (25 μM) for 24 h. Then, these cells were cocultured with THP1-iDCs (THP1-siNC and THP1-siP2RX7) for 24 h. **B** The mRNA levels of the DC maturation marker *CD86* and the proinflammatory cytokines *IL-6, IL-1β* and *IL-18* in THP-iDCs that were cocultured with vehicle- or OXP/TNFα-treated HCT116^shNC^ and HCT116^shPANX1^ cells were evaluated by qRT‒PCR (*n* = 3). ***p < 0.001. One-Way ANOVA test. **C** Schematic diagram of the chemotherapeutic regimen. **D** A total of 5 × 10^5^ CT26 cells were subcutaneously inoculated into the left sides of the backs of BALB/c mice for 7 days, and the mice were administered FOLFOX (20 μM levofolinate, 20 μM 5-Fu, 100 μM OXP), in combination or not with the ATP agonist BzATP (20 μM, i.p. injection) or the ATP antagonist A438079 (20 μM, i.p. injection) on the indicated days. Tumor volume was measured every 3 days. **p < 0.01 and ***p < 0.001. Two-Way ANOVA test. **E** The resected tumors were weighted on Day 26 (mean ± s.e.m., *n* = 5). **p < 0.01 and ***p < 0.001. One-Way ANOVA test. **F** The level of cleaved caspase-3 and PANX1 within resected tumors was analyzed by western blotting (*n* = 3). *p < 0.05 and n.s. = not significant. One-Way ANOVA test. **G** The representative results of CD3^+^, CD8^+^, GzmB^+^CD8^+^ T cells was shown. **H** The quantification of intratumoral CD3 expression is shown (mean ± s.e.m., *n* = 3). *p < 0.05 and **p < 0.01. One-Way ANOVA test. **I** The quantification of intratumoral CD8 expression is shown (mean ± s.e.m., *n* = 3). *p < 0.05 and **p < 0.01. One-Way ANOVA test. **J** The quantification of peri-tumoral GzmB^+^CD8^+^ expression is shown (mean ± s.e.m., *n* = 3). *p < 0.05 and **p < 0.01. One-Way ANOVA test. **K** The quantification of intratumoral GzmB^+^CD8^+^ expression is shown (mean ± s.e.m., *n* = 3). *p < 0.05 and **p < 0.01. One-Way ANOVA test.

Additionally, we evaluated whether deficiency in the ATP receptor P2RX7 may attenuate dendritic cell maturation (Fig. 5A). Indeed, we found that lower mRNA levels of *CD86, HLA-A*, *B2M*, *CD80*, and the proinflammatory cytokines *IL6*, *IL-1β*, and *IL18* were induced by OXP/TNFα, suggesting that PANX1-mediated ATP release promoted dendritic cell maturation via P2RX7 (Fig. 5B and S6) and that the PANX1-P2RX7 axis, which promotes ATP release, regulates OXP-induced cancer immunogenicity. To verify that ATP release, which promotes P2RX7-mediated dendritic cell maturation, can enhance the therapeutic efficacy of chemotherapy by eliciting antitumor immunity, we subcutaneously inoculated BALB/c mice with syngeneic CT26 colon carcinoma cells. The BALB/c mice received FOLFOX chemotherapy (folinic acid, 5-fluorouracil, and oxaliplatin), which is the standard adjuvant chemotherapy for treating CRC after surgical resection [41]. Moreover, we treated the mice with the combination of the benzoyl ester of ATP, BzATP, which is the most potent P2X7 agonist, and the small molecule A438079, which is a competitive P2X7 receptor antagonist (Fig. 5C). After treatment, we found that administration of the P2RX7 antagonist A438079 significantly influenced FOLFOX-induced tumor regression (5D and 5E). BzATP enhanced FOLFOX-induced tumor regression and decreased tumor weight (Fig. 5D, E). We found that there was no significant difference in the cleavage of caspase-3 and PANX1 (Fig. 5F), suggesting that BzATP might participate in P2RX7-mediated DC maturation to promote antitumor immunity.

**Fig. 6:**
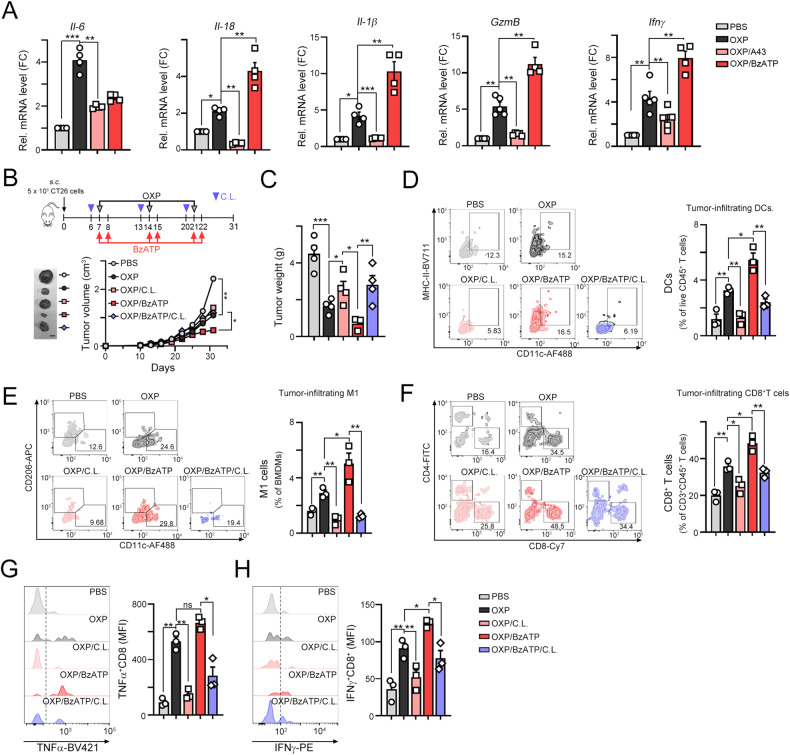
The ATP mimic BzATP significantly increased the infiltration of immune cells in combination with chemotherapy. **A** The mRNA levels of the proinflammatory cytokines *Il-6, Il-18, Il-1β, Gzmb,* and *Ifnγ* within tumors were evaluated by qRT‒PCR (*n* = 3). **p < 0.01 and ***p < 0.001. One-Way ANOVA test. **B** Schematic diagram of the chemotherapeutic regimen. A total of 5 × 10^5^ CT26 cells were subcutaneously injected into the left sides of the backs of BALB/c mice, and 7 days later, the mice were treated with OXP (50 mg/kg), the ATP agonist BzATP (20 μM, i.p. injection) or clodronate liposomes (50 μL/mouse) on the indicated days (*n* = 5). The tumor tissues were harvested on Day 31 for further analysis. Tumor volume was measured every 3 days (*n* = 5). *p < 0.05 and **p < 0.01. Two-Way ANOVA test. **C** The resected tumors were weighed on Day 31 (*n* = 3). *p < 0.05, **p < 0.01 and ***p < 0.001. One-Way ANOVA test. **D** The tumor-infiltrating CD11c^+^MHCII^Hi^ dendritic cells within resected tumors were analyzed by flow cytometry (*n* = 3). *p < 0.05 and **p < 0.01. One-Way ANOVA test. **E** The tumor-infiltrating CD11c^+^F4/80^+^CD11b^+^ M1-macrophages within resected tumors were analyzed by flow cytometry (*n* = 3). *p < 0.05 and **p < 0.01. One-Way ANOVA test. **F** The tumor-infiltrating CD8^+^CD3^+^ T cells within resected tumors were analyzed by flow cytometry (*n* = 3). *p < 0.05 and **p < 0.01. One-Way ANOVA test. **G** The tumor-infiltrating TNFα^+^CD8^+^CD3^+^ T cells within resected tumors were analyzed by flow cytometry (*n* = 3). *p < 0.05 and **p < 0.01. One-Way ANOVA test. **H** The tumor-infiltrating IFNγ^+^CD8^+^CD3^+^ T cells within resected tumors were analyzed by flow cytometry (*n* = 3). *p < 0.05 and **p < 0.01. One-Way ANOVA test.

To explore the mechanisms by which P2RX7 blockade affects tumor growth *via* antitumor immunity, we evaluated the infiltration of general CD3^+^ and cytotoxic CD8^+^ T lymphocytes. FOLFOX treatment recruited CD3^+^ and CD8^+^ immune cells into the TME (Fig. 5G, H, I). When FOLFOX was combined with BzATP, the density of CD3^+^ and CD8^+^ immune cells was markedly elevated (Fig. 5H, I). Moreover, the density of granzyme B (GzmB)^+^ CD8^+^ immune cells in the central region was profoundly increased in the FOLFOX/BzATP group (Fig. 5J, K). The mRNA levels of the proinflammatory factors *Il-6, Il-1β, Il-18*, *GzmB* and *Ifnγ* were elevated in the FOLFOX/BzATP group (Fig. 6A), suggesting that immunomodulatory ATP significantly enhanced dendritic cell maturation, promoting the release of proinflammatory cytokines and the recruitment of tumor-infiltrating CD8^+^ T cells.

To further demonstrate the immunomodulatory effect of ATP on the therapeutic efficacy of OXP via dendritic cells, we depleted antigen presenting cells (APCs), including DCs, with low dose of clodronate-liposome (C.L.) [42, 43] prior to OXP and ATP agonist treatment (Fig. 6B and Fig. S7). We found that OXP led to a significant decrease in tumor growth when administered in combination with the P2RX7 activator BzATP. However, this therapeutic efficacy was attenuated by C.L. administration, leading to a larger tumor size and tumor weight (Fig. 6B, C). By analyzing the immune cell profiles in the resected tumors, we found that tumor-infiltrating DCs (CD11c^+^MHC-II^Hi^CD3^-^CD45^+^) and M1 macrophages (CD11c^+^CD11b^+^F4/80^+^) were significantly depleted by clodronate-liposome and even the administration of OXP and BzATP (Fig. 6D, E and Fig. S7A). There was no significant difference in the density of Gr1^+^CD11b^+^ MDSCs (Fig. S7B). Additionally, the infiltration of CD8^+^ T cells (CD8^+^CD3^+^CD45^+^) and effector/memory CD8^+^ T_EM_ cells (CD44^+^CD62L^-^CD8^+^CD3^+^CD45^+^) T was also decreased in OXP/BzATP/CL group, compared to OXP/BzATP group (Fig. 6F, Fig. S7C and Fig. S7D). Furthermore, the cytotoxic TNFα^+^CD8^+^ and IFNγ^+^CD8^+^ T cells was decreased (Fig. S7E, Fig. 6G, H) in OXP/BzATP/CL group, compared to OXP/BzATP group. Taken together, these results indicated that the immunomodulatory effect of ATP promoted chemotherapy-induced antitumor immunity via a mechanism that relied on the recruitment of DCs via P2RX7.

### High PANX1 expression and wild-type P2RX7-E496A were associated with favorable survival outcomes in stage III CRC patients

**Fig. 7:**
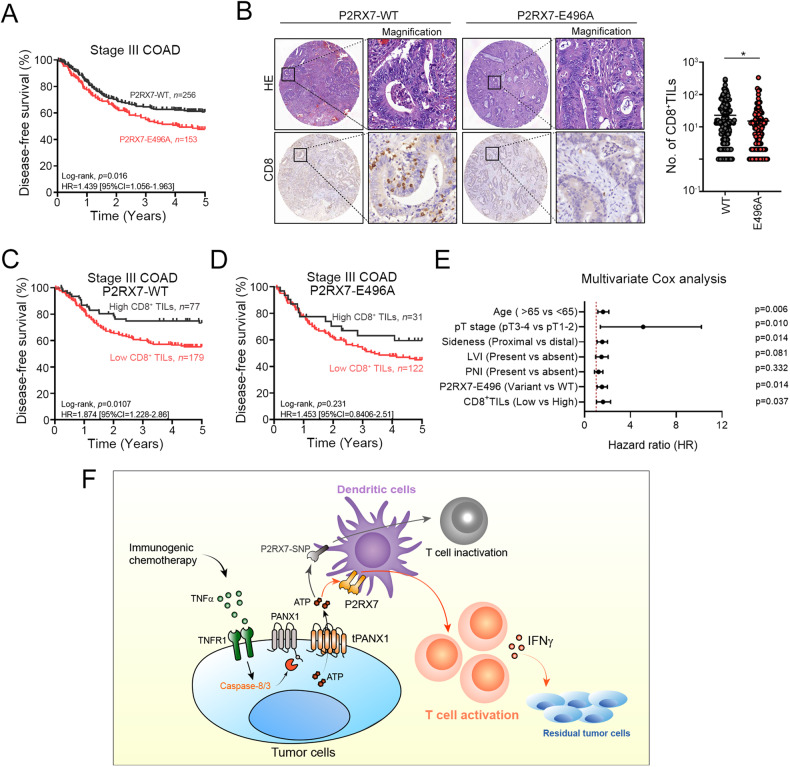
High tumor PANX1 expression and wild-type P2RX7 expression were associated with high infiltration of cytotoxic T lymphocytes and favorable survival outcomes in CRC patients who received adjuvant chemotherapy. **A** The P2RX7-E496A allele was associated with survival outcomes in stage III COAD patients who received adjuvant chemotherapy (*n* = 409, log-rank *p* = 0.016). **B** P2RX7 variant expression was significantly associated with the infiltration of cytotoxic CD8^+^ TILs (unpaired t test, *p* = 0.04). **C** Patients expressing wild-type P2RX7 and with high infiltration of CD8^+^ immune cells had favorable survival outcomes (*n* = 256, log-rank, *p* = 0.0107). **D** Mutant P2RX7 expression and high infiltration of CD8^+^ immune cells were not associated with survival outcome (*n* = 231, log-rank, *p* = 0.231). **E** Multivariate Cox analysis showed that P2RX7 is an independent prognostic factor for stage III COAD patients who received adjuvant chemotherapy (HR = 1.47, 95% CI = 1.08–1.99, *p* = 0.014). **F** The proposed mechanism by which TNFα-mediated PANX1 cleavage promotes ATP release to trigger P2RX7-dependent dendritic cell activation and enhance antitumor immunity following immunogenic chemotherapy in colorectal cancer.

We then examined the effect of the loss-of-function P2RX7 polymorphism, P2RX7-E496A (rs3751143), on 5-year disease-free survival (DFS) in stage III colon carcinoma patients who were treated with adjuvant chemotherapy after surgery (Table S2). At the 5-year follow-up, 149 patients (149/410 = 36.3%) had died. The 5-year DFS and OS rates were 53.5% and 63.7%, respectively. Patients with young age, pathological T1-2 stage, tumor location in the distant colon, absence of lymphovascular invasion (LVI), and perineural invasion (PNI) had a better 5-year DFS (Table S3). Moreover, compared to patients carrying the P2RX7-E496A polymorphism, patients expressing wild-type P2RX7 had better 5-year DFS among stage III CRC patients who received adjuvant chemotherapy (60.9% vs. 51.0%, *p* = 0.016, Fig. 7A). There was no significant difference with stage II CRC patients (Fig. S8). These results suggested that the status of P2RX7 was associated with tumor relapse after chemotherapy treatment. Moreover, we found that patients expressing the wild-type P2RX7 allele had a high density of cytotoxic CD8^+^ T cells within the TME (Fig. 7B and Table S4). However, patients carrying the P2RX7-E496A variant had fewer CD8^+^ TILs, suggesting that the P2RX7 polymorphism was inversely correlated with the infiltration of T lymphocytes within the TME (Fig. 7B). Kaplan-Meier survival analysis indicated that patients who both expressed wild-type P2RX7 and exhibited CD8^+^ TIL infiltration had a better DFS (Fig. 7C, D). Multivariate Cox analysis also showed that P2RX7 is an independent prognostic factor for stage III COAD patients who received adjuvant chemotherapy (HR = 1.47, 95% CI = 1.08–1.99, *p* = 0.014, Fig. 7E, Table S5). Taken together, these results showed that the PANX1-P2RX7 axis was critical for chemotherapy-induced antitumor immunity. Low PANX1 expression or loss of PANX7 function may increase the risk of tumor relapse by reducing the recruitment of T lymphocytes that are necessary for antitumor immunity.

## Discussion

The pathways that initiate and execute immunogenic cell death are complex. Here, we show that TNFα sensitizes colorectal cancer promote the release of ATP by caspase-8/3-mediated PANX1 cleavage via TNFR1 during chemotherapy-induced cell death. TNFα significantly increases the cell response to chemotherapy as well as the release of ATP to enhance cancer immunogenicity via the P2RX7 receptor, thus promoting DC maturation and proinflammatory cytokine production and leading to T-cell-mediated cytotoxicity. Activation of P2RX7 markedly enhanced the therapeutic efficacy of chemotherapy by increasing T-cell infiltration to promote antitumor immunity in vivo. Moreover, patients with lower tumor PANX1 expression or patients who carried the loss-of-function P2RX7 variant have less immune cell infiltration and poor survival outcomes after adjuvant chemotherapy treatment. Taken together, these results showed that TNFα administration enhances cancer immunogenicity *via* caspase-dependent PANX1 cleavage that mediates ATP release.

TNFα is produced by both immune cells and tumor cells and in the tumor microenvironment, TNFα has the potential to activate cell death pathways in colon cancer cells [24, 33, 44, 45]. TNFα studies have highlighted its potential for use as a highly specific anticancer drug in the treatment of a wide variety of tumors, especially colorectal and lung cancers. However, the clinical benefit of single-agent recombinant TNFα was not realized in clinical trials due to systemic toxicity [46], limiting its clinical application. However, recent studies showed that TNFα-mediated cell death plays an indispensable role in immune surveillance by recruiting cytotoxic T lymphocytes [26, 27]. Loss of TNFα sensitivity leads to tumor immune evasion independent of perforin-mediated killing, suggesting that resensitizing cancer cells to TNFα-mediated killing might be a potent mechanism for killing antigen-negative tumor cells [26, 27]. Furthermore, intratumoral administration of an oncolytic virus encoding TNFα and IL-2 also enhanced anti-PD-1 efficacy in mouse melanoma by improving CD8^+^ T-cell infiltration [47], suggesting that delivering high concentrations of TNFα to tumors can enhance the efficacy of immunotherapy. These results are consistent with another work in which targeting TNFα to the tumor vasculature also increased the therapeutic efficacy of immune checkpoint blockade as well as adoptive cell therapy by enhancing T-cell infiltration [48]. Antibody-based delivery of TNFα to the tumor also increased the therapeutic efficacy of cancer vaccines by promoting T-cell infiltration [49]. These results suggested that TNFα-induced cell death should promote immune activation by multiple mechanisms, such as increasing the delivery of antigens or enhancing the release of “danger signals”. In our studies, we found that proinflammatory TNFα may execute PANX1 cleavage via the TNFR1-caspase-8/3-mediated apoptotic pathway to promote ATP release, which is triggered by chemotherapeutic drugs [19]. Knockdown of PANX1 significantly reduced the release of ATP and decreased the cancer immunogenicity that promotes dendritic cell maturation and proinflammatory cytokine production, resulting in decreased T-cell-mediated cytotoxicity. Several immunogenic chemotherapeutic agents, such as oxaliplatin and doxorubicin, have been reported to elicit ICD by increasing the release of DAMPs, including ATP, HMGB1, HSP70 and CARL. Here, we found that the addition of TNFα increased the immunogenicity of OXP-treated cancer cells via the PANX1-ATP-P2RX7 axis. Low expression of PANX1 or a loss-of-function P2RX7 allele was associated with reduced infiltration of cytotoxic T lymphocytes, suggesting that the PANX1-induced secretion of ATP to promote P2RX7 signaling is critical for a favorable response to chemotherapy [7–9, 50]. Additionally, depletion with antigen presenting cells significantly decreased the therapeutic efficacy of ATP mimetic in combination with chemotherapy, suggesting that ATP enhanced DCs maturation and T-cell infiltration to promote OXP-induced antitumor immunity. However, we cannot exclude the influences of tumor-associated macrophage depletion by liposomal clodronate [42, 43]. Therefore, we need to further investigate in the future.

TNFα executes cell death by either an apoptotic or necroptotic process. If the tumor is intrinsically TNFα-sensitive, TNFα will initiate apoptosis and promote PANX1 cleavage [51–53]. However, recent studies have shown that activation of the TNFα-dependent necroptotic protein receptor-interacting serine/threonine protein kinase 1 (RIPK1) can drive antigen cross priming of CD8^+^ T cells [54, 55]. RIPK1-mediated immunogenic cell death and cytokine release promote antitumor immunity [3, 34]. Our results showed that RIPK1-dependent MLKL phosphorylation was not observed in TNFα-sensitive colorectal cancer cells. Inhibition of RIPK1 by Nec-1 did not influence the sensitivity of HCT15 and HCT116 cells to TNFα, suggesting that TNFα may utilize two distinct molecular mechanisms to enhance antitumor immunity. Supporting our findings, Douanne et al. reported that PANX1 limits the production of proinflammatory cytokines during necroptosis [56], suggesting that PANX1 is required for ATP release during TNFα-dependent apoptosis but not for TNFα-mediated necroptosis and inflammasome activation [57, 58].

The P2RX7 receptor is a well-known ATP-gated ion channel that is associated with the activation of multiple inflammatory pathways, including the NLRP3 inflammasome, and the release of cytokines such as IL-1β and IL-18 from innate immune cells [59, 60]. Therefore, P2RX7 acts as a DAMP receptor to sensitize cells to ATP and trigger NLRP3 inflammation. Breast cancer patients carrying loss-of-function alleles of *P2RX7*-E496A (rs3751143) exhibit reduced therapeutic efficacy of adjuvant chemotherapy [21]. P2RX7-E496A limits the affinity of P2RX7 for ATP and causes resistance against ATP-induced cell death in *Mycobacterium*-infected macrophages, confirming the functional effect of this polymorphism on the immune response [22]. Moreover, patients who are homozygous or heterozygous carriers of *P2RX7-E496A* (rs3751143) experienced more rapid relapse than patients who carry the wild-type allele [21].

Taken together, our results indicated that chemotherapy sensitizes colorectal cancer to release ATP via the caspase-8/3-mediated cleavage of PANX1 downstream of TNFα. TNFα significantly enhances the response to chemotherapy as well as the release of ATP to enhance cancer immunogenicity via the P2RX7 receptor, promoting DC maturation and proinflammatory cytokine production and leading to T-cell-mediated cytotoxicity. Administration of an ATP mimic markedly enhances the therapeutic efficacy of chemotherapy by increasing T-cell infiltration to promote antitumor immunity in vivo. Low tumor PANX1 expression or the loss-of-function P2RX7 allele leads to reduced infiltration of immune cells and poor survival outcomes after adjuvant chemotherapy treatment in CRC patients. These results indicated that PANX1-ATP-P2RX7 signaling is essential for TNFα-dependent cancer immunotherapy in CRC patients.

## Data sharing

The original dataset is available on request from the corresponding author.

### Supplementary information

Confirm mails and pre-acceptance request form

supplementary information

Supplemental Material-Raw data
