# Supplementary material for: TNFα modulates PANX1 activation to promote ATP release and enhance P2RX7-mediated antitumor immune responses after chemotherapy in colorectal cancer
Source: Cell Death Dis. 2024 Jan 9;15(1):24. doi: 10.1038/s41419-023-06408-5 (PMC10776587; doi:10.1038/s41419-023-06408-5)
Supplement: Supplementary file 1 — Confirm mails and pre-acceptance request form [file 41419_2023_6408_MOESM1_ESM.pdf]

SPRINGER NATURE

Change of authorship request form - Journals (pre-acceptance)

Important information. Please read.

- This form should be used by authors to request any change in authorship (adding/deleting authors) including changes in corresponding authors. This form should not be used for name changes. Please fully complete all sections. Use black ink and block capitals and provide each author's full name with the given name first followed by the family name.

- By signing this declaration, all authors guarantee that the order of the authors are in accordance with their scientific contribution, if applicable as different conventions apply per discipline, and that only authors have been added who made a meaningful contribution to the work.

- Please note, in author collaborations where there is formal agreement for representing the collaboration, it is sufficient for the representative or legal guarantor (usually the corresponding author) to complete and sign the Authorship Change Form on behalf of all authors, **next to the added/removed author(s)**. **(Complete Section 3, followed by Section 6.)**
  In author collaborations where there is no formal agreement for representing the collaboration and **there are more than 10 authors**, one may sign for all, provided the signer appends correspondence that attests that each of the authors have agreed to the change **and the added/removed authors sign the form**. **(Complete Section 3, followed by Section 6.)**

- Please note, we cannot investigate or mediate any authorship disputes. If you are unable to obtain agreement from all authors (including those who you wish to be removed) you must refer the matter to your institution(s) for investigation. Please inform us if you need to do this.

- If you are not able to return a fully completed form within **30 days** of the date that it was sent to the author requesting the change, we may have to withdraw your manuscript. We cannot publish manuscripts where authorship has not been agreed by all authors (including those who have been removed).

- Incomplete forms will be rejected.

- Please return/upload this form, fully completed, to the Journals Editorial Office. The Journal and/or Publisher will consider the information you have provided to decide whether to approve the proposed change in authorship. We may decide to contact your institution for more information or undertake a further investigation, if appropriate, before making a final decision.

SPRINGER NATURE Change of authorship request form - Journals (pre-acceptance)

Section 1: Please provide the current title of manuscript

Manuscript ID no.: CDDIS-23-1970RRR

Title: TNF-alpha modulates PANX1 activation to promote ATP release and enhance P2RX7-mediated antitumor immune responses after chemotherapy in colorectal cancer

Section 2: Please provide the previous authorship, in the order shown on the manuscript before the changes were introduced. Please indicate the corresponding author by adding (CA) behind the name.

	First name(s)	Family name	ORCID or SCOPUS id, if available
1st author	Kevin Chih-Yang	Huang	
2nd author	Shu-Fen	Chiang	
3rd author	Pei-Chun	Lin	
4th author	Wei-Ze	Hong	
5th author	Pei-Chen	Yang	
6th author	Tsung-Wei	Chen	
7th author	Tao-Wei	Ke	
8th author	Ji-An	Liang	
9th author	William Tzu-Liang	Chen	
10th author	KS Clifford	Chao	

Please use an additional sheet if there are more than 10 authors.

SPRINGER NATURE Springer Nature is one of the world's leading global research, educational and professional publishers, created in May 2015 through the combination of Nature Publishing Group, Palgrave Macmillan, Macmillan Education and Springer Science+Business Media.

SPRINGER NATURE # Change of authorship request form - Journals (pre-acceptance)

Section 3: Please provide a justification for change. Please use this section to explain your reasons for changing the authorship of your manuscript, e.g. what necessitated the change in authorship? Please refer to the (journal) policy pages for more information about authorship. Please explain why omitted authors were not originally included and/or why authors were removed on the submitted manuscript.

In response to the reviewers' comments in first round (CDDIS-23-1970R), we added immunoblotting, qRT-PCR, coculture and two animal experiments to demonstrate the changes in immune cells (flow cytometry).
For these experiments and data analysis, we ask Hui-Ping Chang and Shin-Lei Peng to carry out.
Regarding to their contributions in the revised manuscript, we add Hui-Ping Chang and Shin-Lei Peng as the co-author.
All authors agree and confirm her contribution and authorship. We have described their contributions in the authors' contribution statement

Section 4: Proposed new authorship. Please provide your new authorship list in the order you would like it to appear on the manuscript. Please indicate the corresponding author by adding (CA) behind the name. <u>If the Corresponding Author has changed, please indicate the reason under section 3</u>.

	First name(s)	Family name (this name will appear in full on the final publication and will be searchable in various abstract and indexing databases)	Affiliated institute	E-mail address
1st author	Kevin Chih-Yang	Huang	China Medical University Hospital	flylerd0425@gmail.com
2nd author	Shu-Fen	Chiang	Feng-Yuan Hospital	suvenchiang@gmail.com
3rd author	Pei-Chun	Lin	China Medical University	catlovefish0907@gmail.com
4th author	Wei-Ze	Hong	China Medical University	b23750046@gmail.com
5th author	Pei-Chen	Yang	China Medical University	u9810029@cmu.edu.tw
6th author	Hui-Ping	Chang	China Medical University	winter12snow84@gmail.com
7th author	Shin-Lei	Peng	China Medical University	speng@mail.cmu.edu.tw
8th author	Tsung-Wei	Chen	China Medical University	runrunrungo@yahoo.com.tw
9th author	Tao-Wei	Ke	China Medical University	d18047@mail.cmuh.org.tw
10th author	Ji-An	Liang	China Medical University	D4615@mail.cmuh.org.tw
11th author	William Tzu-Liang	Chen	China Medical University	wtchen@mail.cmuh.org.tw
12th author	KS Clifford	Chao	China Medical University	d94032@mail.cmuh.org.tw

Please use an additional sheet if there are more than 10 authors.

SPRINGER NATURE Springer Nature is one of the world's leading global research, educational and professional publishers, created in May 2015 through the combination of Nature Publishing Group, Palgrave Macmillan, Macmillan Education and Springer Science+Business Media.

SPRINGER NATURE # Change of authorship request form - Journals (pre-acceptance)

Section 5: Author contribution, Acknowledgement and Disclosures. Please use this section to provide a new disclosure statement and, if appropriate, acknowledge any contributors who have been removed as authors and ensure you state what contribution any new authors made (if applicable per the journal or book (series) policy). Please ensure these are updated in your manuscript - after approval of the change(s) - as our production department will not transfer the information in this form to your manuscript.

New acknowledgements:
New Disclosures (financial and non-financial interests, funding):
New Author Contributions statement (if applicable per the journal policy):

State 'Not applicable' if there are no new authors.

SPRINGER NATURE Springer Nature is one of the world's leading global research, educational and professional publishers, created in May 2015 through the combination of Nature Publishing Group, Palgrave Macmillan, Macmillan Education and Springer Science+Business Media.

SPRINGER NATURE

Change of authorship request form - Journals (pre-acceptance)

Section 6: Declaration of agreement. *All* authors, unchanged, new and removed *must* sign this declaration.
(NB: Please print the form, (docu)-sign and return/upload a scanned copy. Please note that signatures that have been inserted as an image file are acceptable as long as it is handwritten. Typed names in the signature box are unacceptable.) *** Please delete as appropriate. Delete all of the bold if you were on the original authorship list and are remaining as an author.**

	First name	Family name		Signature	Date
1st author	Kevin Chih-Yang	Huang	I agree to the proposed new authorship shown in section 4 **/and the addition/removal*of my name to the authorship list** /and the proposed change in corresponding author	Kevin Chih-Yang Huang	2023.12.15
2nd author	Shu-Fen	Chiang	I agree to the proposed new authorship shown in section 4 **/and the addition/removal*of my name to the authorship list** /and the proposed change in corresponding author	Shu-Fen Chiang	2023,12,15
3rd author	Pei-Chun	Lin	I agree to the proposed new authorship shown in section 4 **/and the addition/removal*of my name to the authorship list** /and the proposed change in corresponding author	Lin, Pei-Chun	2023,12,15
4th authors	Wei-Ze	Hong	I agree to the proposed new authorship shown in section 4 **/and the addition/removal*of my name to the authorship list** /and the proposed change in corresponding author	HONG, WEI-ZE	2023.12.15
5th author	Pei-Chen	Yang	I agree to the proposed new authorship shown in section 4 **/and the addition/removal*of my name to the authorship list** /and the proposed change in corresponding author	Pei-Chen Yang	2023.12.15
6th author	Hui-Ping	Chang	I agree to the proposed new authorship shown in section 4 **/and the addition/removal*of my name to the authorship list** /and the proposed change in corresponding author	Chang, Hui-Ping	2023.12.15
7th author	Shin-Lei	Peng	I agree to the proposed new authorship shown in section 4 **/and the addition/removal*of my name to the authorship list** /and the proposed change in corresponding author	Shin-Lei Peng	2023,12,15

SPRINGER NATURE Springer Nature is one of the world's leading global research, educational and professional publishers, created in May 2015 through the combination of Nature Publishing Group, Palgrave Macmillan, Macmillan Education and Springer Science+Business Media.

SPRINGER NATURE

Change of authorship request form - Journals (pre-acceptance)

Section 6: Declaration of agreement. ***All*** **authors, unchanged, new and removed** ***must*** **sign this declaration.**
(NB: Please print the form, (docu)-sign and return/upload a scanned copy. Please note that signatures that have been inserted as an image file are acceptable as long as it is handwritten. Typed names in the signature box are unacceptable.) *** Please delete as appropriate. Delete all of the bold if you were on the original authorship list and are remaining as an author.**

	First name	Family name		Signature	Date
8th author	Tsung-Wei	Chen	I agree to the proposed new authorship shown in section 4 **/and the addition/removal*of my name to the authorship list** /and the proposed change in corresponding author	Chen, Tsung-Wei	2023.12.15
9th author	Tao-Wei	Ke	I agree to the proposed new authorship shown in section 4 **/and the addition/removal*of my name to the authorship list** /and the proposed change in corresponding author	Tao-Wei Ke	2023.12.15
10th author	Ji-An	Liang	I agree to the proposed new authorship shown in section 4 **/and the addition/removal*of my name to the authorship list** /and the proposed change in corresponding author	Ji-An Liang	2023.12.15
11th authors	William Tzu-Liang	Chen	I agree to the proposed new authorship shown in section 4 **/and the addition/removal*of my name to the authorship list** /and the proposed change in corresponding author	William Tzu-Liang Chen	2023.12.15
12th author	KS Clifford	Chao	I agree to the proposed new authorship shown in section 4 **/and the addition/removal*of my name to the authorship list** /and the proposed change in corresponding author	[illegible]	2023.12.18
			I agree to the proposed new authorship shown in section 4 **/and the addition/removal*of my name to the authorship list** /and the proposed change in corresponding author		
			I agree to the proposed new authorship shown in section 4 **/and the addition/removal*of my name to the authorship list** /and the proposed change in corresponding author		

SPRINGER NATURE Springer Nature is one of the world's leading global research, educational and professional publishers, created in May 2015 through the combination of Nature Publishing Group, Palgrave Macmillan, Macmillan Education and Springer Science+Business Media.

	First name	Family name		Signature	Date
8th author			I agree to the proposed new authorship shown in section 4 /**and the addition/removal*of my name to the authorship list** /and the proposed change in corresponding author		
9th author			I agree to the proposed new authorship shown in section 4 /**and the addition/removal*of my name to the authorship list** /and the proposed change in corresponding author		
10th author			I agree to the proposed new authorship shown in section 4 /**and the addition/removal*of my name to the authorship list** /and the proposed change in corresponding author		

Please use an additional sheet if there are more than 10 authors.

In case of author collaborations with formal agreement:

	Name of consortium/consortia	First name	Family name		Signature	Date
Representative/legal guarantor				I agree to the proposed new authorship shown in section 4 /**and the addition/removal*of my name to the authorship list** /and the proposed change in corresponding author		

Both added/removed authors should complete the information in the first table under Section 6.

---- End of form ----

SPRINGER NATURE Springer Nature is one of the world's leading global research, educational and professional publishers, created in May 2015 through the combination of Nature Publishing Group, Palgrave Macmillan, Macmillan Education and Springer Science+Business Media.

Gmail 黃智洋 <flylerd0425@gmail.com>

Decision letter for CDDIS-23-1970RR: Accepted in Principle

14 封郵件

cddisease@springernature.com <cddisease@springernature.com> 2023年12月5日 凌晨12:19
回覆: cddisease@springernature.com
收件者: flylerd0425@gmail.com

Manuscript Number: CDDIS-23-1970RR
Title: TNFa modulates PANX1 activation to promote ATP release and enhance P2RX7-mediated antitumor immune responses after chemotherapy in colorectal cancer
Authors: Kevin Chih-Yang Huang, Shu-Fen Chiang, Pei-Chun Lin, Wei-Ze Hong, Pei-Chen Yang, Tsung-Wei Chen, Tao-Wei Ke, Ji-An Liang, William Tzu-Liang Chen, K. S. Clifford Chao, Hui-Ping Chang, and Shin-Lei Peng

Dear Professor Huang,

I am very pleased to inform you that your manuscript CDDIS-23-1970RR entitled "TNFa modulates PANX1 activation to promote ATP release and enhance P2RX7-mediated antitumor immune responses after chemotherapy in colorectal cancer" has been **provisionally accepted** for publication in *Cell Death & Disease*.

PLEASE READ THE FOLLOWING CAREFULLY

Please note that we won't be able to officially confirm acceptance of your manuscript and proceed with the publication process until the final version of your manuscript has been submitted as per the instruction below.

Cell Death & Disease is a fully open access journal. Articles are made freely accessible on publication under a http://creativecommons.org/licenses/by/4.0"" target=""_blank""> CC BY license (Creative Commons Attribution 4.0 International License). This license allows maximum dissemination and re-use of open access materials and is preferred by many research funding bodies. For further information about article processing charges, open access funding, and advice and support from Nature Research, please visit https://www.nature.com/cddis/open-access

At acceptance, you will be provided with instructions for completing this CC BY license on behalf of all authors. This grants us the necessary permissions to publish your paper. Additionally, you will be asked to declare that all required third party permissions have been obtained, and to provide billing information in order to pay the article-processing charge (APC).

For more detailed information please check the https://www.nature.com/cddis/authors-and-referees/gta"> Guide to the Authors.

1. SUBMITTING YOUR FINAL FILES:

Please submit your final manuscript here:
https://mts-cddis.nature.com/cgi-bin/main.plex?el=A3CA1bdn1C6WCL2I3A9ftdPG2q2tmbk6YAqQkefON1LAZ

- Files must not contain highlighting or tracked changes.
- Cell Death & Disease references should follow the Vancouver format. All authors should be listed for papers with up to six authors; for papers with more than six authors, the first six only should be listed, followed by et al.
- Figure and table legends must be included at the end of article file, after the references.
- Figure files must be uploaded as individual files and then included in the merged file, ensuring that figures with multiple panels are uploaded as one file (e.g. Fig. 1A, 1B uploaded as Fig. 1).
- Tables must be editable and either uploaded as separate files or included in the article file, after the references.
- All supplementary information, including supplementary figure and table legends, must be uploaded separately and not included in the article file.
- An Author Contribution Statement must be provided.
- All authors identified as 'corresponding author' should create and link their Open Researcher and Contributor Identifier (ORCID) with their account on the Manuscript Tracking System (MTS) prior to acceptance.

Please note that you must retain a copy of all uncropped **original experimental data at high resolution for five years, to be shown upon request by the journal. Failure to produce such data on request can result in a retraction by the journal.**

- AUTHOR LIST:
Please review the author list to verify that it is complete and accurate. We ask that you consult with your co-authors to ensure that all names, affiliations, and titles are represented correctly. Should such details need correcting after publication, this will require a formal correction to be issued which should be avoided.
Please note, changes to authorship (addition or removal) will not be allowed once the manuscript has been formally accepted for publication.

For papers containing one or more consortia, all members of the consortium who contributed to the paper must be listed in the paper (i.e., print/online PDF). If necessary, individual authors can be listed in both the main author list and as a member of a consortium listed at the end of the paper. When submitting your revised manuscript via the online submission system, the consortium name should be entered as an author, together with the contact details of a nominated consortium representative. See our authorship policy and further consortia formatting guidelines, which should be adhered to prior to acceptance.

2. PUBLICATION REQUIREMENTS:

Pending submission of your final files, you will be contacted following the acceptance of your manuscript to complete the relevant publication and payment forms for your manuscript, hence you do not need to return any such forms with your final files.

3. ORCID:

Cell Death & Disease is committed to improving transparency in authorship. As part of our efforts in this direction, we are now requesting that all authors identified as 'corresponding author' create and link their Open Researcher and Contributor Identifier (ORCID) with their account on the Manuscript Tracking System (MTS) prior to acceptance. ORCID helps the scientific community achieve unambiguous attribution of all scholarly contributions. For more information please visit http://www.springernature.com/orcid

For all corresponding authors listed on the manuscript, please follow the instructions in the link below to link your ORCID to your account on our MTS before submitting the final version of the manuscript. If you do not yet have an ORCID you will be able to create one in minutes.
https://www.springernature.com/gp/researchers/orcid/orcid-for-nature-research.

IMPORTANT: All authors identified as 'corresponding author' on the manuscript must follow these instructions. Non-corresponding authors do not have to link their ORCIDs but are encouraged to do so. Please note that it will not be possible to add/modify ORCIDs at proof. Thus, if they wish to have their ORCID added to the paper they must also follow the above procedure prior to acceptance.

To support ORCID's aims, we only allow a single ORCID identifier to be attached to one account. If you have any issues attaching an ORCID identifier to your MTS account, please contact the Platform Support Helpdesk.

You can now use a single sign-on for all your accounts, view the status of all your manuscript submissions and reviews, access usage statistics for your published articles and download a record of your refereeing activity for the Nature journals.

Sincerely,

Mauro Piacentini, Yufang Shi and Hans-Uwe Simon
Editors-in-Chief,
Cell Death & Disease

--

Reviewer Comments:

This email has been sent through the NPG Manuscript Tracking System NY-610A-NPG&MTS

Confidentiality Statement:

This e-mail is confidential and subject to copyright. Any unauthorised use or disclosure of its contents is prohibited. If you have received this email in error please notify our Manuscript Tracking System Helpdesk team at http://platformsupport.nature.com .

Details of the confidentiality and pre-publicity policy may be found here http://www.nature.com/authors/policies/confidentiality.html

Privacy Policy | Update Profile

黃智洋 <flylerd0425@gmail.com> 2023年12月15日 上午10:29
收件者: 趙坤山院長 <d94032@mail.cmuh.org.tw>, 自諒 陳 <golfoma22@gmail.com>, 道維 柯 <ketaowei@gmail.com>, 梁基安 <d4615@mail.cmuh.org.tw>, 陳宗偉 <runrunrungo@yahoo.com.tw>, 姜淑芬 <suvenchiang@gmail.com>, 彭馨蕾 <speng@mail.cmu.edu.tw>, 張慧蘋 <winter12snow84@gmail.com>, 林佩君 <catlovefish0907@gmail.com>, 楊珮禎 <janeyang001015@gmail.com>, 洪維澤 <b23750046@gmail.com>

Dear all,

The paper entitled "TNFa modulates PANX1 activation to promote ATP release and enhance P2RX7-mediated antitumor immune responses after chemotherapy in colorectal cancer" has been **provisionally accepted** for publication in *Cell Death & Disease.*

Please confirm the correct spelling of your name and the author list
Kevin Chih-Yang Huang, Shu-Fen Chiang, Pei-Chun Lin, Wei-Ze Hong, Pei-Chen Yang, Hui-Ping Chang, Shin-Lei Peng, Tsung-Wei Chen, Tao-Wei Ke, Ji-An Liang, William Tzu-Liang Chen*, K. S. Clifford Chao*

If there is no problem, please reply to confrim. Thank you.

Best regrads,

Chih-Yang

---------- Forwarded message ---------
寄件者： <cddisease@springernature.com>
Date: 2023年12月5日 週二 上午12:19
Subject: Decision letter for CDDIS-23-1970RR: Accepted in Principle
To: <flylerd0425@gmail.com>
[隱藏引用文字]

--
Kevin Chih-Yang Huang, Ph.D
Associate Professor/Associate Research Fellow
Department of Biomedical Imaging and Radiological Science, China Medical University
Translation Research Core, China Medical University Hospital
Taichung, Taiwan
E-mail: flylerd0425@gmail.com /096752@tool.caaumed.org.tw / chihyang0425@mail.cmu.edu.tw

黃智洋 副教授兼副研究員
生物醫學影像暨放射科學學系/中國醫藥大學
轉譯研究核心/中國醫藥大學附設醫院
404台中市北區學士路91號 立夫教學大樓10樓
Tel：04-22052121 ext. 12074
E-mail: flylerd0425@gmail.com /096752@tool.caaumed.org.tw /chihyang0425@mail.cmu.edu.tw

---

彭馨蕾 <speng@mail.cmu.edu.tw> 2023年12月15日 上午10:55
收件者: flylerd0425@gmail.com

沒有問題．謝謝黃老師的幫忙。

馨蕾

[隱藏引用文字]
[隱藏引用文字]
[隱藏引用文字]
[隱藏引用文字]
original experimental data at high resolution for five years, to be shown upon request by the journal. Failure to produce such data on request can result in a retraction by the journal.
[隱藏引用文字]
?

This email has been sent through the NPG Manuscript Tracking System NY-610A-NPG&MTS

Confidentiality Statement:

This e-mail is confidential and subject to copyright. Any unauthorised use or disclosure of its contents is prohibited. If you have received this email in error please notify our Manuscript Tracking System Helpdesk team at http://platformsupport.nature.com .

Details of the confidentiality and pre-publicity policy may be found here http://www.nature.com/authors/policies/confidentiality.html

Privacy Policy | Update Profile

[隱藏引用文字]

Pei-Chun Lin <catlovefish0907@gmail.com> 2023年12月15日 上午11:03
收件者: 黃智洋 <flylerd0425@gmail.com>

學長午安

我這裡沒有問題

佩君

黃智洋 <flylerd0425@gmail.com> 於 2023年12月15日 週五 上午10:30寫道：
[隱藏引用文字]

Clifford Chao <biotechgateway@gmail.com> 2023年12月15日 下午1:56
收件者: 黃智洋 <flylerd0425@gmail.com>
副本: 趙坤山院長 <d94032@mail.cmuh.org.tw>, 自諒 陳 <golfoma22@gmail.com>, 道維 柯 <ketaowei@gmail.com>, 梁基安 <d4615@mail.cmuh.org.tw>, 陳宗偉 <runrunrungo@yahoo.com.tw>, 姜淑芬 <suvenchiang@gmail.com>, 彭馨蕾 <speng@mail.cmu.edu.tw>, 張慧蘋 <winter12snow84@gmail.com>, 林佩君 <catlovefish0907@gmail.com>, 楊珮禎 <janeyang001015@gmail.com>, 洪維澤 <b23750046@gmail.com>

A great news, Kevin. . Thanks to all for a fantastic team work.
Cliff
[隱藏引用文字]

Sawa Jukain <b23750046@gmail.com> 2023年12月15日 下午2:52
收件者: 黃智洋 <flylerd0425@gmail.com>

Confirm.

黃智洋 <flylerd0425@gmail.com> 於 2023年12月15日 週五 上午10:30寫道：
[隱藏引用文字]

---

feno Chiang <suvenchiang@gmail.com> 2023年12月17日 晚上8:27
收件者: 黃智洋 <flylerd0425@gmail.com>

The author list is correct.
[隱藏引用文字]

---

runrunrungo <runrunrungo@yahoo.com.tw> 2023年12月18日 清晨7:56
收件者: 黃智洋 <flylerd0425@gmail.com>

Correct.

黃智洋 <flylerd0425@gmail.com> 於 2023年12月15日 週五 上午10:29寫道：
[隱藏引用文字]
[隱藏引用文字]

楊珮禎 <janeyang001015@gmail.com> 2023年12月18日 晚上9:11
收件者: 黃智洋 <flylerd0425@gmail.com>

Confirm

黃智洋 <flylerd0425@gmail.com> 於 2023年12月15日 上午10:29 寫道：

[隱藏引用文字]

黃智洋 <flylerd0425@gmail.com> 2023年12月19日 清晨7:44
收件者: 黃智洋 <flylerd0425@gmail.com>

Okay

從我的iphone 傳送

黃智洋 <flylerd0425@gmail.com> 於 2023年12月18日 晚上9:11 寫道：

[隱藏引用文字]

張慧蘋 <winter12snow84@gmail.com> 2023年12月19日 上午9:11
收件者: flylerd0425@gmail.com

Correct

從我的iphone 傳送

黃智洋 <flylerd0425@gmail.com> 於 2023年12月19日 清晨7:44 寫道：

Okay
[隱藏引用文字]

梁基安 <d4615@mail.cmuh.org.tw> 2023年12月19日 下午3:50
收件者: 黃智洋 <flylerd0425@gmail.com>

Okay.

黃智洋 <flylerd0425@gmail.com> 於 2023年12月15日 週五 上午10:29寫道：
Dear all,
[隱藏引用文字]
[隱藏引用文字]
[隱藏引用文字]

William Chen <golfoma22@gmail.com> 2023年12月19日 晚上10:41
收件者: 黃智洋 <flylerd0425@gmail.com>

Dear Kevin,

Congratulation!
The author list is okay to me.

Best regrardes,

黃智洋 <flylerd0425@gmail.com> 於 2023年12月15日 週五 上午10:29寫道：
Dear all,
[隱藏引用文字]
[隱藏引用文字]
[隱藏引用文字]

David Ke <ketaowei@gmail.com>
收件者: 黃智洋 <flylerd0425@gmail.com> 2023年12月20日 上午8:21

Congratulation to you! Kevin

Thank you for your work team.

The authorship and spelling is okay.

Best regards,

黃智洋 <flylerd0425@gmail.com> 於 2023年12月15日 週五 上午10:29寫道：

Dear all,

[隱藏引用文字]

[隱藏引用文字]

[隱藏引用文字]
