# Supplementary material for: TNFα modulates PANX1 activation to promote ATP release and enhance P2RX7-mediated antitumor immune responses after chemotherapy in colorectal cancer
Source: Cell Death Dis. 2024 Jan 9;15(1):24. doi: 10.1038/s41419-023-06408-5 (PMC10776587; doi:10.1038/s41419-023-06408-5)
Supplement: Supplementary file 2 — supplementary information [file 41419_2023_6408_MOESM2_ESM.docx]

**Supporting information**

**TNFα modulates PANX1 activation for ATP release to enhance P2RX7-mediated antitumor immunity by chemotherapy against colorectal cancer**

| **Table S1. The cytotoxic ability of TNF-alpha on colorectal cancer cells** | | | | | | | | | |
| --- | --- | --- | --- | --- | --- | --- | --- | --- | --- |
|  | **HCT116** |  | **HCT15** |  | **LoVo** |  | **SW480** |  | **SW620** |
| Control | 100±2.61 |  | 100.00±4.39 |  | 100.00±3.06 |  | 100.00±1.50 |  | 100.00±5.16 |
| TNFα | 60.75±4.65*** |  | 75.91±6.14** |  | 81.65±5.99** |  | 83.84±3.09*** |  | 86.65±3.55** |
| TNFα/z-DEVD-fmk | 83.10±3.99## |  | 90.56±7.12# |  | 92.31±6.94# |  | 96.24±5.35## |  | 95.00±3.61# |
| TNFα/Nec-1 | 73.05±5.62+ |  | 84.85±8.28 |  | 77.70±5.99 |  | 94.39±2.18 |  | 92.17±4.23 |
| TNFα vs Control: **p<0.01 and ***p<0.001, TNFα/z-DEVD-fmk vs TNFα:##p<0.01 and #p<0.05, TNFα/Nec-1 vs TNFα: +p<0.05 | | | | | | | | | |

| **Table S2. Clinicopathological parameters of stage III colon carcinoma patients (n=410)** | | | | | | | | | | | | | | | | | | | |
| --- | --- | --- | --- | --- | --- | --- | --- | --- | --- | --- | --- | --- | --- | --- | --- | --- | --- | --- | --- |
| Clinicopathological parameters | Total no.^a^ | TLR5 R392STOP (rs5744168) | | *p* value |  | Total no. | TLR5 F616L (rs5744174) | | *p* value |  | Total no. | P2RX7 E496A (rs3751143) | | *p* value |  | Total no. | TLR2 -196~ -174 (rs111200466) | | *p* value |
|  |  | WT | Variant |  |  |  | WT | Variant |  |  |  | WT | Variant |  |  |  | WT | Variant |  |
|  | 410 | 395 | 15 |  |  | 410 | 261 | 149 |  |  | 409 | 256 | 153 |  |  | 410 | 180 | 230 |  |
| Gender |  |  |  | 0.271 |  |  |  |  | 0.634 |  |  |  |  | 0.79 |  |  |  |  | 0.958 |
| Female | 189 | 180 (45.6%) | 9 (60%) |  |  | 189 | 118 (45.2%) | 71 (47.7%) |  |  | 189 | 117 (45.7%) | 72 (47.1%) |  |  | 189 | 83 (46.1%) | 105 (45.9%) |  |
| Male | 221 | 215 (54.4%) | 6 (40%) |  |  | 221 | 143 (54.8%) | 78 (52.3%) |  |  | 220 | 139 (54.3%) | 81 (52.9%) |  |  | 221 | 97 (53.9%) | 124 (54.1%) |  |
| Age |  |  |  | 0.634 |  |  |  |  | 0.757 |  |  |  |  | 0.179 |  |  |  |  | 0.418 |
| <65 | 194 | 186 (47.1%) | 8 (53.3%) |  |  | 194 | 125 (47.9%) | 69 (46.3%) |  |  | 194 | 128 (50%) | 66 (43.1%) |  |  | 194 | 89 (49.4%) | 104 (45.4%) |  |
| ≥65 | 216 | 209 (52.9%) | 7 (46.7%) |  |  | 216 | 136 (52.1%) | 80 (53.7%) |  |  | 215 | 128 (50%) | 87 (56.9%) |  |  | 216 | 91 (50.6%) | 125 (54.6%) |  |
| Tumor location |  |  |  | 0.036 |  |  |  |  | 0.587 |  |  |  |  | 0.008 |  |  |  |  | 0.859 |
| Distal colon | 210 | 198 (50.1%) | 12 (80%) |  |  | 210 | 131 (50.2%) | 79 (53.0%) |  |  | 210 | 119 (46.5%) | 91 (59.5%) |  |  | 210 | 92 (51.0%) | 117 (50.9%) |  |
| Proximal colon | 197 | 194 (49.1%) | 3 (20%) |  |  | 197 | 128 (49.0%) | 69 (46.3%) |  |  | 196 | 136 (53.1%) | 60 (39.2%) |  |  | 197 | 85 (47.2%) | 112 (48.7%) |  |
| Unspecified | 3 | 3 (0.8%) | 0 (0%) |  |  | 3 | 2 (0.8%) | 1 (0.7%) |  |  | 3 | 1 (0.4%) | 2 (1.3%) |  |  | 3 | 2 (1.1%) | 1 (0.4%) |  |
| pT stage |  |  |  | 0.89 |  |  |  |  | 0.383 |  |  |  |  | 0.87 |  |  |  |  | 0.171 |
| pT1-2 | 31 | 30 (7.6%) | 1 (6.7%) |  |  | 31 | 22 (8.4%) | 9 (6.0%) |  |  | 31 | 19 (7.4%) | 12 (7.9%) |  |  | 31 | 10 (5.6%) | 21 (9.1%) |  |
| pT3-4 | 377 | 363 (91.9%) | 14 (93.3%) |  |  | 377 | 238 (91.2%) | 139 (93.3%) |  |  | 376 | 236 (92.2%) | 140 (92.1%) |  |  | 376 | 169 (93.8%) | 207 (90.%) |  |
| Undefined | 2 | 2 (0.5%) | 0 (0%) |  |  | 2 | 1 (0.4%) | 1 (0.7%) |  |  | 2 | 1 (0.4%) | 1 (.07%) |  |  | 3 | 1 (0.6%) | 2 (0.9%) |  |
| Tumor differentiation |  |  |  | 0.78 |  |  |  |  | 0.69 |  |  |  |  | 0.27 |  |  |  |  | 0.21 |
| Well to moderate | 402 | 387 (98.0%) | 15 (100%) |  |  | 402 | 256 (98.1%) | 146 (98.0%) |  |  | 401 | 249 (97.2%) | 152 (99.3%) |  |  | 401 | 175 (97.2%) | 226 (98.3%) |  |
| Poor | 2 | 2 (0.5%) | 0 (0%) |  |  | 2 | 1 (0.4%) | 1 (0.7%) |  |  | 2 | 2 (0.8%) | 0 (0%) |  |  | 2 | 0 (0%) | 2 (0.9%) |  |
| Unknown | 6 | 6 (1.5%) | 0 (%) |  |  | 6 | 4 (1.5%) | 2 (1.3%) |  |  | 6 | 5 (2.0%) | 1 (0.7%) |  |  | 7 | 5 (2.8%) | 2 (0.9%) |  |
| Lymphovascular invasion |  |  |  | 0.911 |  |  |  |  | 0.589 |  |  |  |  | 0.913 |  |  |  |  | 0.37 |
| Absent | 114 | 110 (28%) | 4 (26.7%) |  |  | 114 | 75 (28.7%) | 39 (26.1%) |  |  | 113 | 71 (27.7%) | 42 (27.5%) |  |  | 113 | 54 (30%) | 59 (25.7%) |  |
| Present | 294 | 283 (71.5%) | 11 (73.3%) |  |  | 294 | 185 (70.9%) | 109 (73.2%) |  |  | 294 | 183 (71.5%) | 111 (72.5%) |  |  | 294 | 126 (70%) | 168 (71%) |  |
| Unknown | 2 | 2 (0.5%) | 0 (0%) |  |  | 2 | 1 (0.4%) | 1 (0.7%) |  |  | 2 | 2 (0.8%) | 0 (0%) |  |  | 3 | 0 (0%) | 3 (1.3%) |  |
| Perineural invasion |  |  |  | 0.23 |  |  |  |  | 0.971 |  |  |  |  | 0.35 |  |  |  |  | 0.221 |
| Absent | 210 | 200 (50.6%) | 10 (66.7%) |  |  | 210 | 134 (51.3%) | 76 (51.0%) |  |  | 209 | 136 (53.1%) | 73 (47.7%) |  |  | 210 | 99 (55%) | 111 (48.3%) |  |
| Present | 198 | 193 (48.9%) | 5 (33.3%) |  |  | 198 | 126 (48.3%) | 72 (48.3%) |  |  | 198 | 118 (46.1%) | 80 (52.3%) |  |  | 197 | 81 (45%) | 116 (50.4%) |  |
| unknown | 2 | 2 (0.5%) | 0 (0%) |  |  | 2 | 1 (0.4%) | 1 (0.7%) |  |  | 2 | 2 (0.8%) | 0 (0%) |  |  | 3 | 0 (0%) | 3 (1.3%) |  |
| MMR status |  |  |  | 0.928 |  |  |  |  | 0.169 |  |  |  |  | 0.809 |  |  |  |  | 0.213 |
| MMR-proficient | 387 | 373 (94.4%) | 14 (93.3%) |  |  | 387 | 248 (95%) | 139 (93.3%) |  |  | 386 | 242 (94.5%) | 144 (94.1%) |  |  | 386 | 168 (93.3%) | 218 (94.8%) |  |
| MMR-deficient | 21 | 20 (5.1%) | 1 (6.7%) |  |  | 21 | 13 (5%) | 8 (5.4%) |  |  | 21 | 13 (5.1%) | 8 (5.2%) |  |  | 21 | 12 (6.7%) | 9 (3.9%) |  |
| Unknown | 2 | 2 (0.5%) | 0 (0%) |  |  | 2 | 0 (0%) | 2 (1.3%) |  |  | 2 | 1 (0.4%) | 1 (0.7%) |  |  | 3 | 0 (0%) | 3 (1.3%) |  |
| TLR5 R392STOP: WT (GG genotype) and Variant (GT heterogenotype); TLR5 F616L: WT (AA genotype) and Variant (AG heterogenotype and GG homogenotype). ^a^Number of cases may differ due to missing data. The unknown and unspecified group was not included. Fisher's exact test was used when >25% of the cells have expected counts less than 5. | | | | | | | | | | | | | | | | | | | |

| **Table S3. Correlation between clinicopathologic parameters, 5-year DMFS, 5-year DFS and 5-year OS** | | | | | | | | | |
| --- | --- | --- | --- | --- | --- | --- | --- | --- | --- |
| **Parameters** | **No^a^** | **5-year DMFS %** | ***p* value*** |  | **5-year DFS %** | ***p* value*** |  | **5-year OS %** | ***p* value*** |
|  |  | 58.3% |  |  | 53.5% |  |  | 63.7% |  |
| Sex |  |  | 0.926 |  |  | 0.878 |  |  | 0.853 |
| Female | 189 | 57.7% |  |  | 56.6% |  |  | 63.5% |  |
| Male | 221 | 58.8% |  |  | 57.9% |  |  | 63.8% |  |
| Age |  |  | 0.002 |  |  | 0.001 |  |  | <0.001 |
| <65 | 194 | 66.5% |  |  | 66.0% |  |  | 73.7% |  |
| ≥65 | 216 | 50.9% |  |  | 49.5% |  |  | 54.6% |  |
| pT stage |  |  | 0.001 |  |  | 0.001 |  |  | 0.006 |
| pT1-2 | 31 | 87.1% |  |  | 87.1% |  |  | 87.1% |  |
| pT3-4 | 377 | 55.7% |  |  | 54.6% |  |  | 61.5% |  |
| Tumor location |  |  | 0.014 |  |  | 0.027 |  |  | 0.02 |
| Distal colon | 210 | 63.8% |  |  | 62.4% |  |  | 69.0% |  |
| Proximal colon | 197 | 52.8% |  |  | 52.3% |  |  | 58.4% |  |
| Tumor differentiation |  |  | 0.606 |  |  | 0.628 |  |  | 0.498 |
| Well to moderate | 402 | 58.2% |  |  | 57.2% |  |  | 63.4% |  |
| Poor | 2 | 50.0% |  |  | 50.0% |  |  | 50.0% |  |
| Lymphovascular invasion |  |  | 0.008 |  |  | 0.040 |  |  | 0.015 |
| Absent | 114 | 69.3% |  |  | 69.3% |  |  | 73.7% |  |
| Present | 294 | 54.1% |  |  | 52.7% |  |  | 59.9% |  |
| Perineural invasion |  |  | 0.016 |  |  | 0.009 |  |  | 0.038 |
| Absent | 210 | 64.8% |  |  | 64.3% |  |  | 69.5% |  |
| Present | 198 | 51.5% |  |  | 50.0% |  |  | 57.6% |  |
| TLR5 R392STOP (rs5744168) |  |  | 0.484 |  |  | 0.427 |  |  | 0.211 |
| WT | 395 | 58.0% |  |  | 57.0% |  |  | 63.0% |  |
| Variant | 15 | 66.7% |  |  | 66.7% |  |  | 80.0% |  |
| TLR5 F616L (rs5744174) |  |  | 0.487 |  |  | 0.690 |  |  | 0.33 |
| WT | 261 | 56.7% |  |  | 56.3% |  |  | 61.7% |  |
| Variant | 149 | 61.1% |  |  | 59.1% |  |  | 67.1% |  |
| P2RX7 E496A (rs3751143) |  |  | 0.031 |  |  | 0.043 |  |  | 0.15 |
| WT | 256 | 62.1% |  |  | 60.9% |  |  | 66.0% |  |
| Variant | 153 | 51.6% |  |  | 51.0% |  |  | 59.5% |  |
| TLR2 -196~ -174 (rs111200466) |  |  | 0.739 |  |  | 0.524 |  |  | 0.587 |
| WT | 180 | 59.4% |  |  | 59.4% |  |  | 65.6% |  |
| Variant | 229 | 57.2% |  |  | 55.5% |  |  | 62.0% |  |
| P2RX7 E496A (rs3751143)/CD8+TILs |  |  | 0.003 |  |  | 0.002 |  |  | 0.025 |
| WT and High | 76 | 73.7% |  |  | 73.7% |  |  | 75.0% |  |
| Variant or Low | 333 | 54.7% |  |  | 53.5% |  |  | 61.0% |  |
| P2RX7 E496A (rs3751143)/CD45RO+TILs |  |  | 0.001 |  |  | 0.001 |  |  | 0.003 |
| WT and High | 99 | 72.7% |  |  | 72.7% |  |  | 76.8% |  |
| Variant or Low | 311 | 53.7% |  |  | 52.4% |  |  | 59.5% |  |
| ^a^Number of cases may differ due to missing data. | | | | | | | | | |

| **Table S4. The association between P2RX7-E496A and intratumoral-infiltrating lymphocytes (TILs)** | | | | |
| --- | --- | --- | --- | --- |
| Clinicopathological parameters | Total no. | P2RX7-E496A (rs3751143) | | *p* value |
|  |  | WT | Variant |  |
|  | 409 | 256 | 153 |  |
| CD45+TILs |  |  |  | 0.251 |
| High | 159 | 105 (41%) | 54 (35.3%) |  |
| Low | 250 | 151 (59%) | 99 (64.7%) |  |
| CD8+TILs |  |  |  | 0.036 |
| High | 107 | 76 (29.7%) | 31 (20.3%) |  |
| Low | 302 | 180 (70.3%) | 122 (79.7%) |  |
| CD45RO+TILs |  |  |  | 0.046 |
| High | 142 | 98 (38.7%) | 44 (28.9%) |  |
| Low | 263 | 155 (61.3%) | 108 (71.1%) |  |
| PD1+TILs |  |  |  | 0.445 |
| High | 35 | 24 (9.4%) | 11 (7.2%) |  |
| Low | 274 | 232 (90.6%) | 142 (92.8%) |  |
|  | | | | |

| **Table S5. Univariate and multivariate analysis of 5-years DFS and known prognostic factors in stage III colon carcinoma patients.** | | | | | | | | | | | |
| --- | --- | --- | --- | --- | --- | --- | --- | --- | --- | --- | --- |
| **Parameters** | **Univariate analysis** | | | | |  | **Multivariate analysis** | | | | |
|  | **No. at risk^a^** | **Events** | **HR** | **95% CI** | ***p* value** |  | **No. at risk^a^** | **Events** | **HR** | **95% CI** | ***p* value** |
| Sex |  |  |  |  | 0.878 |  |  |  |  |  |  |
| Female | 189 | 82 | 1 |  |  |  |  |  |  |  |  |
| Male | 221 | 93 | 1.0 | 0.726-1.32 |  |  |  |  |  |  |  |
| Age |  |  |  |  | **0.001** |  |  |  |  |  | **0.006** |
| <65 | 194 | 66 | 1 |  |  |  | 194 | 66 | 1.00 |  |  |
| ≥65 | 216 | 109 | 1.646 | 1.212-2.24 |  |  | 216 | 109 | 1.55 | 1.14-2.11 |  |
| pT stage |  |  |  |  | **0.002** |  |  |  |  |  | **0.010** |
| pT1-2 | 31 | 4 | 1 |  |  |  | 31 | 4 | 1.00 | 31 |  |
| pT3-4 | 377 | 171 | 4.631 | 1.718-12.5 |  |  | 377 | 171 | 3.74 | 1.37-10.20 |  |
| Tumor location |  |  |  |  | **0.027** |  |  |  |  |  | **0.014** |
| Distal colon | 210 | 79 | 1 |  |  |  | 210 | 79 | 1.00 |  |  |
| Proximal colon | 197 | 94 | 1.401 | 1.038-1.89 |  |  | 197 | 94 | 1.55 | 1.08-2.00 |  |
| Tumor differentiation |  |  |  |  | 0.631 |  |  |  |  |  |  |
| Well to moderate | 402 | 172 | 1 |  |  |  |  |  |  |  |  |
| Poor | 2 | 1 | 1.618 | 0.227-11.6 |  |  |  |  |  |  |  |
| Lymphovascular invasion |  |  |  |  | **0.004** |  |  |  |  |  | 0.081 |
| Absent | 114 | 35 | 1 |  |  |  | 114 | 35 | 1.00 |  |  |
| Present | 294 | 139 | 1.717 | 1.185-2.49 |  |  | 294 | 139 | 1.40 | 0.96-2.05 |  |
| Perineural invasion |  |  |  |  | **0.009** |  |  |  |  |  | 0.332 |
| Absent | 210 | 75 | 1 |  |  |  | 210 | 75 | 1.00 |  |  |
| Present | 198 | 99 | 1.49 | 1.104-20.1 |  |  | 198 | 99 | 1.17 | 0.86-1.59 |  |
| P2RX7 E496A (rs3751143) |  |  |  |  | **0.017** |  |  |  |  |  | **0.014** |
| WT | 256 | 98 | 1 |  |  |  | 256 | 98 | 1.00 |  |  |
| Variant | 153 | 77 | 1.44 | 1.068-1.94 |  |  | 153 | 77 | 1.47 | 1.08-1.99 |  |
| CD8+TILs |  |  |  |  | **0.004** |  |  |  |  |  | **0.037** |
| High | 107 | 32 | 1 |  |  |  | 10.7 | 32 | 1.00 |  |  |
| Low | 303 | 143 | 1.751 | 1.193-2.57 |  |  | 303 | 143 | 1.52 | 1.03-2.26 |  |
| CD45RO+TILs |  |  |  |  | **0.006** |  |  |  |  |  |  |
| High | 142 | 47 | 1 |  |  |  |  |  |  |  |  |
| Low | 264. | 127 | 1.601 | 1.146-2.24 |  |  |  |  |  |  |  |
| P2RX7 E496A (rs3751143)/CD8+TILs |  |  |  |  | **0.003** |  |  |  |  |  |  |
| WT and High | 76 | 20 | 1 |  |  |  |  |  |  |  |  |
| Variant or Low | 333 | 155 | 2.035 | 1.277-3.24 |  |  |  |  |  |  |  |
| P2RX7 E496A (rs3751143)/CD45RO+TILs |  |  |  |  | **0.001** |  |  |  |  |  |  |
| WT and High | 99 | 27 | 1 |  |  |  |  |  |  |  |  |
| Variant or Low | 311 | 148 | 1.995 | 1.323-3.01 |  |  |  |  |  |  |  |


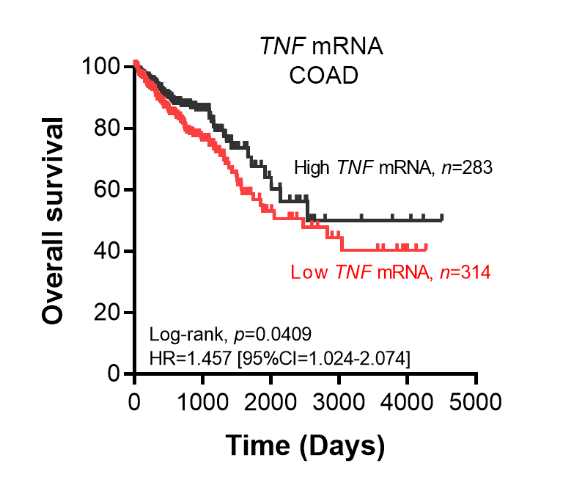


**Figure S1. Patients with high tumor TNF mRNA was associated with favorable survival outcome in COAD patients.**

The *TNF* mRNA expression was retrieved from the open-access resource named the Human Pathology Atlas as part of the Human Protein Atlas (HPA, www.proteinatlas.org/pathology).


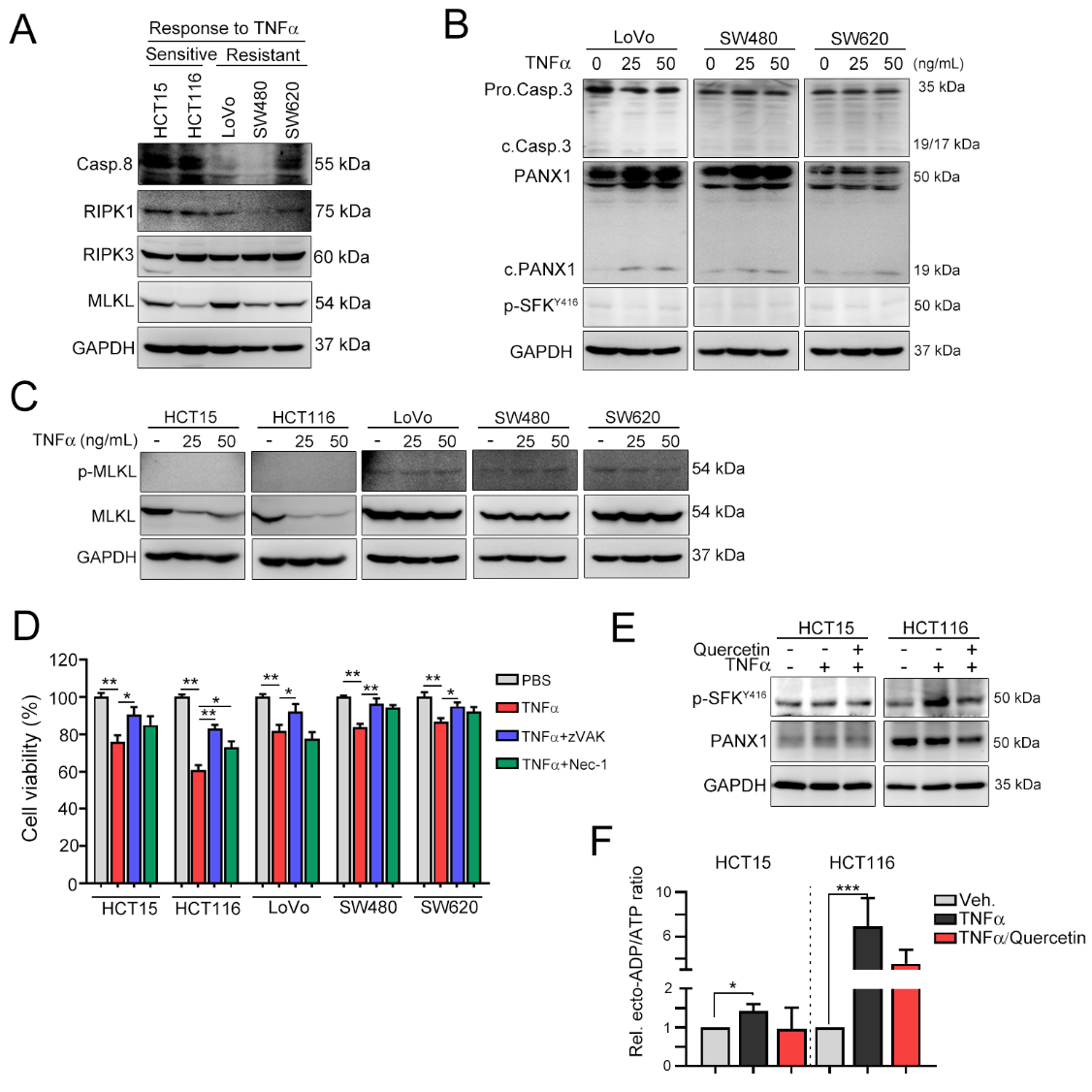


**Figure S2. MLKL signaling pathway did not involve in TNF-mediated PANX1 cleavage.**

1. The levels of TNFα-mediated two pathways for PANX1 cleavage.
2. The cleavage of PANX1 is three TNFα-resistant cell lines.
3. The level of MLKL and phosphor-MLKS was not increased by TNFα.
4. The TNFα-dependent cell viability was inhibited by apoptosis inhibitor (n=3). **p*<0.05 and ***p*<0.01.
5. HCT15 and HCT116 cells were treated with TNFα (50 ng/mL) and SFK inhibitor quercetin (10 μM) for 24 h. The level of p-PANX1 and p-SFK was evaluated by western blot.
6. HCT15 and HCT116 cells were treated with TNFα (50 ng/mL) and SFK inhibitor quercetin (10 μM) for 24 h. The extracellular ATP and ADP content was examined by luminescent ADP/ATP detection kit (n=3). **p*<0.05 and ****p*<0.001


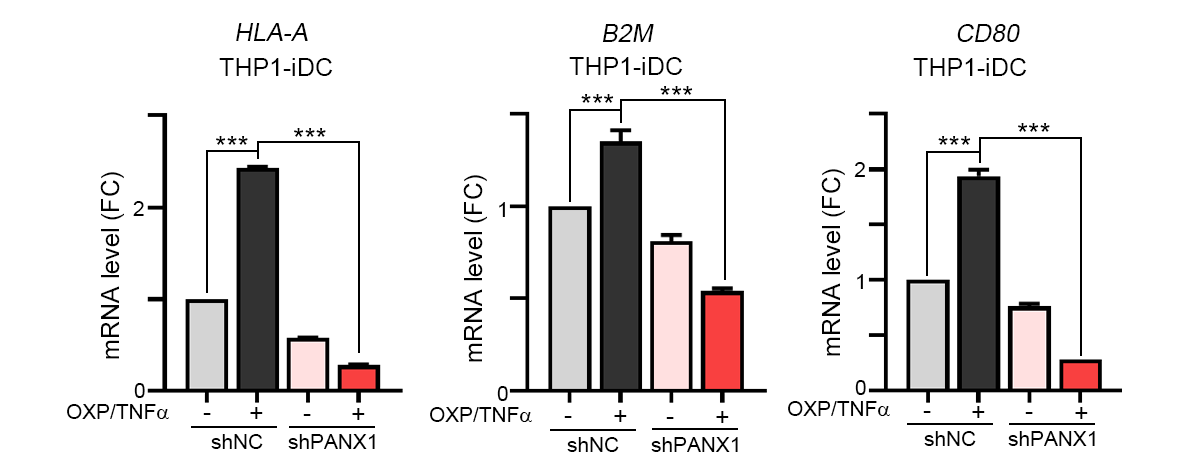


**Figure S3. Dysfunctional PANX1-mediated signaling led to insufficient DC maturation.**

The mRNA level of DC maturation marker *HLA-A, B2M and CD80* was significantly decreased in THP-iDC cells that cocultured with TNF/OXP-treated HCT116^shPANX1^(n=3). ****p*<0.001.


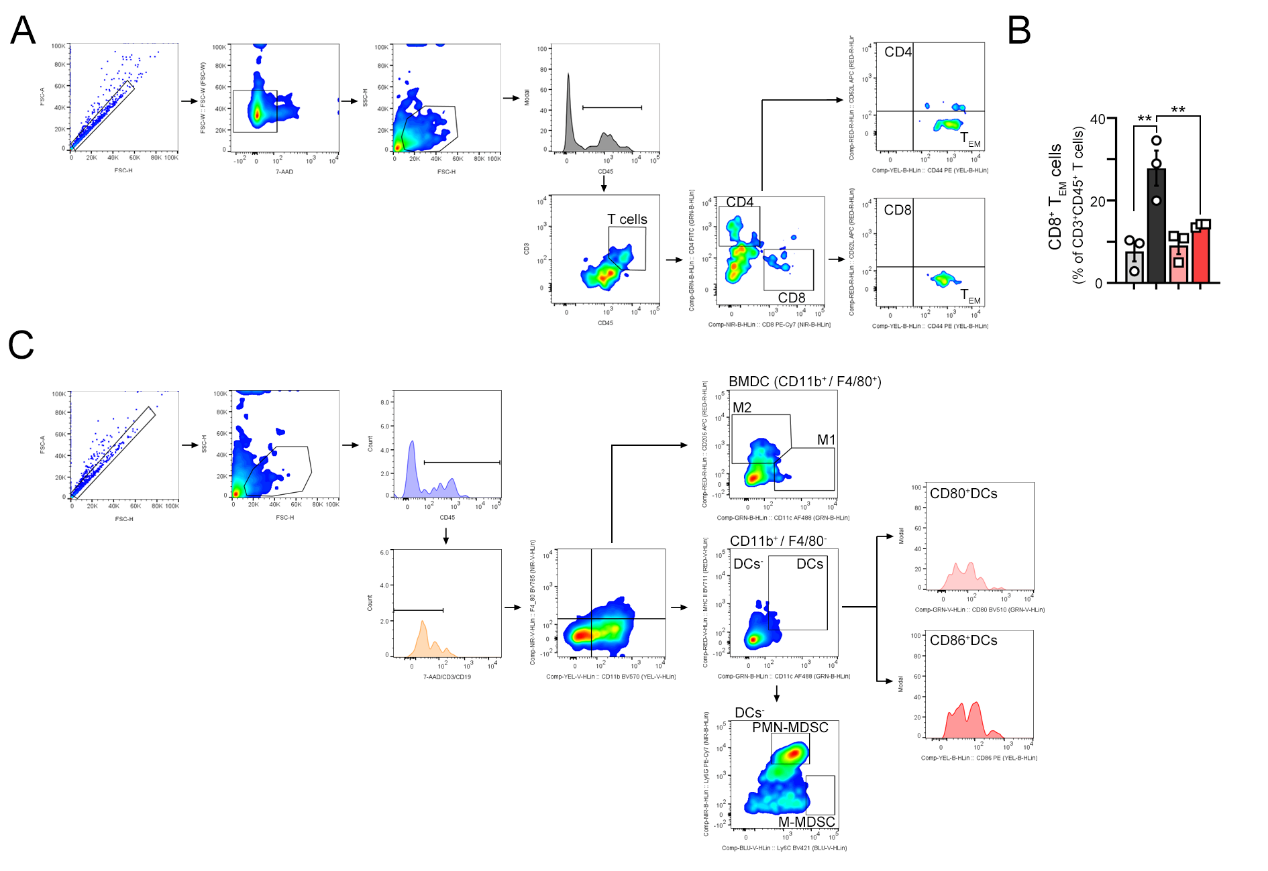


**Fig. S4. The gating strategies of immune cell profiles within resected tumors.**

1. The gating strategy for CD4, CD8 and CD4 T_EM_ and CD8 T_EM._
2. The tumor-infiltrating CD44^+^CD62L^-^CD8^+^CD3^+^ T cells within resected tumors were analyzed by flow cytometry (n=3). **p*<0.05 and ***p*<0.01.
3. The gating strategy for macrophage subtypes, dendritic cells and MDSCs_._

**
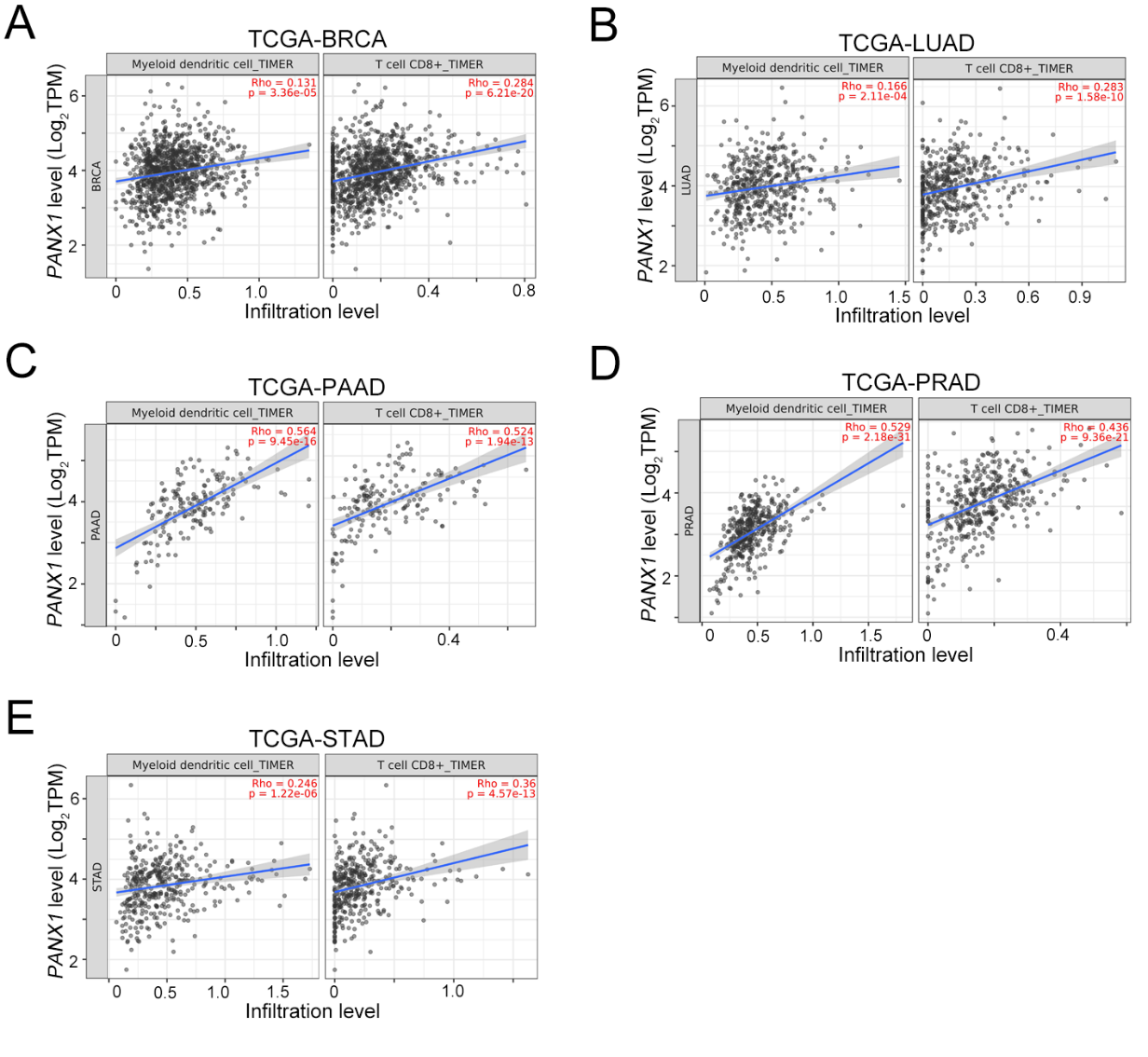
**

**Figure S5. The level of *PANX1* mRNA was positively correlated with DC and CD8a^+^ T cell signatures.**

The relationship between *PANX1* mRNA and dendritic cell (DC) and CD8a signature was analyzed in different malignancies in TCGA cohort, including breast cancer (BRCA), lung adenocarcinoma (LUAD), pancreatic adenocarcinoma (PAAD), prostate adenocarcinoma (PRAD) and stomach adenocarcinoma (STAD).


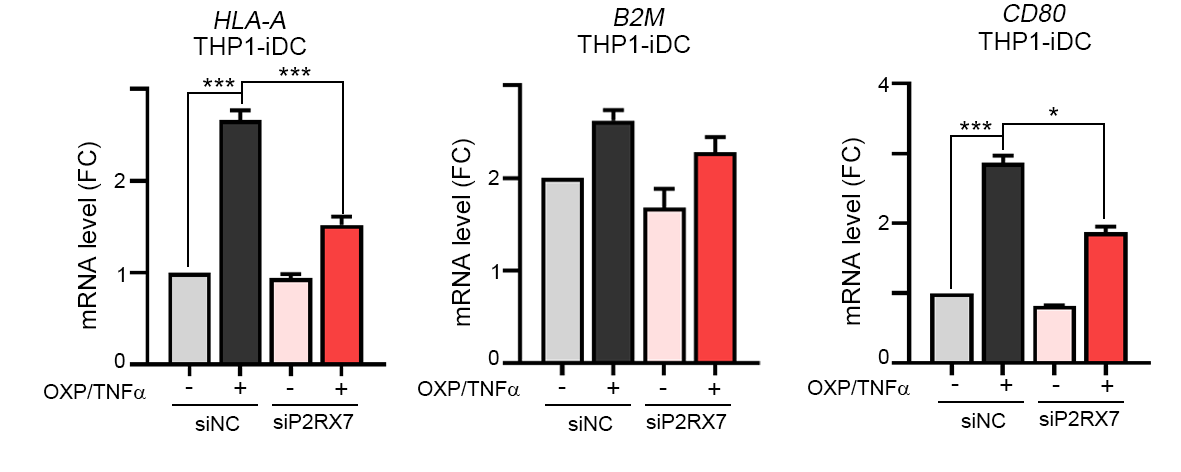


**Figure S6. Dysfunctional P2RX7-mediated signaling led to insufficient DC maturation.**

The mRNA level of DC maturation marker *HLA-A, B2M and CD80* was significantly decreased in THP-siP2RX7-iDC (n=3). **p*<0.05 and ****p*<0.001.


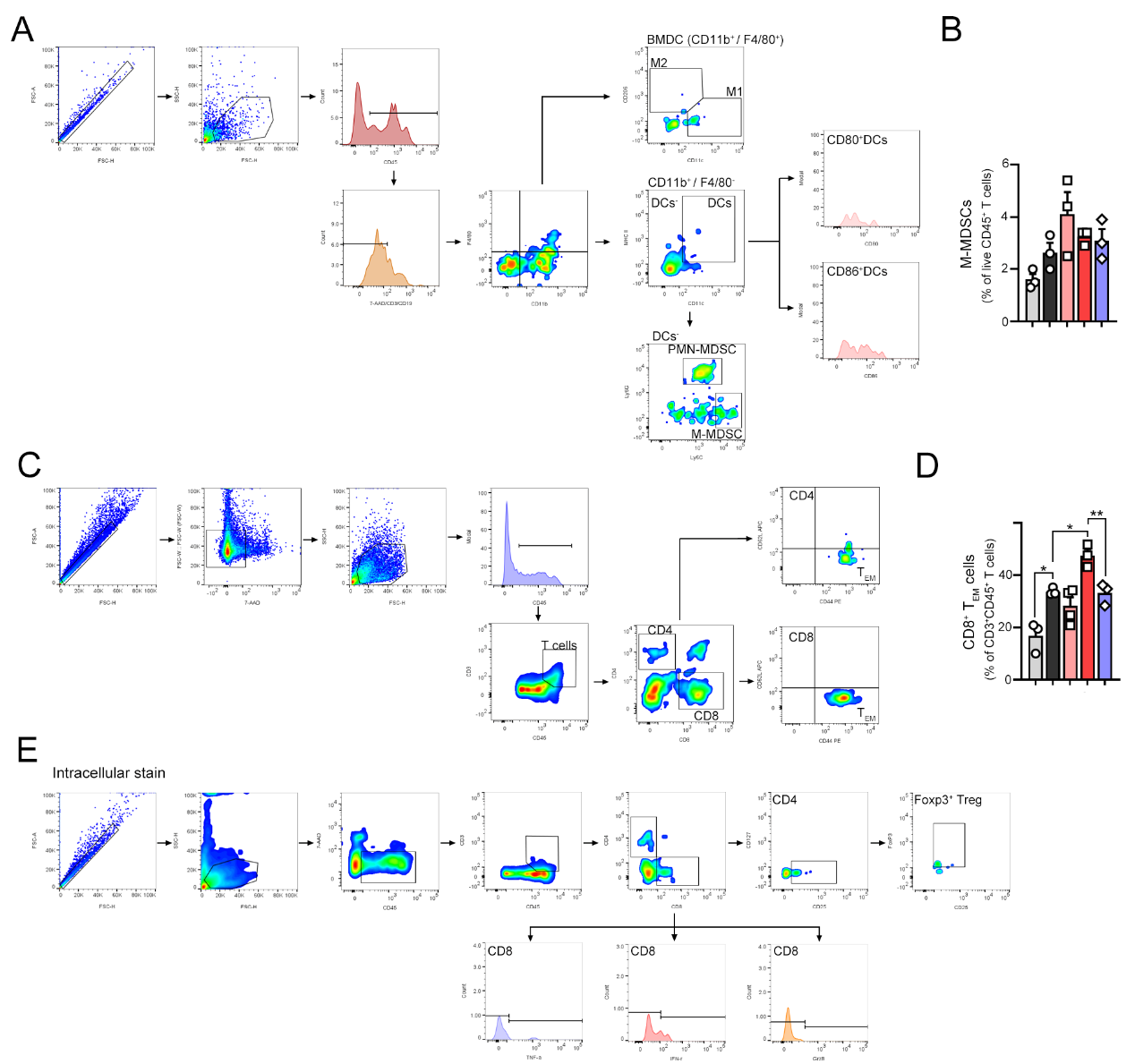


**Fig. S7. The gating strategies of immune cell profiles within resected tumors.**

1. The gating strategy for macrophage subtypes, dendritic cells and MDSCs_._
2. The tumor-infiltrating Gr-1^+^CD11b^+^ M-MDSCs within resected tumors were analyzed by flow cytometry (n=5). ***p*<0.01 and ****p*<0.001.
3. The gating strategy for CD4, CD8 and CD4 T_EM_ and CD8 T_EM._
4. The tumor-infiltrating CD44^+^CD62L^-^CD8^+^CD3^+^ T cells within resected tumors were analyzed by flow cytometry (n=3). **p*<0.05 and ***p*<0.01.
5. The gating strategy for TNFα^+^CD8^+^CD3^+^ T cells and IFNγ^+^CD8^+^CD3^+^ T cells_._


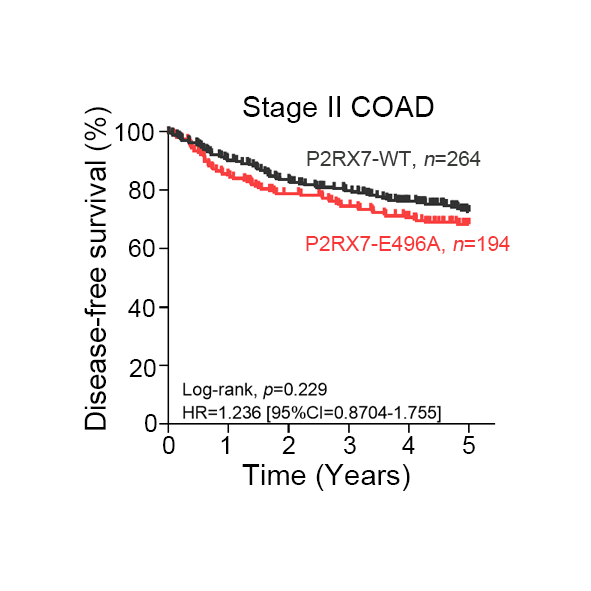


**Figure S8. The status of P2RX7-E496A was not associated with survival in stage II COAD patients.**

The status of P2RX7-E496A was not associated with survival outcome in stage II CRC patients who received adjuvant chemotherapy (n=458, Log-rank p=0.229).
