# Supplementary material for: TNFα modulates PANX1 activation to promote ATP release and enhance P2RX7-mediated antitumor immune responses after chemotherapy in colorectal cancer
Source: Cell Death Dis. 2024 Jan 9;15(1):24. doi: 10.1038/s41419-023-06408-5 (PMC10776587; doi:10.1038/s41419-023-06408-5)
Supplement: Supplementary file 3 — Supplemental Material-Raw data [file 41419_2023_6408_MOESM3_ESM.pdf]

Fig. 1B

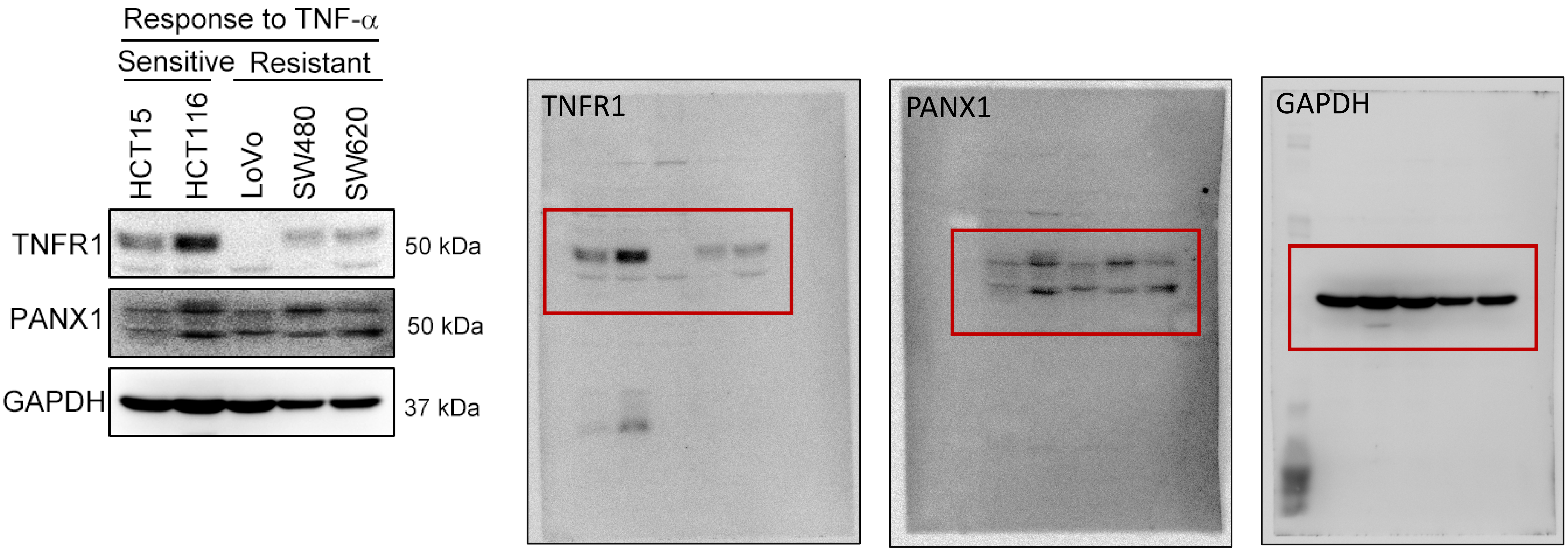
Response to TNF-α
Sensitive
Resistant
HCT15
HCT116
LoVo
SW480
SW620
TNFR1
PANX1
GAPDH
50 kDa
50 kDa
37 kDa
TNFR1
PANX1
GAPDH

Fig. 1C

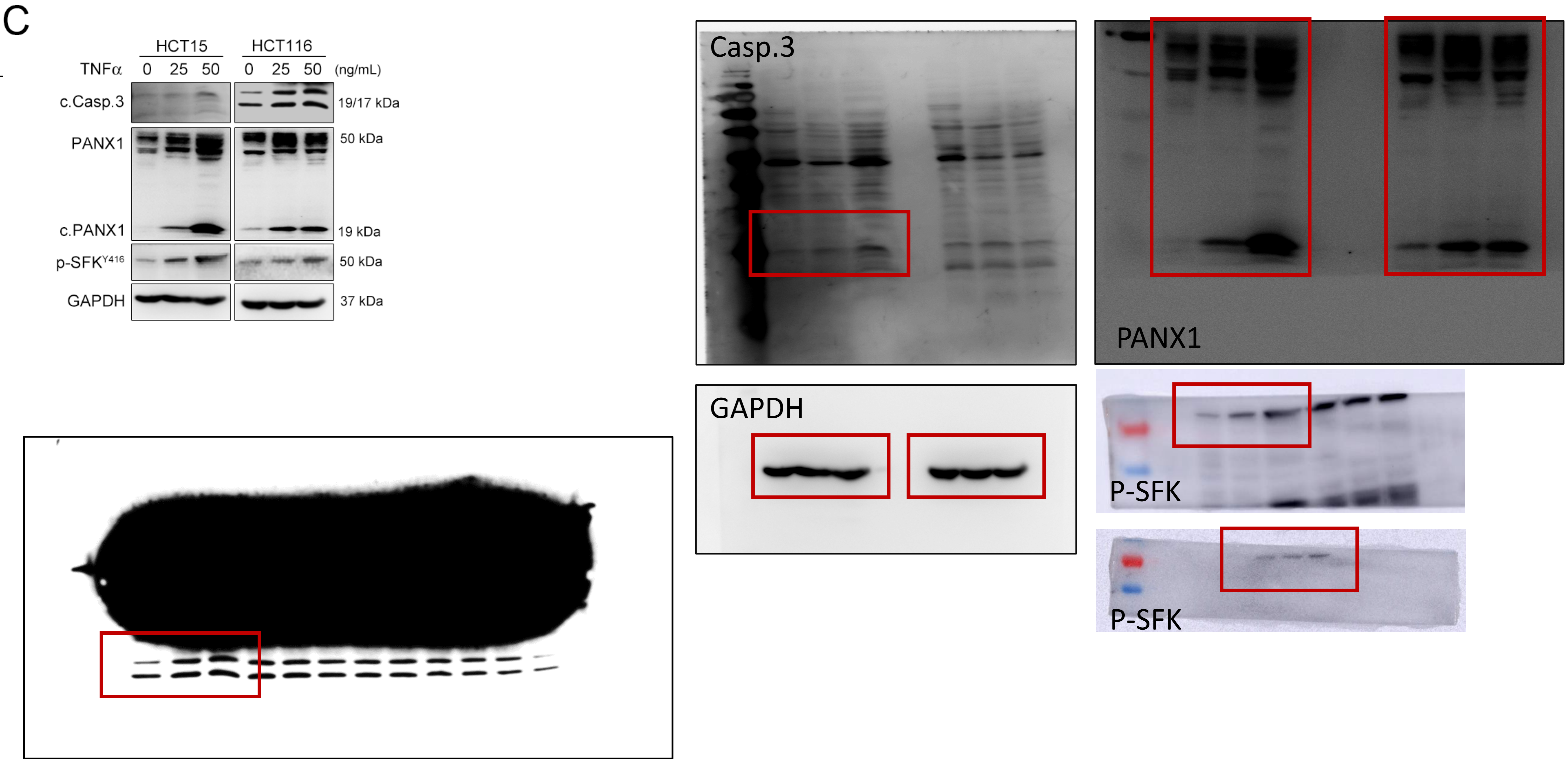
C
HCT15
HCT116
TNFα 0 25 50 0 25 50 (ng/mL)
c.Casp.3
19/17 kDa
PANX1
50 kDa
c.PANX1
19 kDa
p-SFK^{Y416}
50 kDa
GAPDH
37 kDa
Casp.3
PANX1
GAPDH
P-SFK
P-SFK

Fig. 1D

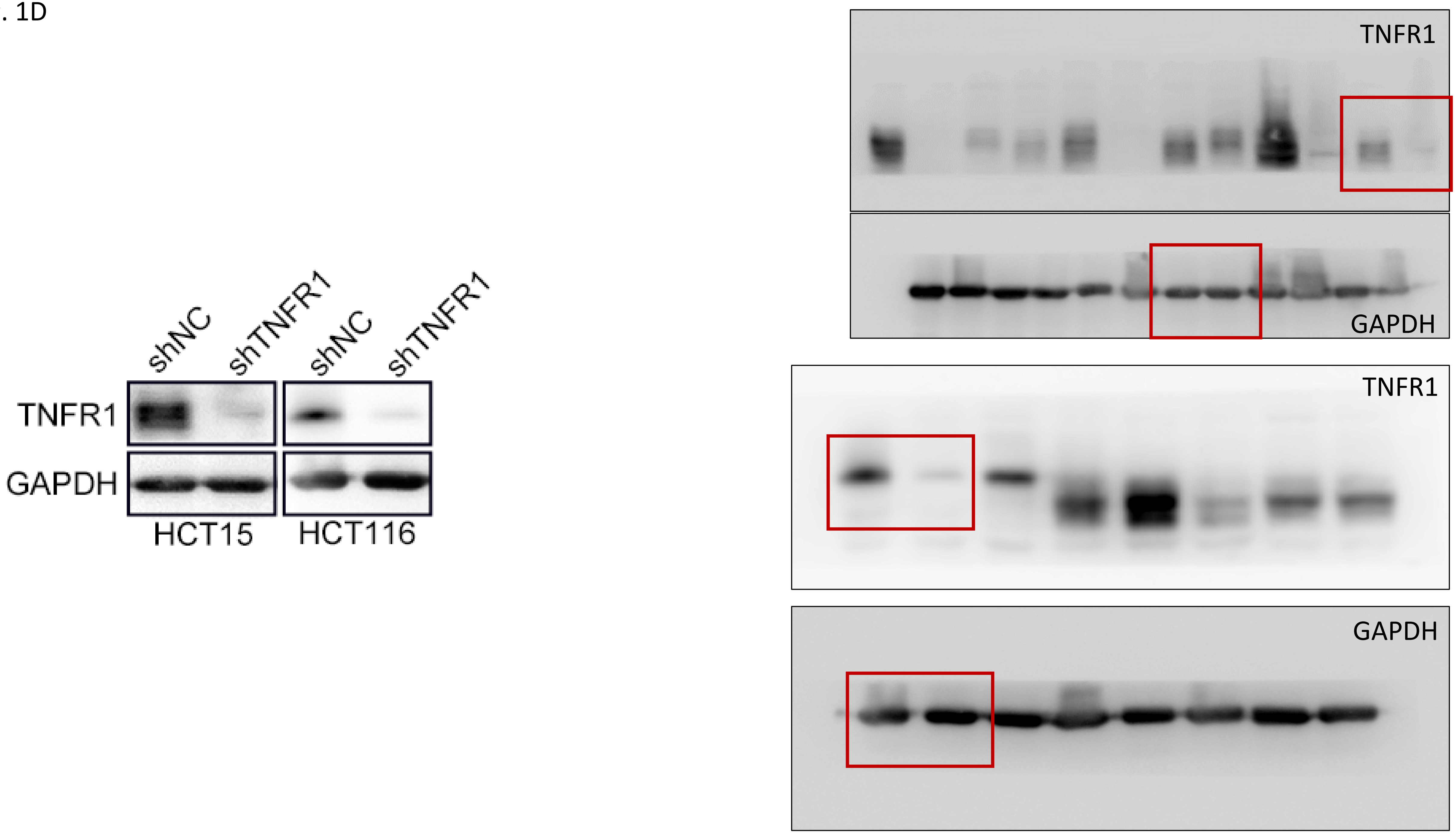

shNC
shTNFR1
shNC
shTNFR1
TNFR1
GAPDH
HCT15
HCT116
TNFR1
GAPDH
TNFR1
GAPDH

Fig. 1E

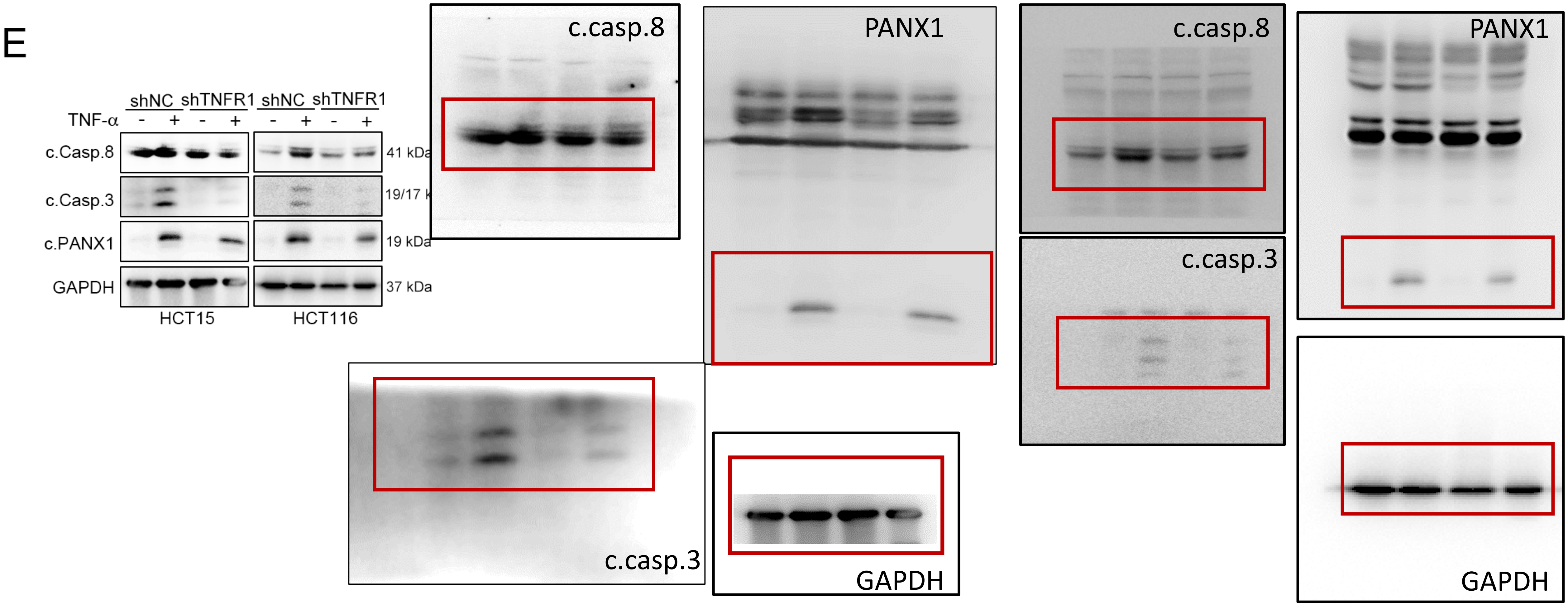
E
shNC
shTNFR1
shNC
shTNFR1
TNF-α
c.Casp.8
c.Casp.3
c.PANX1
GAPDH
41 kDa
19 kDa
37 kDa
HCT15
HCT116
c.casp.8
PANX1
c.casp.8
PANX1
c.casp.3
c.casp.3
GAPDH
GAPDH

Fig. 1F

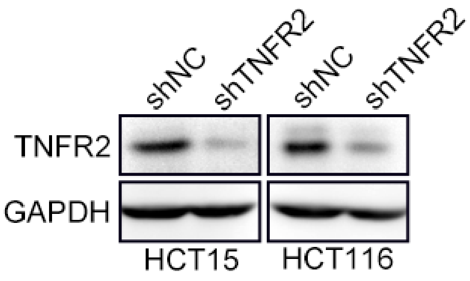
shNC
shTNFR2
shNC
shTNFR2
TNFR2
GAPDH
HCT15
HCT116

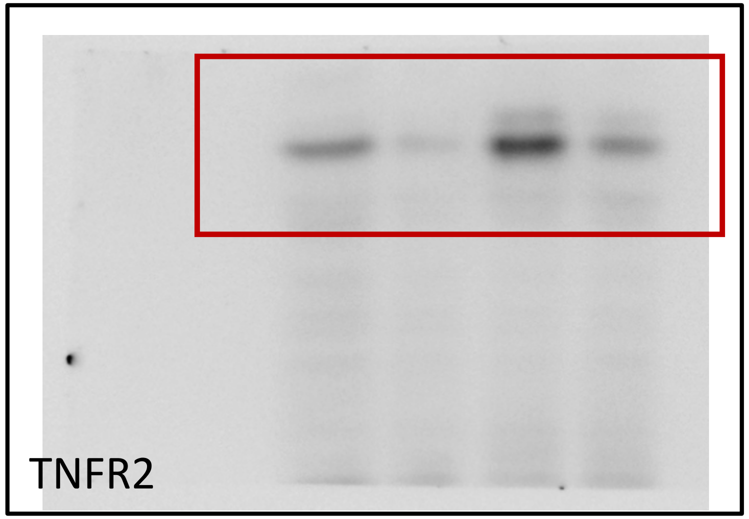
TNFR2

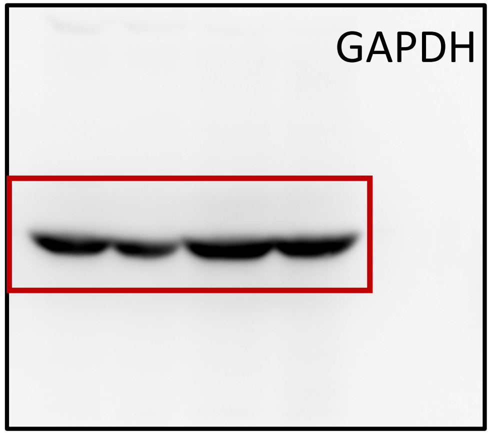
GAPDH

Fig. 1F

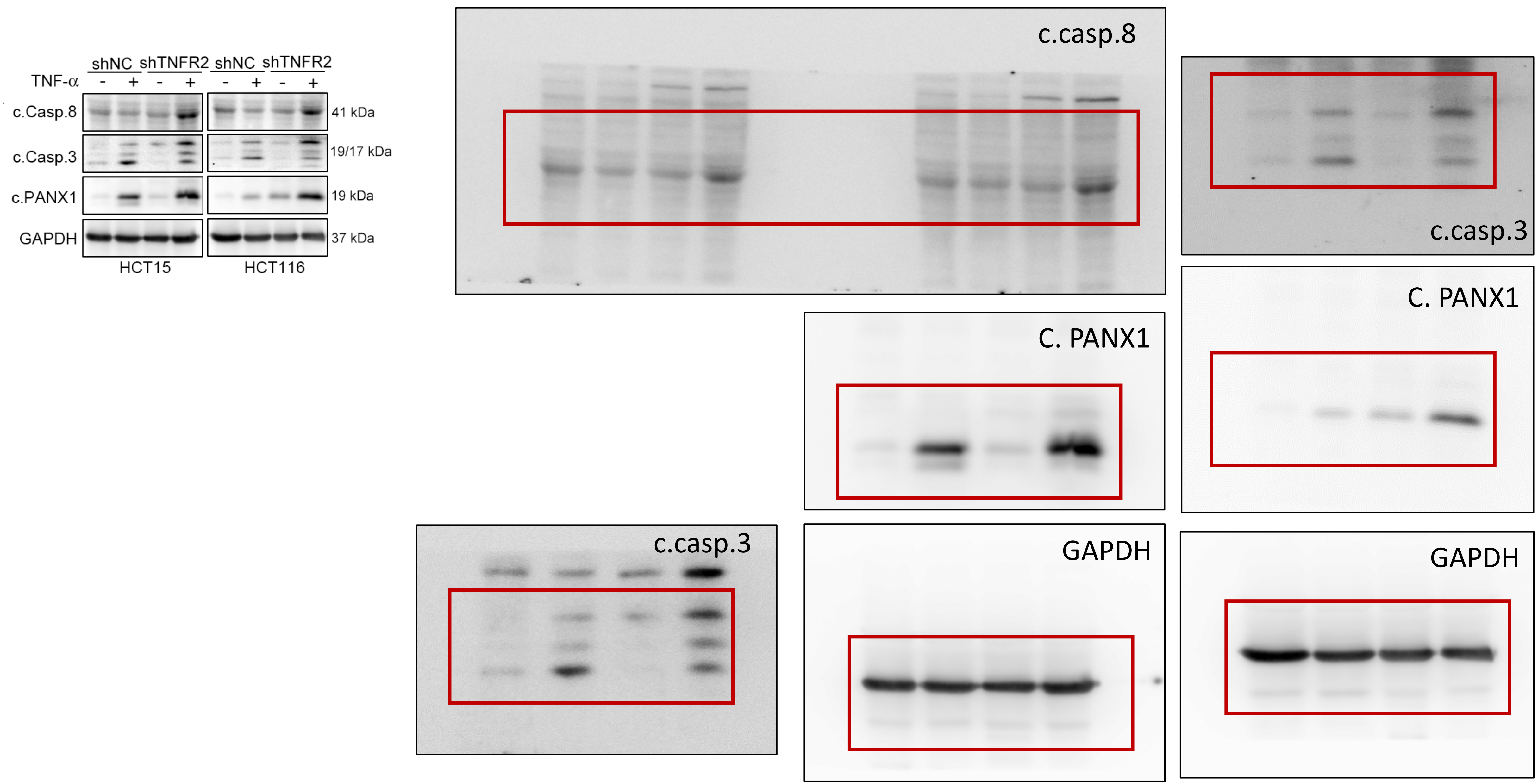

shNC shTNFR2 shNC shTNFR2
TNF-α - + - + - + - +
c.Casp.8
c.Casp.3
c.PANX1
GAPDH
41 kDa
19/17 kDa
19 kDa
37 kDa
HCT15
HCT116
c.casp.8
c.casp.3
C. PANX1
C. PANX1
c.casp.3
GAPDH
GAPDH

Fig. 1G

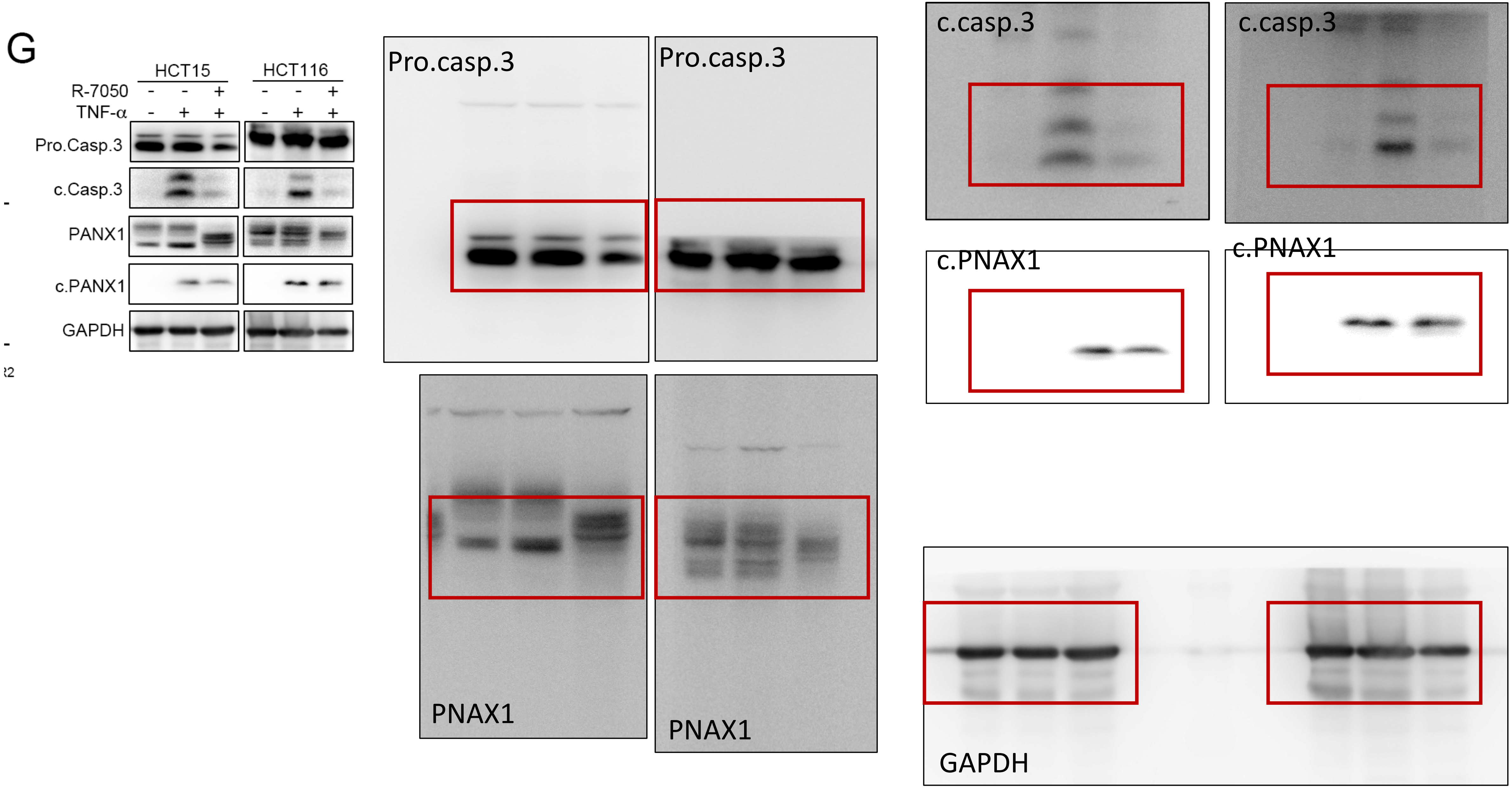

G
HCT15
HCT116
R-7050
TNF-α
Pro.Casp.3
c.Casp.3
PANX1
c.PANX1
GAPDH
Pro.casp.3
Pro.casp.3
c.casp.3
c.casp.3
c.PNAX1
c.PNAX1
PNAX1
PNAX1
GAPDH

Fig. 2A

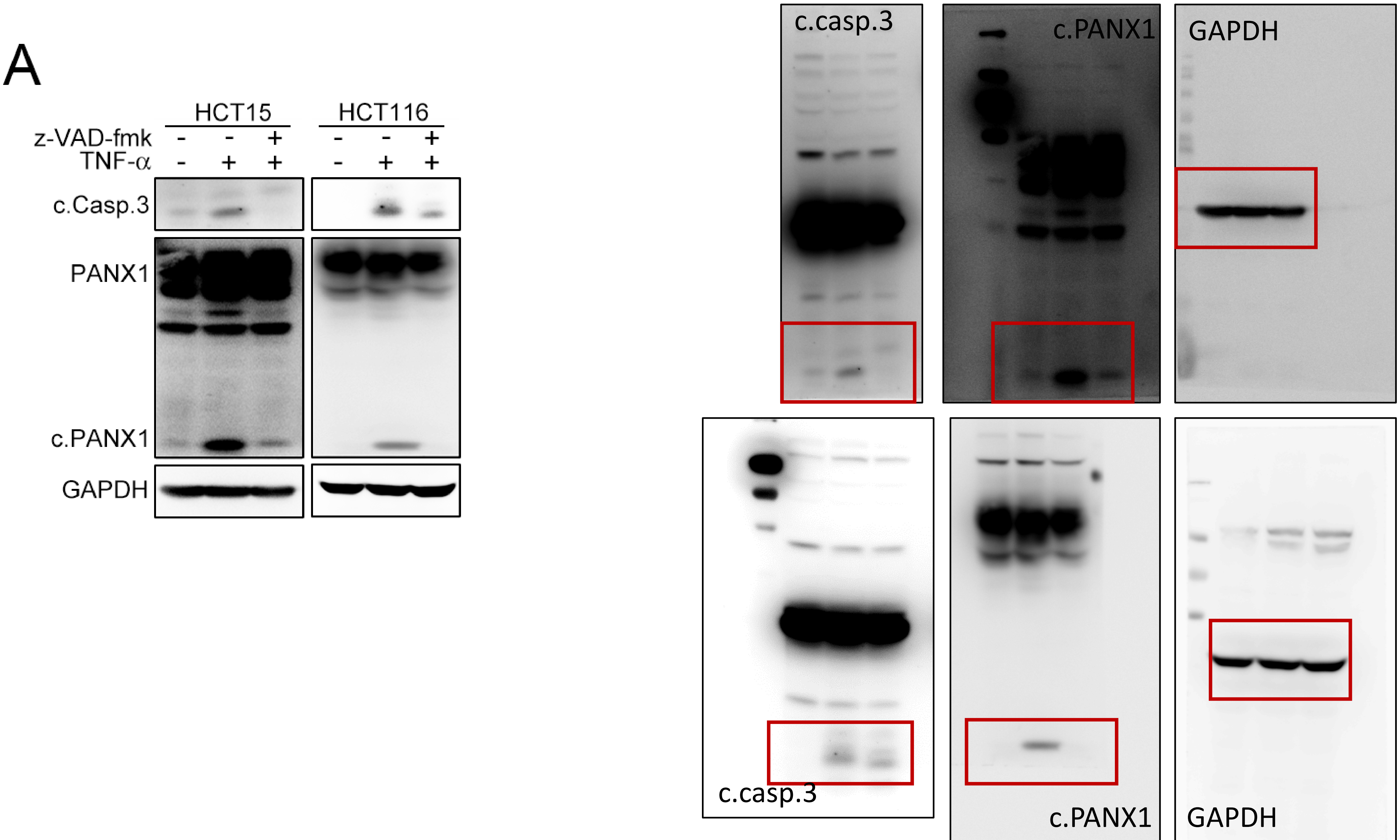
A
HCT15
HCT116
z-VAD-fmk - - + - - +
TNF-α - + + - + +
c.Casp.3
PANX1
c.PANX1
GAPDH
c.casp.3
c.PANX1
GAPDH
c.casp.3
c.PANX1
GAPDH

Fig. 2C

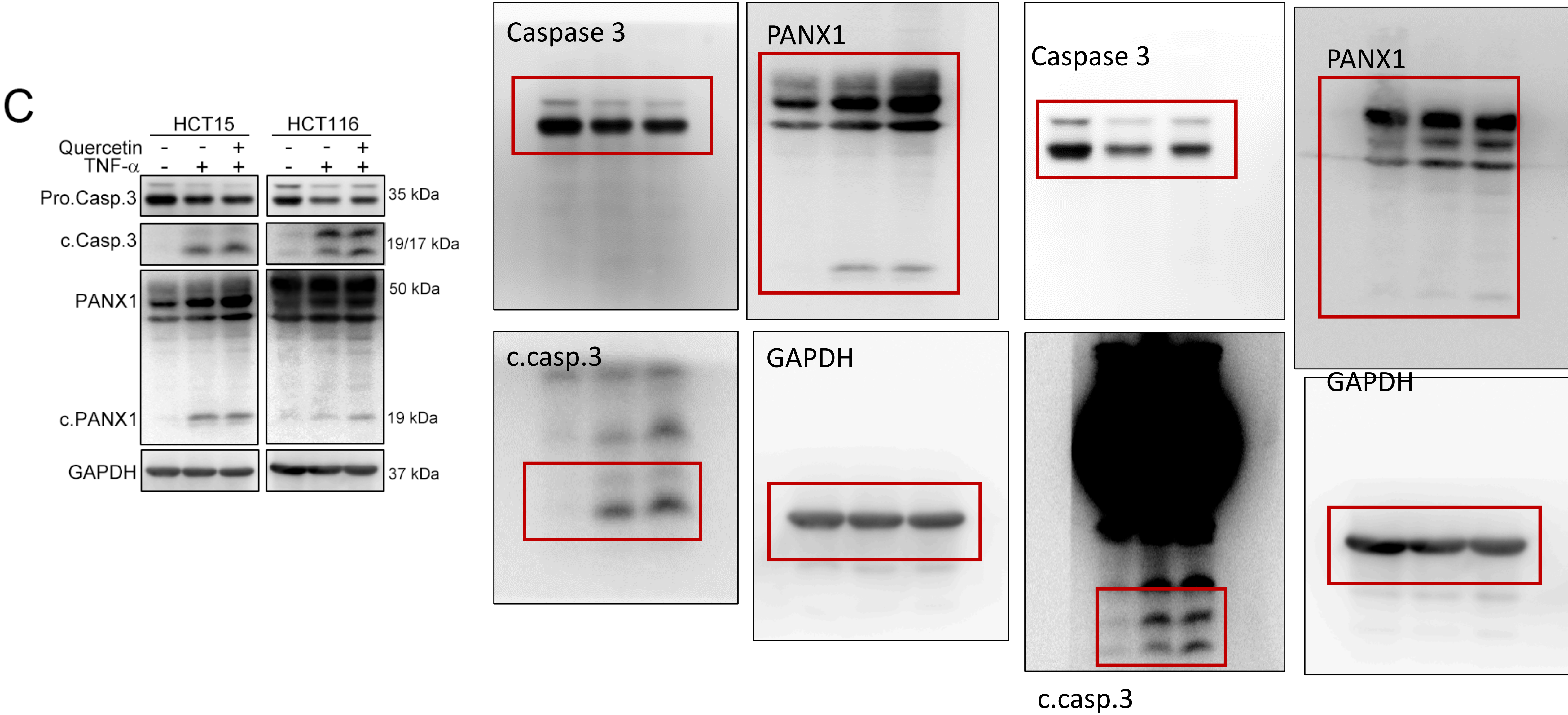

C
HCT15
HCT116
Quercetin
TNF-α
Pro.Casp.3
c.Casp.3
PANX1
c.PANX1
GAPDH
35 kDa
19/17 kDa
50 kDa
19 kDa
37 kDa
Caspase 3
PANX1
c.casp.3
GAPDH
Caspase 3
PANX1
GAPDH
c.casp.3

Fig. 2D

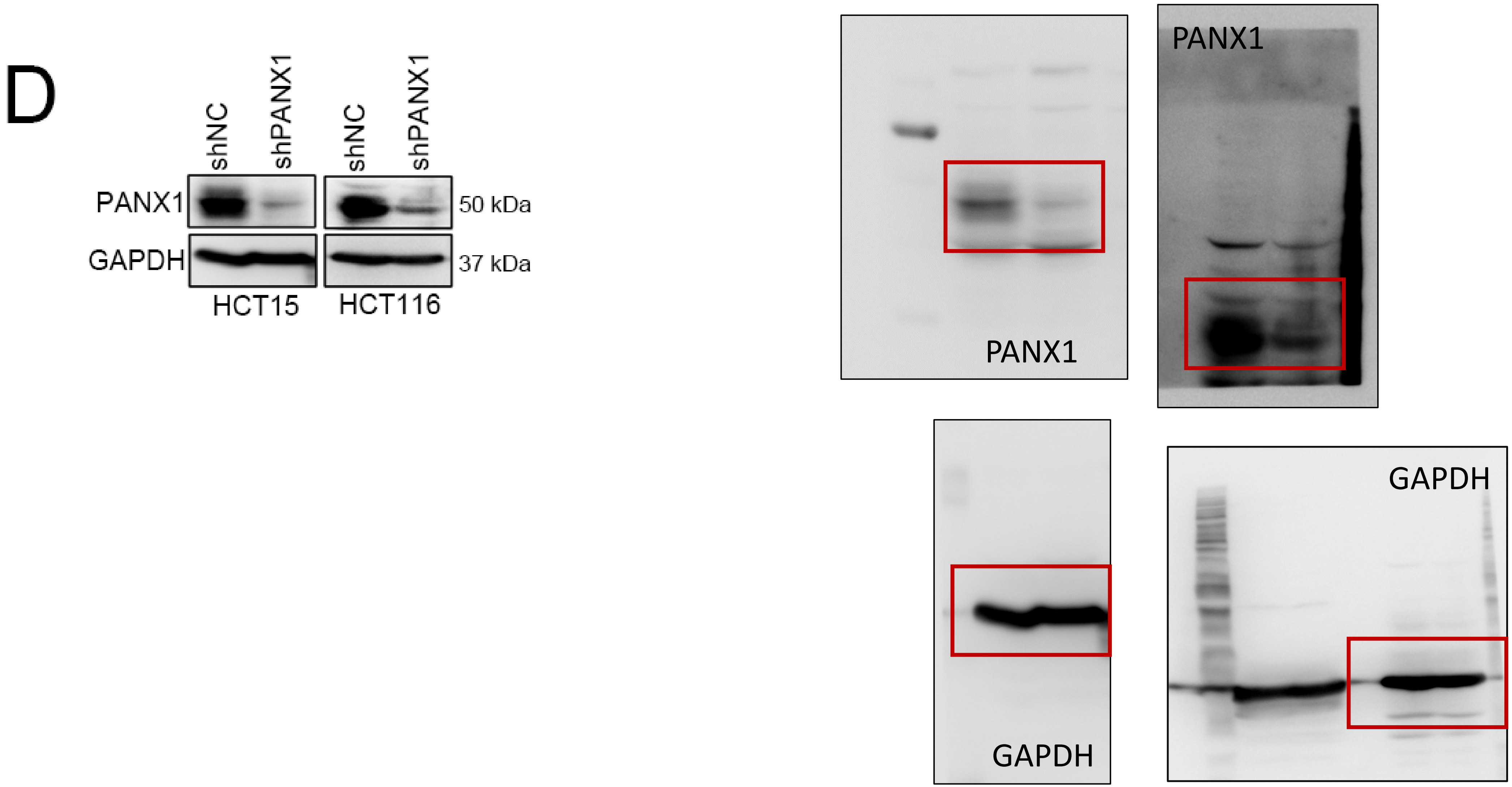
D
shNC
shPANX1
shNC
shPANX1
PANX1
GAPDH
50 kDa
37 kDa
HCT15
HCT116
PANX1
PANX1
GAPDH
GAPDH

Fig. 2E

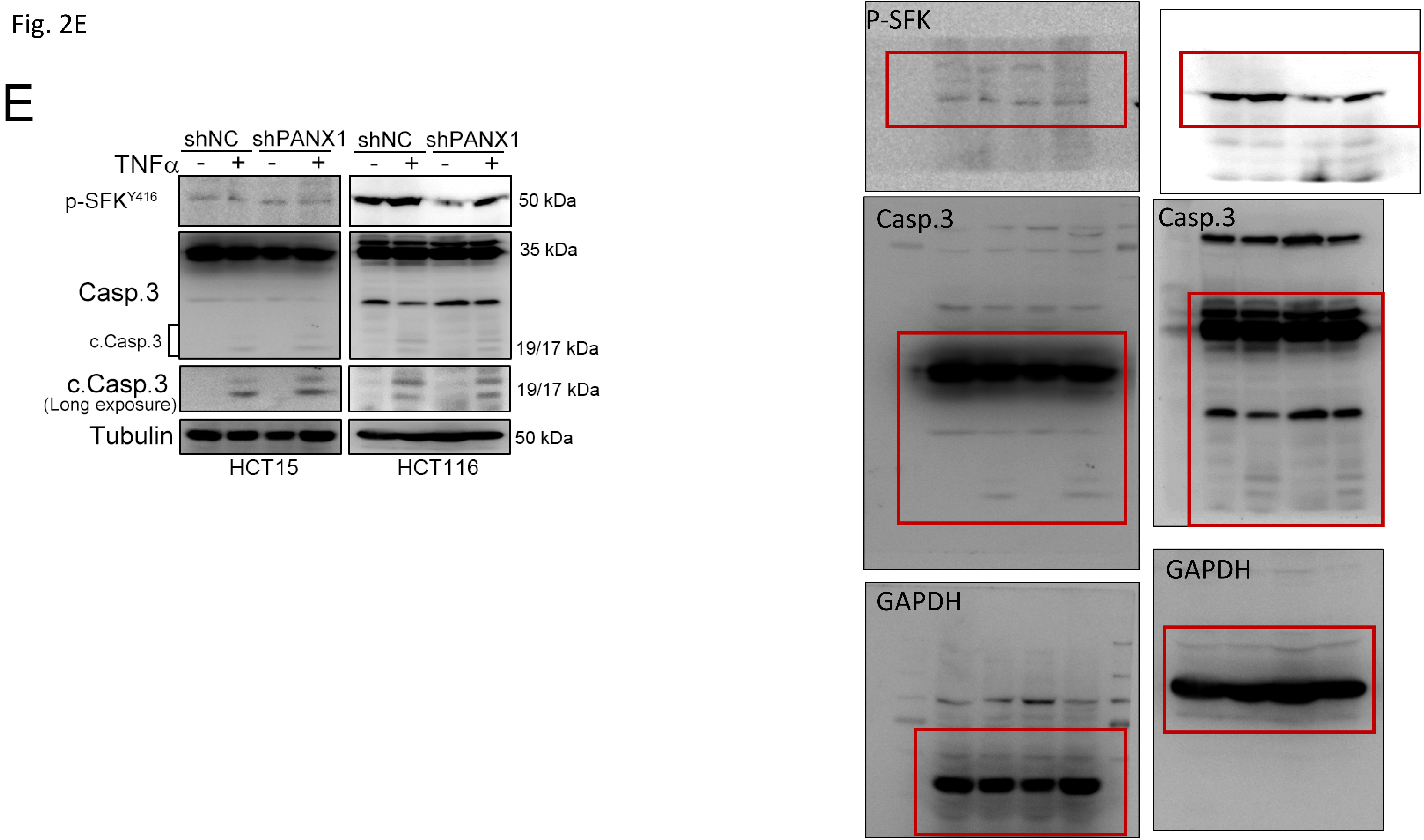

E
shNC
shPANX1
shNC
shPANX1
TNFα - + - + - + - +
p-SFK^Y416
50 kDa
35 kDa
Casp.3
c.Casp.3
19/17 kDa
c.Casp.3
(Long exposure)
19/17 kDa
Tubulin
50 kDa
HCT15
HCT116
P-SFK
Casp.3
Casp.3
GAPDH
GAPDH

Fig. 3C

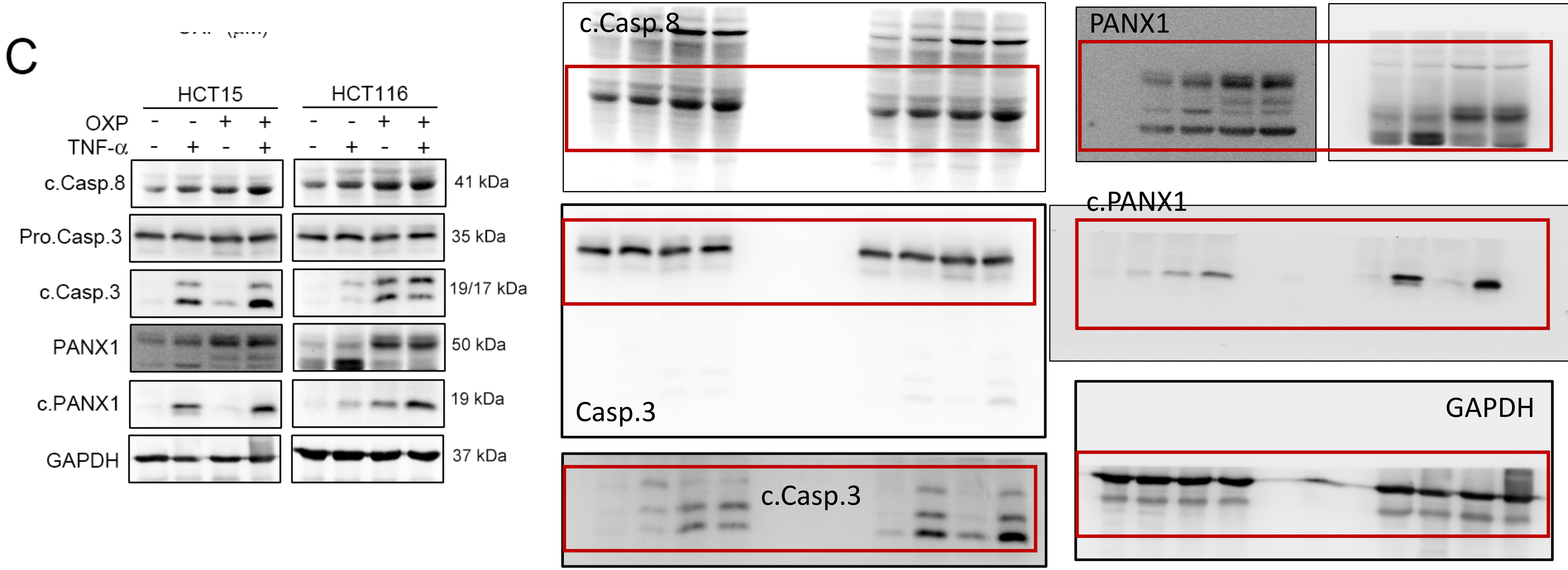
C
HCT15
HCT116
OXP
TNF-α
c.Casp.8
Pro.Casp.3
c.Casp.3
PANX1
c.PANX1
GAPDH
41 kDa
35 kDa
19/17 kDa
50 kDa
19 kDa
37 kDa
c.Casp.8
Casp.3
c.Casp.3
PANX1
c.PANX1
GAPDH

Fig. 4A

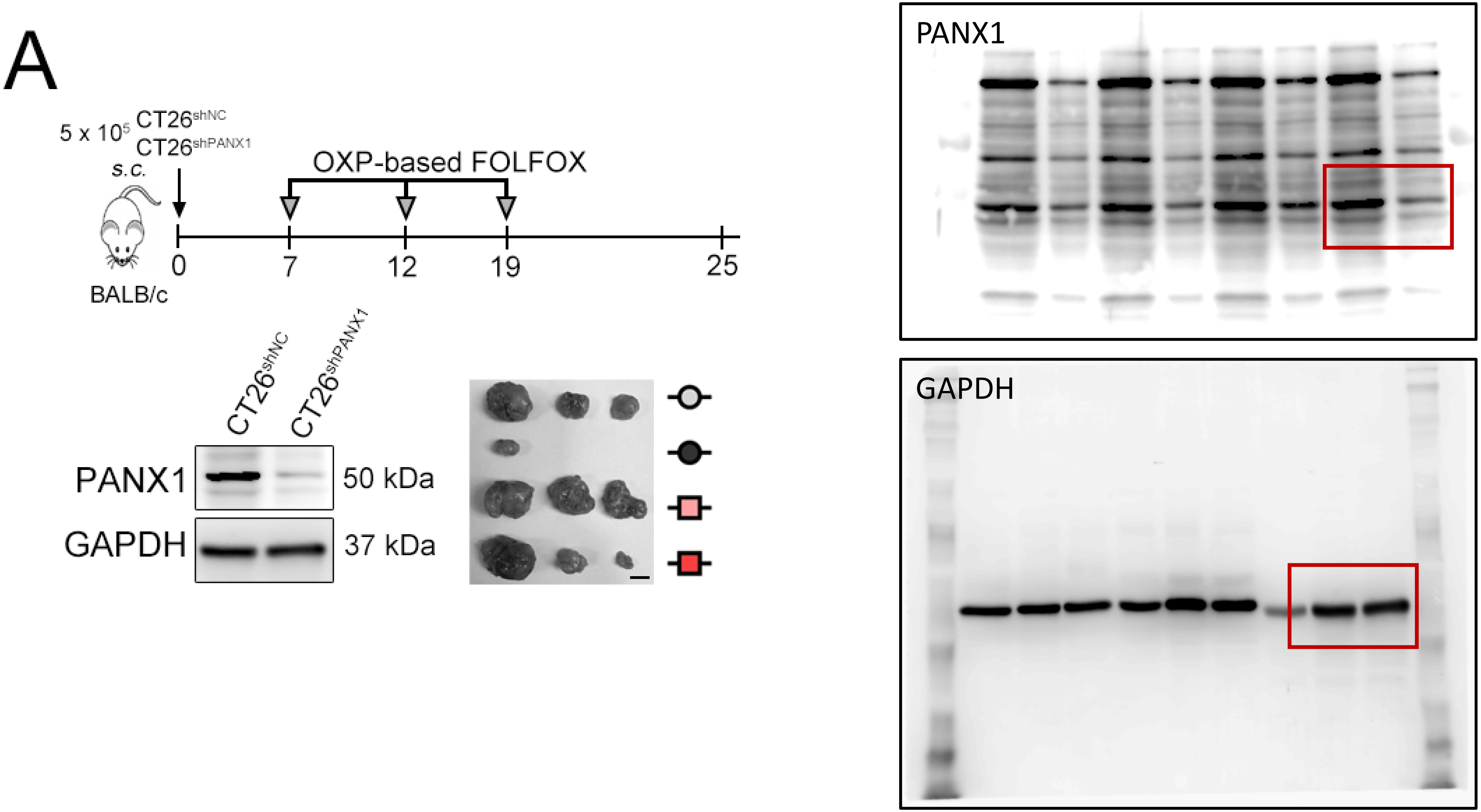
A
5 x 10^5
CT26shNC
CT26shPANX1
s.c.
OXP-based FOLFOX
0
7
12
19
25
BALB/c
CT26shNC
CT26shPANX1
PANX1
50 kDa
GAPDH
37 kDa
PANX1
GAPDH

Fig. 5I

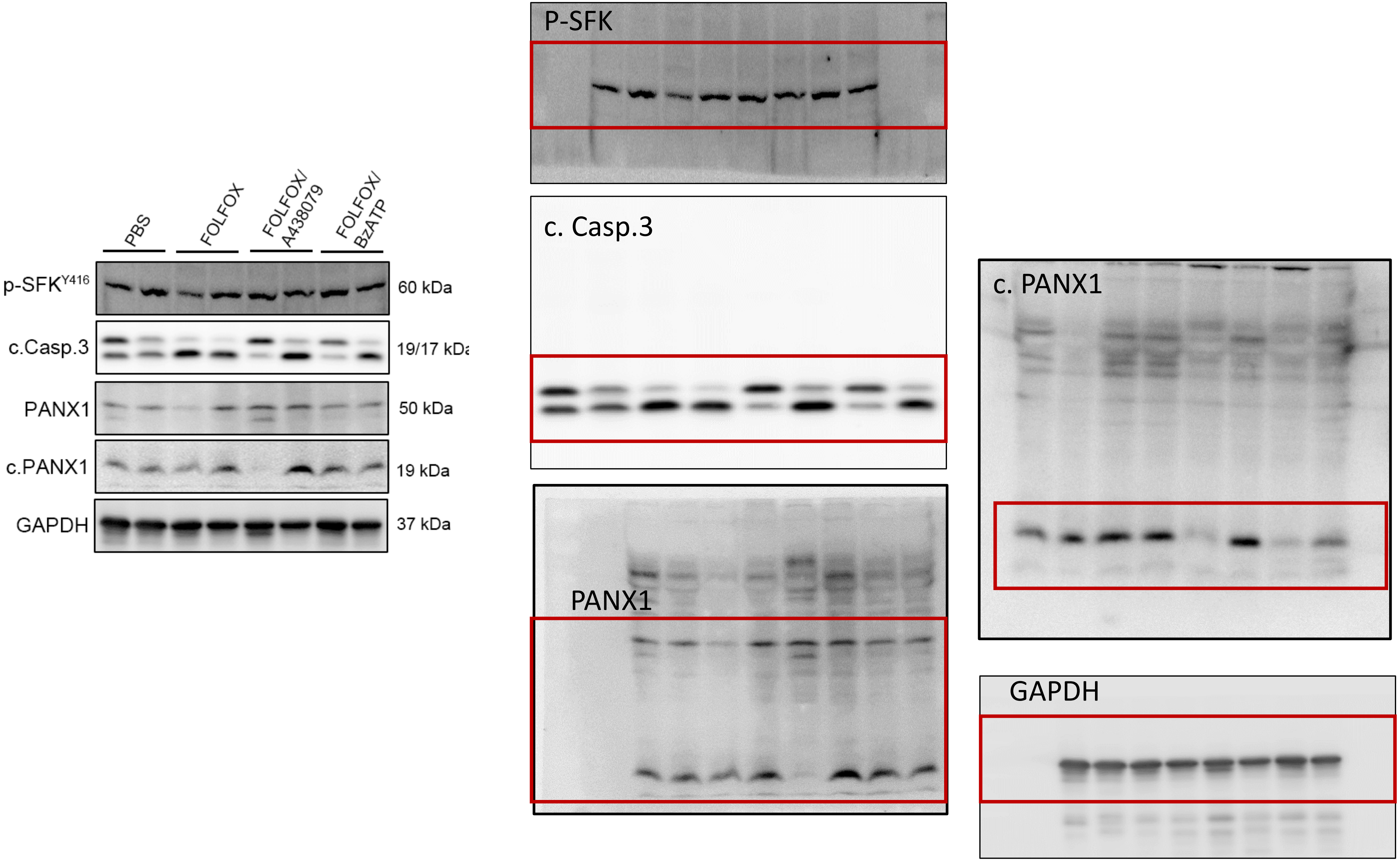
PBS
FOLFOX
FOLFOX/ A438079
FOLFOX/ BzATP
p-SFK^Y416
60 kDa
c.Casp.3
19/17 kDa
PANX1
50 kDa
c.PANX1
19 kDa
GAPDH
37 kDa
P-SFK
c. Casp.3
PANX1
c. PANX1
GAPDH

Fig. S2A

A

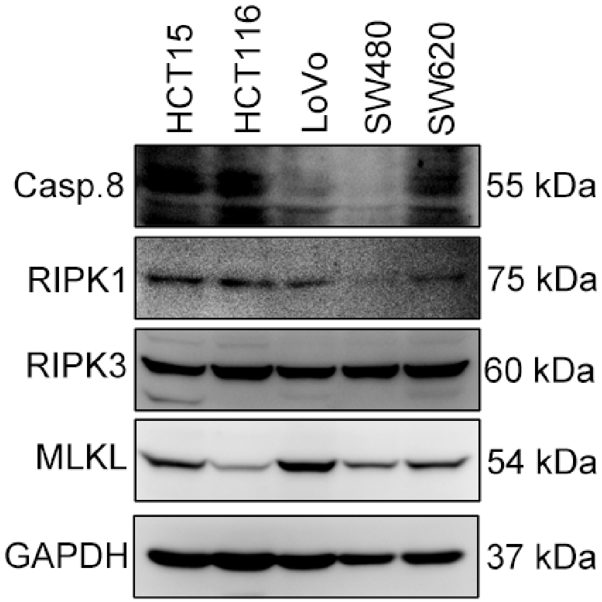
HCT15
HCT116
LoVo
SW480
SW620
Casp.8
55 kDa
RIPK1
75 kDa
RIPK3
60 kDa
MLKL
54 kDa
GAPDH
37 kDa

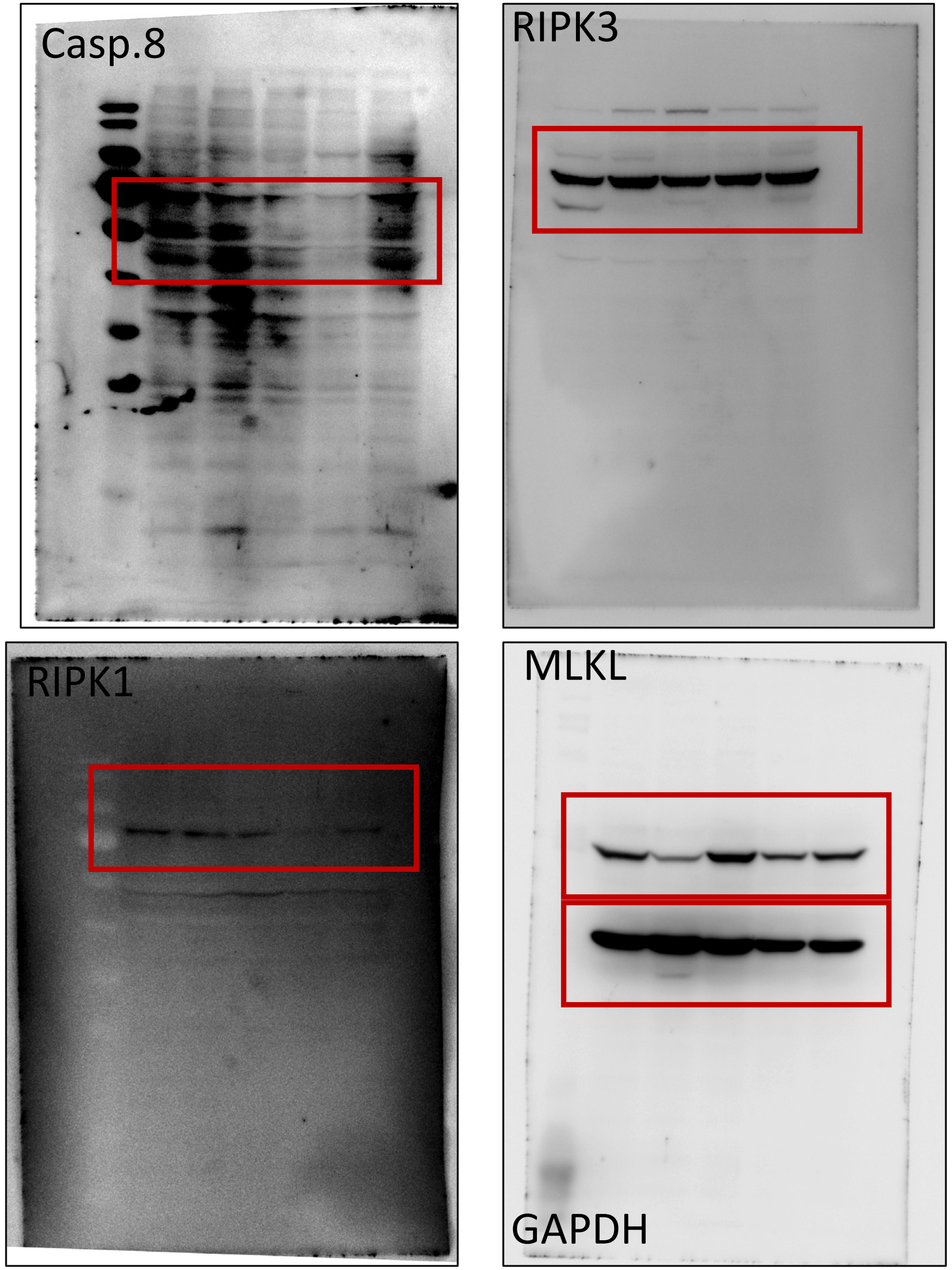
Casp.8
RIPK3
RIPK1
MLKL
GAPDH

Fig. S2B

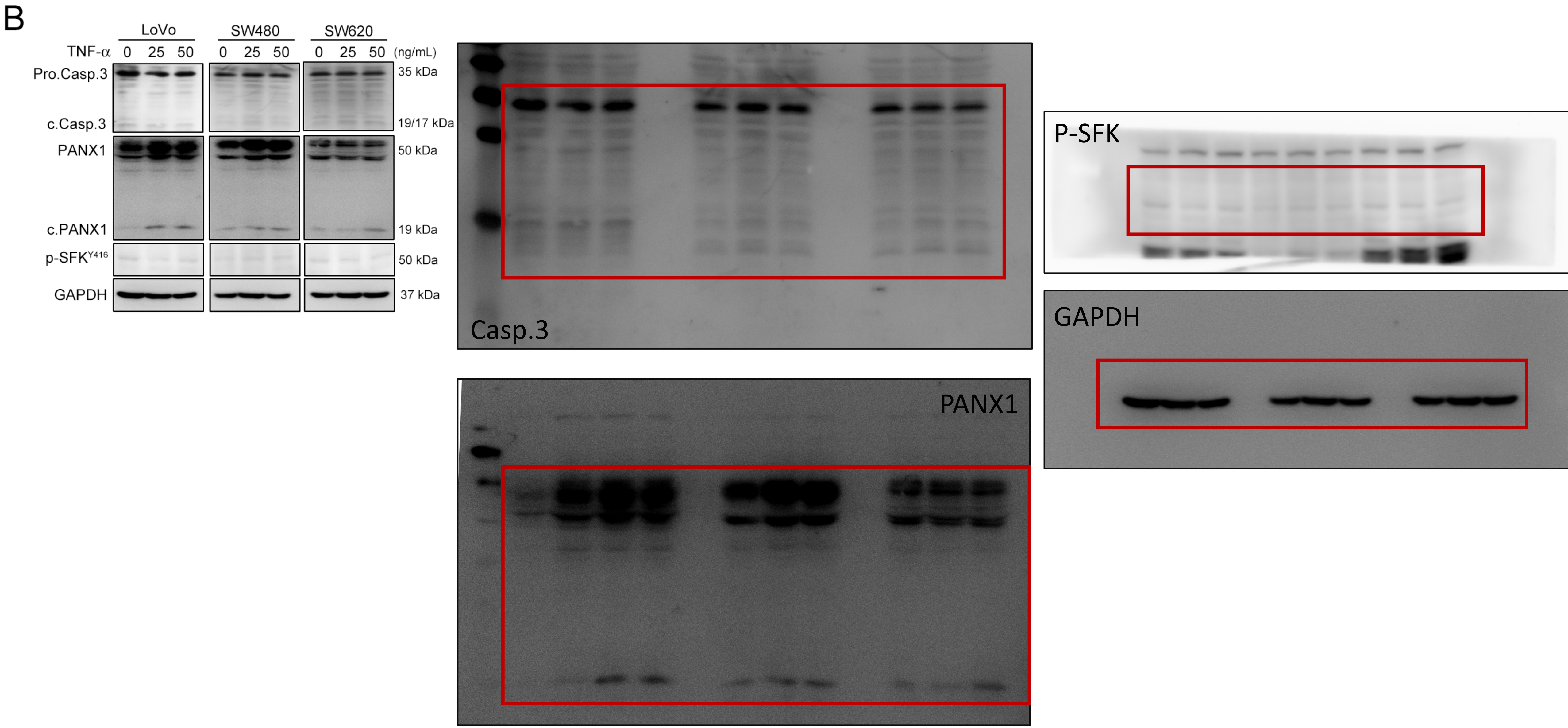
B
LoVo
SW480
SW620
TNF-α
0
25
50
(ng/mL)
Pro.Casp.3
c.Casp.3
PANX1
c.PANX1
p-SFKY416
GAPDH
35 kDa
19/17 kDa
50 kDa
19 kDa
50 kDa
37 kDa
Casp.3
PANX1
P-SFK
GAPDH

Fig. S2C

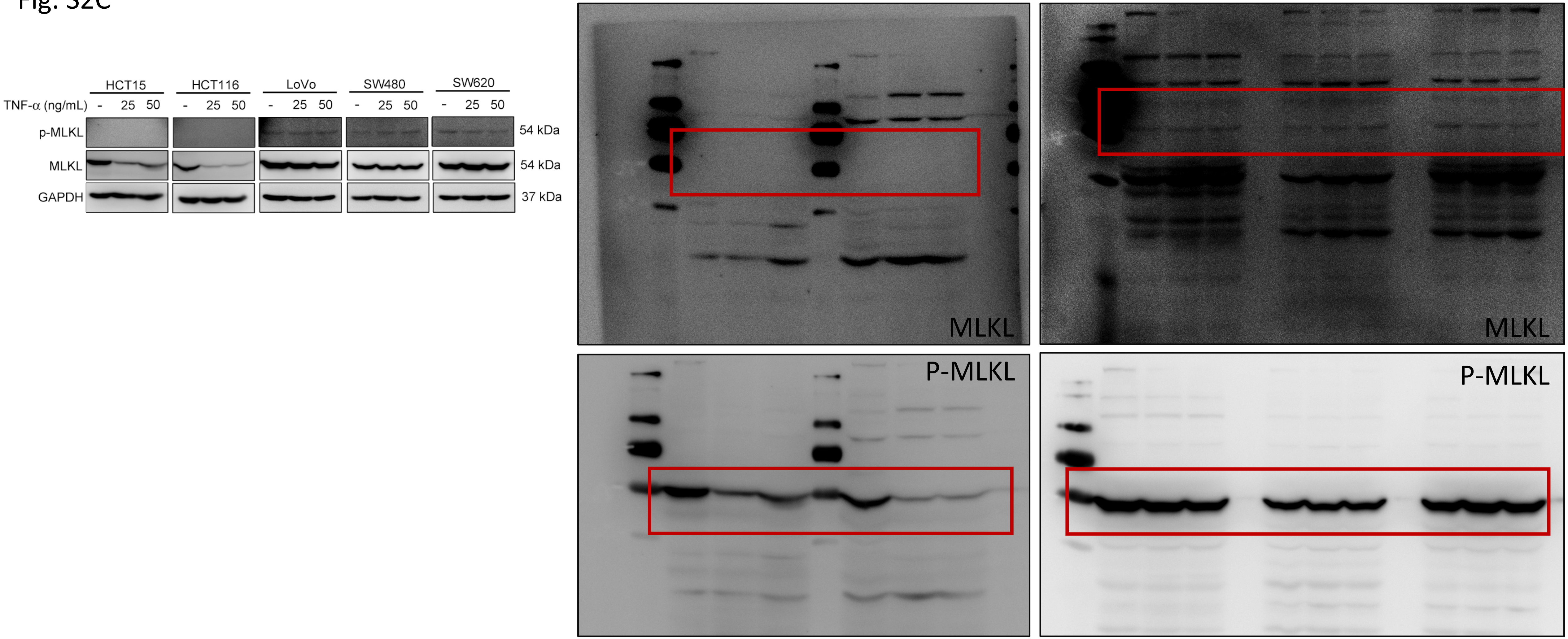
HCT15
HCT116
LoVo
SW480
SW620
TNF-α (ng/mL)
- 25 50 - 25 50 - 25 50 - 25 50 - 25 50
p-MLKL
MLKL
GAPDH
54 kDa
54 kDa
37 kDa
MLKL
MLKL
P-MLKL
P-MLKL

Fig. S2E

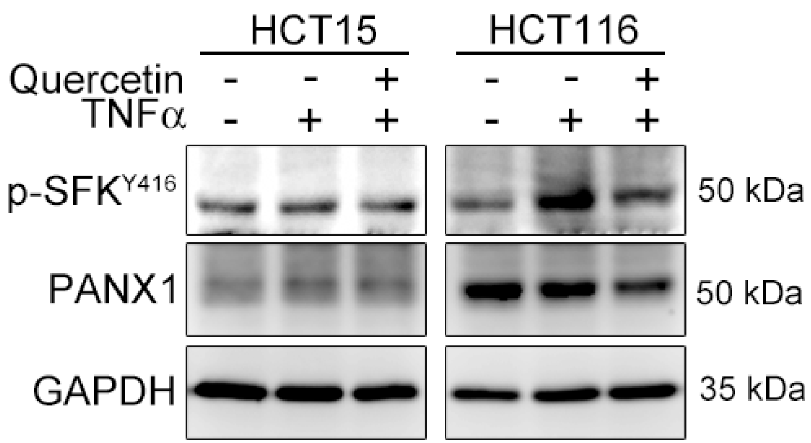
HCT15
HCT116
Quercetin - - + - - +
TNFα - + + - + +
p-SFK Y416
PANX1
GAPDH
50 kDa
50 kDa
35 kDa

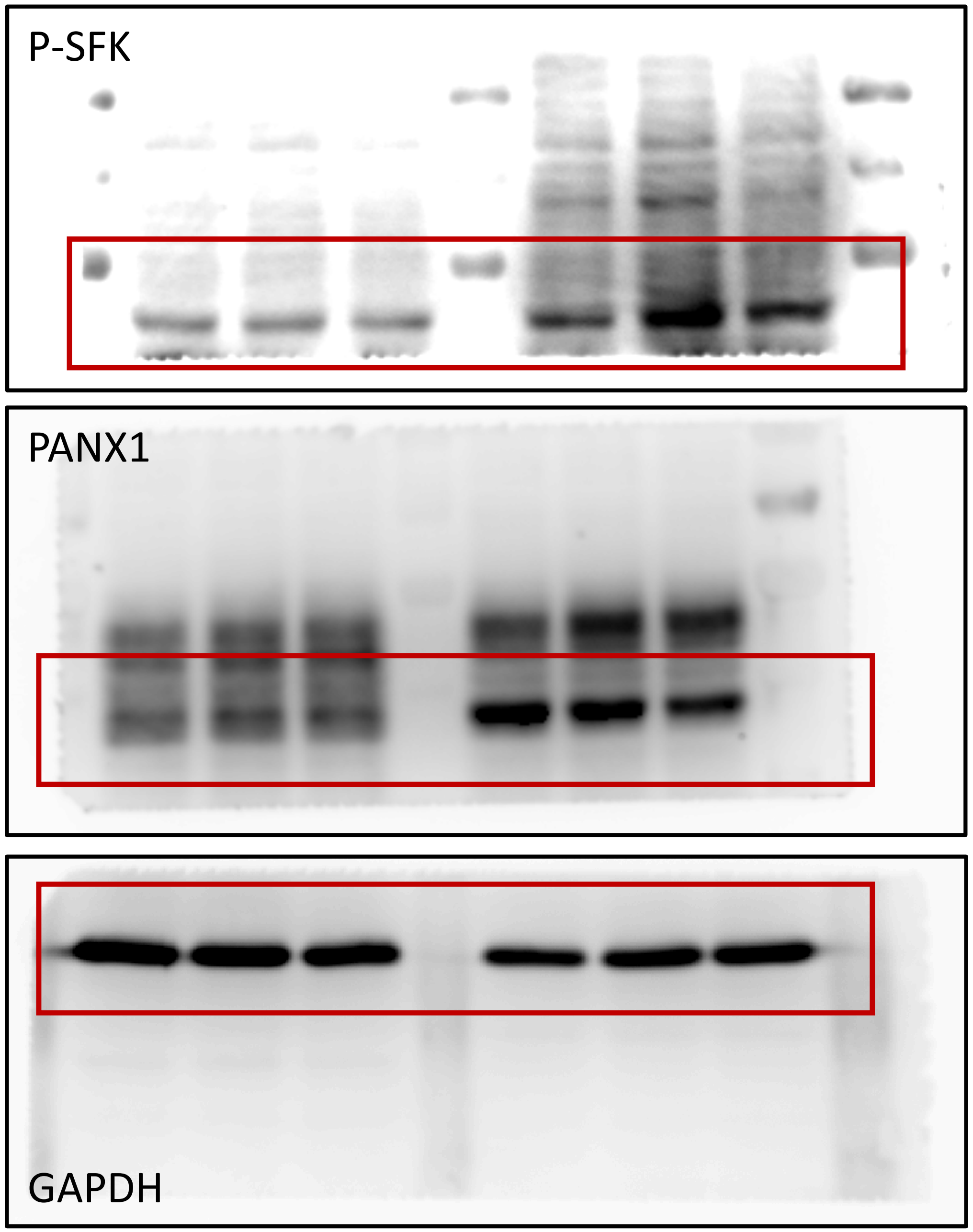
P-SFK
PANX1
GAPDH
